# A hybrid of 1-deoxynojirimycin and benzotriazole induces preferential inhibition of butyrylcholinesterase (BuChE) over acetylcholinesterase (AChE)

**DOI:** 10.1080/14756366.2022.2117912

**Published:** 2022-09-06

**Authors:** Tereza Cristina Santos Evangelista, Óscar López, Adrián Puerta, Miguel X. Fernandes, Sabrina Baptista Ferreira, José M. Padrón, José G. Fernández-Bolaños, Magne O. Sydnes, Emil Lindbäck

**Affiliations:** a Department of Chemistry, Bioscience and Environmental Engineering, Faculty of Science and Technology, University of Stavanger, Stavanger, Norway; b Department of Organic Chemistry, Chemistry Institute, Federal University of Rio de Janeiro, Rio de Janeiro, Brazil; c Departamento de Química Orgánica, Facultad de Química, Universidad de Sevilla, Seville, Spain; d BioLab, Instituto Universitario de Bio-Orgánica “Antonio González” (IUBO-AG), Universidad de La Laguna, La Laguna, Spain

**Keywords:** Iminosugars, Inhibitors, Cholinesterases, Alzheimer’s disease

## Abstract

The synthesis of four heterodimers in which the copper(I)-catalysed azide-alkyne cycloaddition was employed to connect a 1-deoxynojirimycin moiety with a benzotriazole scaffold is reported. The heterodimers were investigated as inhibitors against acetylcholinesterase (AChE) and butyrylcholinesterase (BuChE). The heterodimers displayed preferential inhibition (> 9) of BuChE over AChE in the micromolar concentration range (IC_50_ = 7–50 µM). For the most potent inhibitor of BuChE, Cornish-Bowden plots were used, which demonstrated that it behaves as a mixed inhibitor. Modelling studies of the same inhibitor demonstrated that the benzotriazole and 1-deoxynojirimycin moiety is accommodated in the peripheral anionic site and catalytic anionic site, respectively, of AChE. The binding mode to BuChE was different as the benzotriazole moiety is accommodated in the catalytic anionic site.

## Introduction

**Figure 1.**
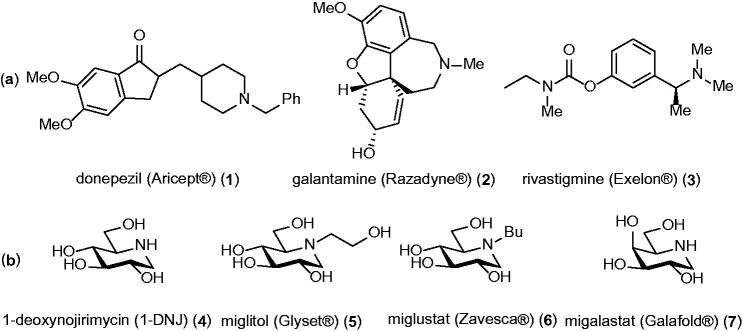
(a) FDA approved ChE inhibitors. (b) Examples of iminosugars.

Alzheimer’s disease (AD) is a neurodegenerative process that constitutes the most common type of dementia, which causes death 3–9 years after diagnosis. AD is a multifactorial disease in which all the factors involved in the progression of the disease have not been pinpointed; however, the two major pathogenic mechanisms are believed to include accumulation of amyloid-beta (Aβ) plaques between the neurons that causes neuronal loss^1^ and formation of neurofibrillary tangles (NFTs) within the neurons that disturbs the synaptic communication between the cells.^2^^,^^3^ The AD brain usually includes additional abnormalities such as inflammation, deficits of the neurotransmitter acetylcholine (ACh), oxidative stress, and metal ion dyshomeostasis.^4^ Currently, there is no fully established therapy available for AD, presumably because all factors involved in the progression of the disease have not yet been identified.^5^ In this context, it is worth mentioning that the Federal Drug Administration (FDA) used its accelerated approval pathway (an approval pathway of drugs against serious diseases that lack other medical options) when it gave green light for aducanumab as an AD drug in 2021.^6^ Aducanumab was approved due to its ability to reduce the level of senile plaque in patients with AD, and thereby being the first approved drug that addresses one of the main pathophysiological hallmarks of AD.^7^ However, no unambiguously improvement of cognition was observed in those patients involved in two separate phase III trials.^6^ Indeed, it has been found that significant levels of senile plaques can be present in the brain of elderly people without cognitive impairments.^8^ Before aducanumab was approved there were only four AD drugs available on the market, which are only able to slow down or delay the symptoms of AD by a few months.^9^ Three out of those palliative drugs, namely donepezil, galantamine, and rivastigmine, are cholinesterase (ChE) inhibitors (Figure 1(a)). Such treatment is in line with the cholinergic hypothesis in which the cognitive decline in AD patients is due to degeneration of neurons in the basal forebrain, which in turn causes loss of neurotransmission in regions of the brain that are essential for the cognitive functions.^10^ Therefore, the purpose of ChE inhibitor drugs is to boost the neurotransmission between neurons by inhibiting the rapid hydrolysis of the neurotransmitter acetylcholine (ACh) in the synaptic cleft by the two major ChEs, acetylcholinesterase (AChE) and butyrylcholinesterase (BuChE).

Iminosugars, such as naturally occurring 1-deoxynojirimycin (1-DNJ) (**4**) (Figure 1(b)) are carbohydrate analogues in which the ring oxygen atom is replaced by a nitrogen atom. Many iminosugars can be partially protonated at physiological pH, in which the conjugate acid can be considered as a charged analogue of the transition state of glycosidase catalysed cleavage of glycosidic bonds.^11^ Therefore, it is not surprising that iminosugars are mostly famous for their properties as glycosidase inhibitors.^12^ The glycosidase inhibitory properties of iminosugars have made them attractive as lead compounds for treatment of diseases such as cancer, diabetes, viral infections, and lysosomal disorders in which carbohydrate processing enzymes are apparent pharmaceutical targets.^13^ The FDA has approved three iminosugars: miglitol (**5**),^14^ miglustat (**6**),^15^ and migalastat (**7**)^16^ for treatment of type 2 diabetes, Gaucher’s disease, and Fabry’s disease, respectively. In addition, in a cellular model of H4APPsw it has been found that miglustat (**6**) lowers the level of Aβ protein,^17^ which is the major component in senile plaque.

X-ray analysis has demonstrated that the overall structure of AChE and BuChE is very similar and that both enzymes host a catalytic triad consisting of histidine, glutamate, and serine almost on the bottom of a *ca.* 20 Å deep active gorge.^18^^,^^19^ Both enzymes contain a tryptophan residue in the catalytic anionic binding site (CAS) nearby the catalytic triad, which establishes π–cation interactions with the cationic ACh substrate and thereby place its ester functional group in a favourable position for hydrolysis in the catalytic triad.^20^ At the mouth of the active site gorge of both enzymes there is an initial substrate binding site, the peripheral anionic binding site (PAS), which is structurally different between AChE and BuChE as the PAS of AChE is more rich in aromatic residues.^21^

**Figure 2.**
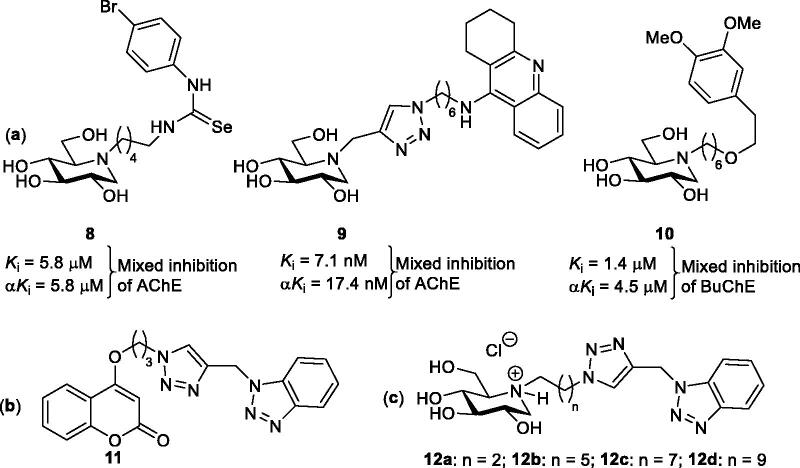
(a) Reported iminosugars ChE inhibitors. (b) Reported AD multifactorial agent candidate. (c) Iminosugar heterodimers evaluated in the current work.

Because iminosugars can be protonated at physiological pH, and as such they were thought to establish cation–π interactions with the tryptophan residue in CAS of AChE in the same way as the quaternary ammonium groups of ACh and of the AChE inhibitors edrophonium and decamethonium.^22^ When a library of 23 iminosugars of different stereochemistry and with substituents in various positions were tested as AChE and BuChE inhibitors, some of them showed inhibition of especially BuChE in the micromolar concentration range.^22^ Dual binding site AChE inhibitors (i.e., such inhibitors that bind simultaneously to PAS and CAS of AChE) are attractive dual action AD drug candidates because they: (1) increase the concentration of the neurotransmitter ACh and (2) inhibit the AChE promoted formation of amyloid fibrils (a component in senile plaques^23^) when the PAS interacts with Aβ-proteins.^24^ Following this line, 1-DNJ (**4**) has been employed as a binding unit in the development of dual binding site ChE inhibitors when it is connected to a second aryl-substituted selenourea (exemplified by **8**),^25^ tacrine (exemplified by **9**),^26^ or catechol binding unit (exemplified by **10**)^27^ (Figure 2(a)). This type of compounds was found to behave as mixed inhibitors of AChE and/or BuChE. The results were interpreted in such a way that heterodimers including a 1-DNJ moiety can bind both to the catalytic site and PAS of BuChE and AChE. Modelling studies of **9** indicated that its 1-DNJ binding unit, in its acidic form, and tacrine binding unit are able to interact simultaneously with the Trp residues in PAS and CAS, respectively.^26^ Modelling studies of **10**, on the other hand, in complex with AChE and BuChE demonstrated that its 1-DNJ binding unit, in its acidic form, and catechol binding unit bind to the catalytic active site and PAS, respectively.^27^

Bedi and co-workers reported a series of coumarin-benzotriazole heterodimers (exemplified by **11** in Figure 2(b)) as multifactorial agents for AD treatment.^28^ The most promising candidate, *viz.* compound **11**, exhibited inhibition potency against AChE and copper-promoted aggregation of Aβ protein. In addition, **11** displays chelation properties with biometal ions (Cu^2+^, Zn^2+^, and Fe^2+^) and is therefore potentially able to tackle metal homeostasis imbalance, which is thought to be involved in the progression of AD. Dimer **11** displayed mixed AChE inhibition, which was consistent with docking studies in which the coumarin and benzotriazole moiety binds to PAS and CAS, respectively. When the benzotriazole ring was connected to a naphthoquinone and anthraquinone system interesting multitarget agents for AD were obtained as they inhibit both ChEs and monoamine oxidase (MAO) B.^29^

As above mentioned, thus far, four papers have been published, which describe the use of iminosugars as ChE inhibitors.^22^^,^^25–27^ Herein, in the fifth paper, we present the synthesis of four heterodimers **12a**–**12d** (Figure 2(c)), which contain 1-DNJ and benzotriazole binding units connected *via* a linker of variable length. The thought behind including two binding units within the same molecule is to establish simultaneously interaction with the catalytic active site and PAS of ChEs. The inhibitory testing demonstrated that **12a**–**12d** exhibit preferential inhibition of BuChE over AChE. To get a better picture of the selectivity, our study also includes modelling studies for the binding of **12b** to AChE and BuChE.

## Materials and methods

### General procedures

DMF, CH_3_CN and CH_2_Cl_2_ were dried over 4 Å molecular sieves (oven dried). The reactions were carried out under an argon atmosphere, unless otherwise specified. Microwave reactions were performed in a CEM Discover-SP, max power 300 W. TLC analyses were performed on Merck silica gel 60 F_254_ plates using UV light for detection. Silica gel NORMASIL 60^®^ 40–63 µm pore size was used for flash column chromatography. NMR spectra were recorded on a Bruker Avance NMR spectrometer; ^1^H NMR spectra were recorded at 400.13 MHz, ^13 ^C NMR spectra were recorded at 100.61 MHz, in CDCl_3_ or CD_3_OD. Chemical shifts are reported in ppm relative to an internal standard of residual chloroform (δ = 7.26 for ^1^H NMR; δ = 77.16 for ^13 ^C NMR) and residual methanol (δ = 3.31 for ^1^H NMR; δ = 49.00 for ^13 ^C NMR). High-resolution mass spectra (HRMS) were recorded on a Qexactive spectrometer in positive electrospray ionisation (ESI) mode.

### Synthetic protocols

#### General procedure for the preparation of compounds 18a–d

To a solution of iminosugar **17** (0.30 mmol, 1 equiv.) in dry acetonitrile (15 ml) were added the corresponding alkyl dibromide (12 equiv.) and potassium carbonate (1.3 equiv.). The resulting mixture was heated under microwave irradiation at 100 °C for 1 h. The reaction mixture was then cooled to room temperature and the solvent was evaporated under reduced pressure, followed by addition of 10 ml of water and extraction with EtOAc (2 × 20 ml). The combined organic layers were dried (MgSO_4_), filtered and concentrated under reduced pressure. The residue was purified by silica gel flash column chromatography (See Supplementary Material).

#### General procedure for the synthesis of compounds 19a–d

To a solution of the alkyl bromide **18a**, **18 b**, **18c,** or **18d** (0.22 mmol, 1 equiv.) in DMF (2 ml) was added NaN_3_ (4 equiv.). The reaction mixture was heated overnight at 45 °C under Ar-atmosphere. The mixture was then cooled to room temperature and the solvent was evaporated under reduced pressure; 10 ml of water was then added, and the aqueous phase was extracted with CH_2_Cl_2_ (2 × 20 ml). The combined organic layers were dried (MgSO_4_), filtered and concentrated under reduced pressure without the need for further purification (See Supplementary Material).

#### General procedure for the synthesis of compounds 21a–d

To a mixture of alkyne **20** (0.2 mmol, 1 equiv.), azide **19a**, **19b**, **19c,** or **19d** (0.2 mmol, 1 equiv.), and copper (II) sulphate pentahydrate (0.4 equiv.) in DMF (4 ml) in a foil covered round bottom flask was added sodium ascorbate (0.8 equiv.). The mixture was kept stirring at room temperature under Ar-atmosphere overnight. The solvent was then removed under reduced pressure, and the concentrate was suspended in water (10 ml) and EtOAc (20 ml). The layers were separated, and the organic extract was dried (MgSO_4_), filtered, and concentrated under reduced pressure. The residue was purified by silica gel flash column chromatography (See Supplementary Material).

#### General procedure for the synthesis of compounds 12a–d

A solution of compound **21a**, **21b**, **21c,** or **21d** (0.12 mmol, 1 equiv.) in anhydrous CH_2_Cl_2_ (10 ml) under an Ar-atmosphere at −78 °C was slowly added BCl_3_ (1 M in heptane, 33 equiv.). After addition, the mixture was kept stirring at −78 °C for 2 h and then at 0 °C overnight. The volatiles were then removed under reduced pressure and the concentrate underwent purification by silica gel column chromatography (See Supplementary Material).

#### Inhibition assays

The inhibitory activity of **12a–12d** against AChE and BuChE was carried out following minor modifications of the Ellman assay,^30^ as reported previously^31^ using Thermo Scientific^TM^ Varioskan^TM^ LUX microplate reader and Greiner F-bottom 96-well plates.

## General method for docking simulations

Structures for all proteins (AChE: PDBid 4EY7; BuChE: PDBid 6QAA) were retrieved from the Protein DataBank.^32^ Crystal structures were optimised using the QuickPrep protocol from MOE (Chemical Computing Group). All ligands were drawn, hydrogens added, and the geometry was optimised with MOE. For the docking calculations, performed with MOE, in the placement stage, we used the Triangle Matcher algorithm with the London dG scoring scheme. In the refinement stage, the receptor was kept rigid, and the GBVI/WSA dG scoring scheme was used.

## Synthesis

**Scheme 1.**
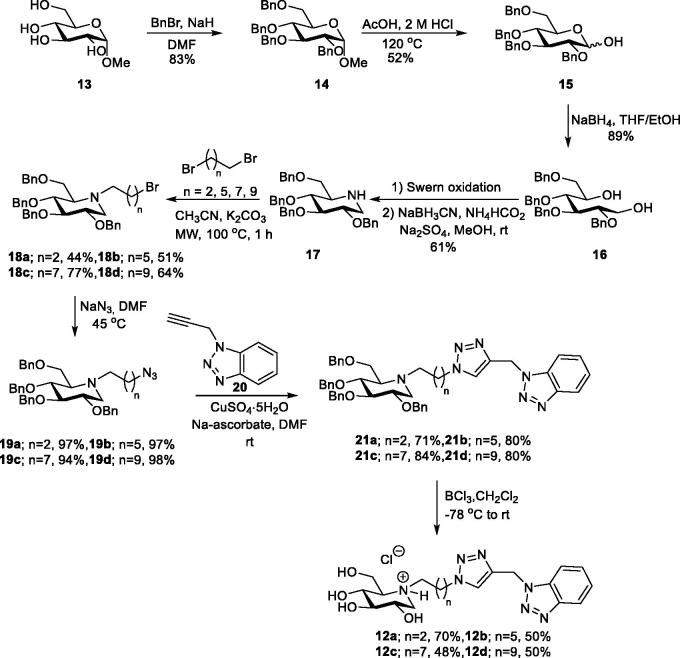
Synthesis of target compounds **12a**–**12d**.

The synthesis of 1-DNJ-benzotriazole heterodimers **12a**–**12d** commenced from commercially available methyl α-D-glucopyranoside (**13**) (Scheme **1**), which was converted into tetra-*O-*benzylated 1-DNJ **17** by following a five-step sequence reported by Wennekes et al. including: (1) per-*O*-benzylation, (2) acidic cleavage of glycosidic bond, (3) reduction of pyranose **15**, (4) double Swern oxidation of diol **16**, and (5) double reductive amination to obtain **17**.^33^ *N*-alkylation of **17** *via* a nucleophilic substitution reaction is challenging when carried out under conventional heating,^34^ presumably due to the steric hindrance on the NH-group exerted by the 6-*O*-benzyl group.^35^ One solution for the *N*-alkylation challenge of **17** is to use microwave irradiation. Thus, bromoalkyl derivatives **18a–18d** of 1-DNJ were obtain in 44–77% when **17** was treated with an excess of dibromoalkanes in acetonitrile in the presence of K_2_CO_3_ under microwave irradiation. Treatment of **18a**–**18d** with sodium azide provided the azide cycloaddition partners **19a**–**19d** in excellent yield with no need for purification before they underwent copper(I)-catalysed azide-alkyne cycloaddition (CuAAC) with *N*-propargyl benzotriazole **20**^28^ to afford heterodimers **21a**–**21d** in 71–84% yield. BCl_3_ promoted de-*O*-benzylation of **21a**–**21d** provided the target compounds **12a**–**12d.**

## Cholinesterase inhibition studies

The inhibition potencies of 1-DNJ-benzotriazole heterodimers **12a**–**12d** were tested against *ee*AChE (AChE from *Electrophorus electricus*) and *eq*BuChE (BuChE from equine serum) by using minor modification of the Ellman assay.^30^ The minimum inhibitory concentrations required to achieve 50% inhibition (IC_50_) of the ChE enzymes are tabulated in Table 1. The test series includes galantamine (**2**) as a positive reference and parent 1-DNJ (**4**) to assess the impact of the benzotriazole binding group on the inhibitory properties of **12a**–**12d**.

**Table 1.** IC_50_ values for the inhibition of *ee*AChE and *eq*BuChE by heterodimers **12a**–**12d**.

Compound		IC_50_(μM)^a^			BuChE selectivity^d^
		n	*ee*AChE^b^	*eq*BuChE^c^	
1-DNJ (**4**)	**4**		>100^[26]^	10 ± 0	>10
Galantamine (**2**)	**2**		1.29 ± 0.14	5.47 ± 0.40	0.24
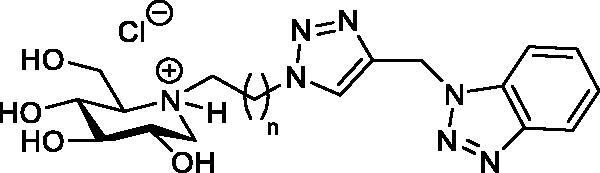	**12a**	2	>500	50 ± 3	>10
	**12b**	5	65 ± 2	6.7 ± 0.7	9.7
	**12c**	7	>500	26 ± 0.7	>19
	**12d**	9	>500	17 ± 0.7	>19

^a^ Mean ± SD.

^b^ [S] = 121 µM.

^c^ [S] = 112 µM.

BuChE selectivity = IC_50_(AChE)/IC_50_(BuChE).

As shown in Table 1, 1-DNJ together with heterodimers **12a**, **12c**, and **12d** exhibited very poor inhibition potency against AChE. Heterodimer **12b** was an outlier from this triplet of heterodimers as it displayed inhibition in the micromolar concentration range, which shows that the arming of 1-DNJ with a benzotriazole group can strengthen its AChE inhibition potency when the number of CH_2_-groups in the linker is optimised.

When testing for activity towards BuChE that includes an active site gorge with a larger volume compared to AChE and different combination of residues, which affect the inhibitor selectivity of such enzymes,^36^ all heterodimers, **12a**–**12d**, became inhibitors in the micromolar concentration range (IC_50_ = 7–50 μM) with a clear preference for BuChE over AChE (BuChE selectivity ≥ 9.7). From Table 1, it is obvious that arming 1-DNJ with a benzotriazole binding group can both attenuate and increase its BuChE inhibitory potency. In fact, **12b** is a 1.5-fold more potent BuChE inhibitor than 1-DNJ. Heterodimers **12a**, **12c**, and **12d**, on the other hand, are 1.7 up to 5 times less potent BuChE inhibitors than 1-DNJ.

**Figure 3.**
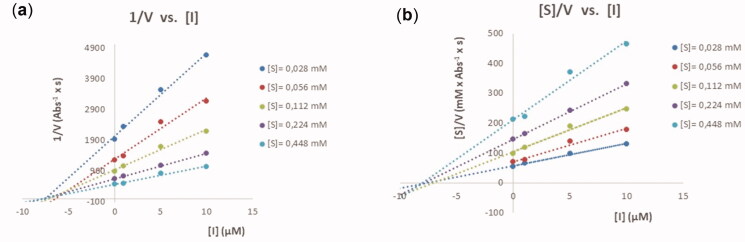
Cornish-Bowden plots for analysing the mode of inhibition of **12b**.

Even though IC_50_-values provide useful information about inhibition potency they do not provide any information about the type of inhibition and affinity for inhibitors to their enzymes.^37^ Therefore, two plots (1/V *vs.* [I] and [S]/V *vs.* [I]) were created for the inhibition of BuChE by **12b** (Figure 3). Since an intersection point is present in both plots, it was concluded that **12b** behaves as a mixed inhibitor against BuChE.^38^ The mixed inhibition type was interpreted as **12b** has the capacity to bind both to PAS and the active site of BuChE. However, because the competitive inhibition constant (*K*_i_ = 4.9 ± 0.40 μM) is smaller than the uncompetitive inhibition constant (α*K*_i_ = 12 ± 1 μM), it is reasonable to expect that **12b** possesses a greater affinity for the active site than for PAS.

## Docking studies

**Figure 4.**
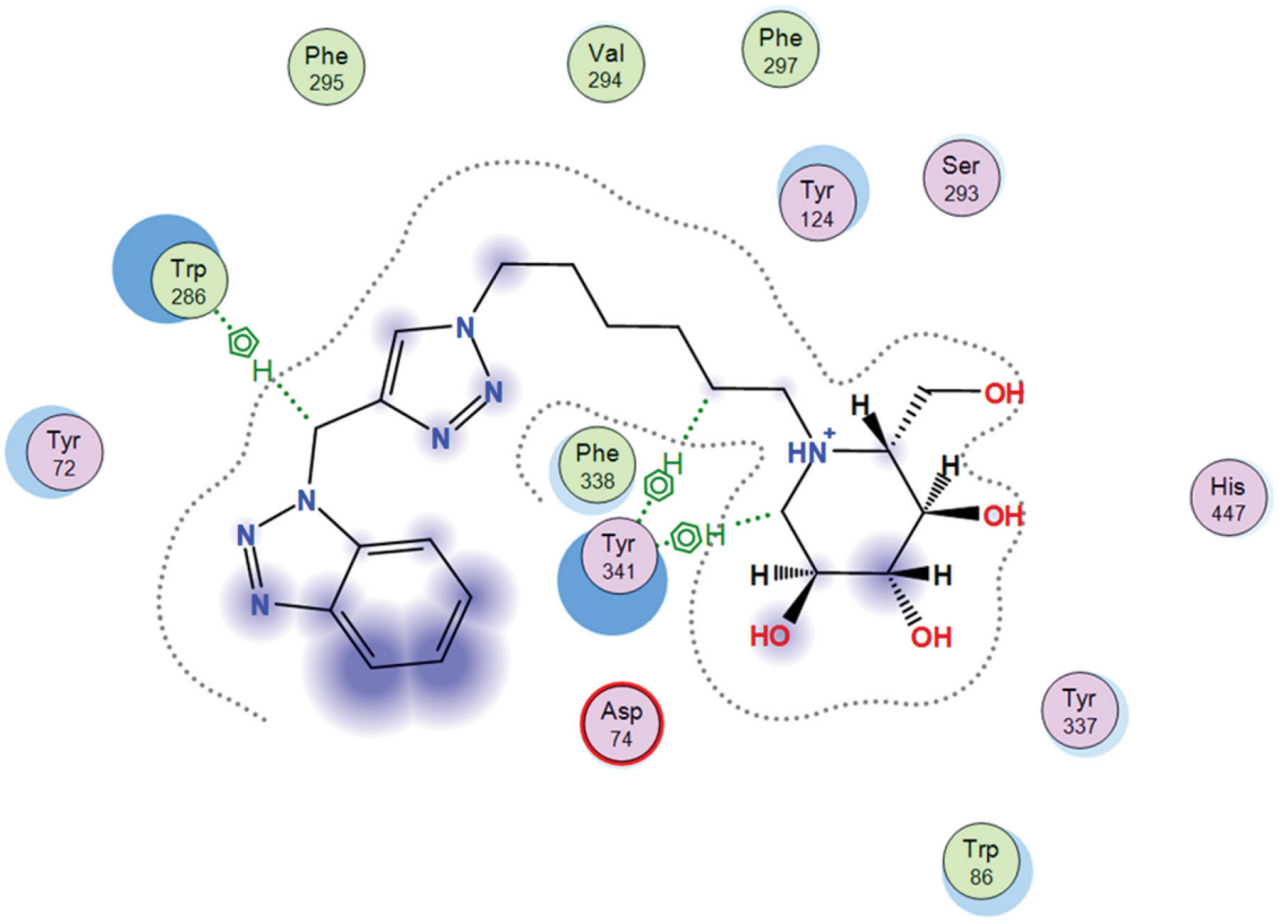
Most energetically favoured binding pose of **12b** to *rh*AChE.

The preferred binding pose of **12b** in recombinant human acetylcholinesterase (*rh*AChE) is shown in Figure 4, in which the benzotriazole and iminosugar moiety is accommodated in PAS and CAS, respectively. The most striking result, given that **12b** binds in its acidic form, is that no cation–π interactions are observed between the positively charged nitrogen atom of **12b** and any of the aromatic residues in the active gorge. However, because increased polarity of a X-H bond increases the strength of a X − H···π interaction,^39^ it is possible that the positively charged nitrogen atom of **12b** further polarise the C–H bonds of the neighbouring endocyclic CH_2_-group and thereby strengthen its CH–π interaction with Tyr341. In addition, the CH_2_-group between the benzotriazole and triazole moiety was observed to participate in a CH–π interaction with Trp286 in PAS.

**Figure 5.**
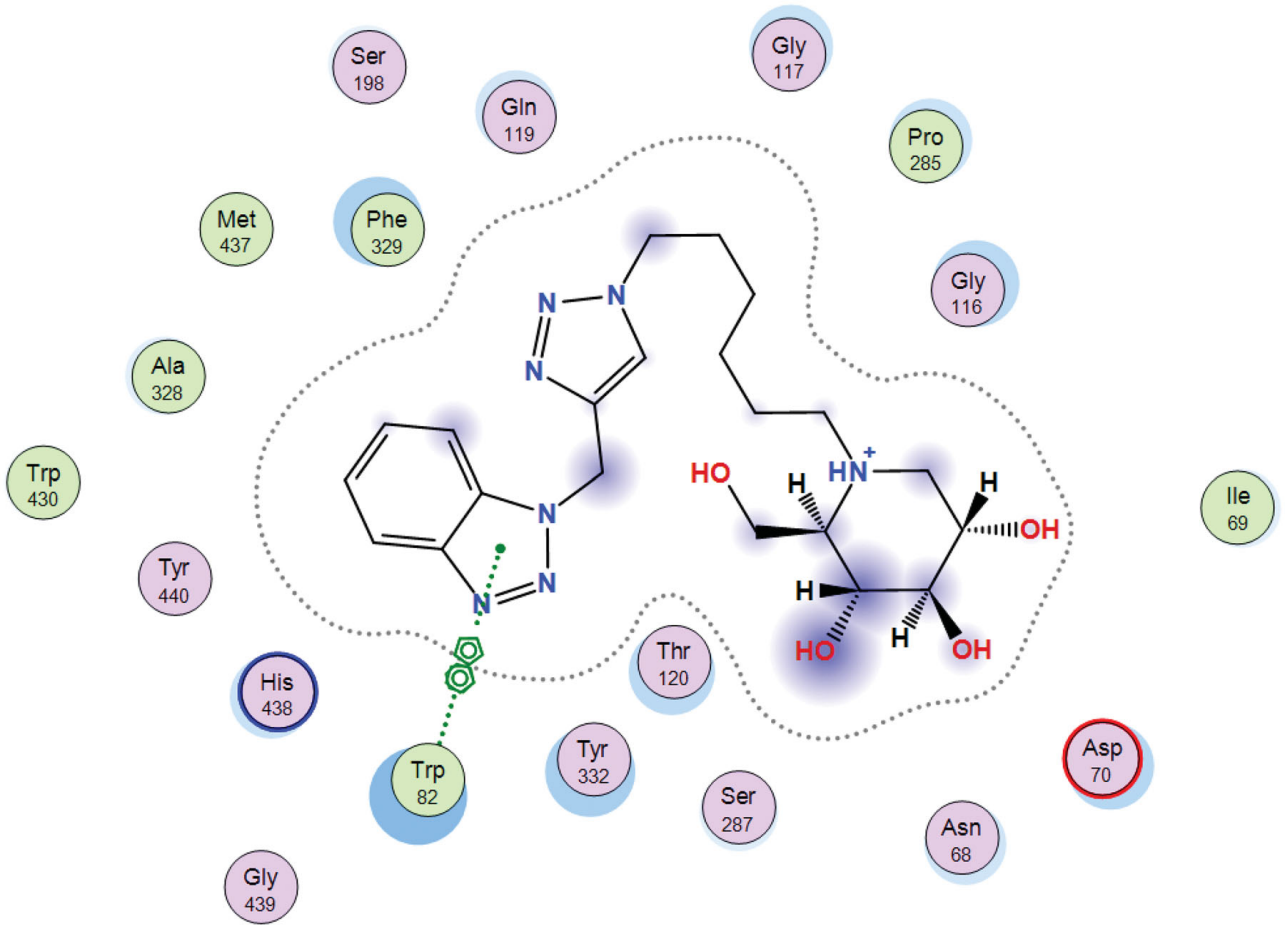
Preferred binding pose of **12b** to *h*BuChE.

The most stable binding pose of **12b** to the active gorge of human BuChE (*h*BuChE) is presented in Figure 5. The binding mode of **12b** to AChE and BuChE is very different as the benzotriazole moiety of **12b** is accommodated in the CAS of BuChE in which it participates in π–π interactions with the Trp82 moiety.

## Conclusions

Heterodimers **12a**–**12d** are stronger inhibitors for BuChE than for AChE. The inhibition of BuChE by **12a**–**12d** is susceptible to the number of CH_2_-groups between the 1-DNJ and benzotriazole binding groups as **12b** is a more potent BuChE inhibitor than 1-DNJ whereas **12a**, **12c**, and **12d** are less potent than 1-DNJ. Modelling studies suggest that the 1-DNJ binding group is placed in the PAS of BuChE and the benzotriazole binding group in the CAS. This suggested dual binding site inhibition is supported by Cornish-Bowden plots, which demonstrate that **12b** exhibit mixed type of inhibition against BuChE. The hydroxyl groups of heterodimers **12a**–**12d** open the gate to prepare derivatives, which include additional pharmacophores and thereby target more of the factors that are involved in the progression of AD. Indeed, such studies are currently in progress in our laboratories.

## Supplementary Material

Supplemental Material
