# Supplementary material for: A hybrid of 1-deoxynojirimycin and benzotriazole induces preferential inhibition of butyrylcholinesterase (BuChE) over acetylcholinesterase (AChE)
Source: J Enzyme Inhib Med Chem. 2022 Sep 6;37(1):2395–402. doi: 10.1080/14756366.2022.2117912 (PMC9467581; doi:10.1080/14756366.2022.2117912)

Supplementary Information

A hybrid of 1-deoxynojirimycin and benzotriazole induces preferential inhibition of butyrylcholinesterase (BuChE) over acetylcholinesterase (AChE)

Tereza Cristina Santos Evangelista,[a,b] Óscar López,[c] Adrián Puerta,[d] Miguel X. Fernandes,[d] Sabrina Baptista Ferreira,[b] José M. Padrón,[d] José G. Fernández-Bolaños,[c] Magne O. Sydnes,[a] Emil Lindbäck[a*]

[a]*Department of Chemistry, Bioscience and Environmental Engineering, Faculty of Science and Technology, University of Stavanger, Stavanger, Norway*

[b]*Department of Organic Chemistry, Chemistry Institute, Federal University of Rio de Janeiro, Rio de Janeiro, Brazil*

[c]*Departamento de Química Orgánica, Facultad de Química, Universidad de Sevilla, Seville, Spain.*

[d]*BioLab, Instituto Universitario de Bio-Orgánica "Antonio González" (IUBO-AG), Universidad de La Laguna, c/Astrofísico Francisco Sánchez 2, La Laguna, E-38206, Spain*

Table of Contents

1. 2,3,4,6-Tetra-O-benzyl-D-glucitol (16) ***3***

2. 2,3,4,6-Tetra-O-benzyl-1-deoxynojirimycin (17) ***3***

3. Compounds 18a-d **4**

3.1. *N*-(3-bromopropyl)-2,3,4,6-tetra-*O*-benzyl-1-deoxynojirimycin **(18a)** 4
3.2. *N*-(6-bromohexyl)-2,3,4,6-tetra-*O*-benzyl-1-deoxynojirimycin **(18b)** 5
3.3. *N*-(8-bromooctyl)-2,3,4,6-tetra-*O*-benzyl-1-deoxynojirimycin **(18c)** 5
3.4. *N*-(10-bromodecyl)-2,3,4,6-tetra-*O*-benzyl-1-deoxynojirimycin **(18d)** 6

4. Compounds 19a-d **6**

4.1. *N*-(3-azidopropyl)-2,3,4,6-tetra-*O*-benzyl-1-deoxynojirimycin **(19a)** 6
4.2. *N*-(6-azidohexyl)-2,3,4,6-tetra-*O*-benzyl-1-deoxynojirimycin **(19b)** 7
4.3. *N*-(8-azidooctyl)-2,3,4,6-tetra-*O*-benzyl-1-deoxynojirimycin **(19c)** 7
4.4. *N*-(10-azidodecyl)-2,3,4,6-tetra-*O*-benzyl-1-deoxynojirimycin **(19d)** 8

5. 1-(2-Propyn-1-yl)-1*H*-benzotriazole (20) **8**

6. Compounds 21a-d **9**

6.1.1. *N*-(1-(2-((1H-benzo[1,2,3]triazol-1-yl)methyl)-1*H*-1,2,3-triazol-1-yl)propyl)-2,3,4,6-tetra-*O*-benzy-1-deoxynojirimycin **(21a)** 9
6.1.2. *N*-(1-(2-((1H-benzo[1,2,3]triazol-1-yl)methyl)-1*H*-1,2,3-triazol-1-yl)hexyl)-2,3,4,6-tetra-*O*-benzy-1-deoxynojirimycin **(21b)** 10
6.1.3. *N*-(1-(2-((1*H*-benzo[1,2,3]triazol-1-yl)methyl)-1*H*-1,2,3-triazol-1-yl)octyl)-2,3,4,6-tetra-*O*-benzy-1-deoxynojirimycin **(21c)** 10
6.1.4. *N*-(1-(2-((1H-benzo[1,2,3]triazol-1-yl)methyl)-1*H*-1,2,3-triazol-1-yl)decyl)-2,3,4,6-tetra-*O*-benzy-1-deoxynojirimycin **(21d)** 11

7. Compounds 12a-d **12**

7.1. *N*-(1-(2-((1*H*-benzo[1,2,3]triazol-1-yl)methyl)-1*H*-1,2,3-triazol-1-yl)propyl)-1-deoxynojirimycin hydrochloride **(12a)** 12
7.2. *N*-(1-(2-((1*H*-benzo[1,2,3]triazol-1-yl)methyl)-1*H*-1,2,3-triazol-1-yl)hexyl)-1-deoxynojirimycin hydrochloride **(12b)** 12
7.3. *N*-(1-(2-((1*H*-benzo[1,2,3]triazol-1-yl)methyl)-1*H*-1,2,3-triazol-1-yl)octyl)-1-deoxynojirimycin hydrochloride **(12c)** 13
7.4. *N*-(1-(2-((1*H*-benzo[1,2,3]triazol-1-yl)methyl)-1*H*-1,2,3-triazol-1-yl)decyl)-1-deoxynojirimycin hydrochloride **(12d)** 13

8. References ***14***

9. NMR Spectra ***15***

1. **2,3,4,6-Tetra-*O*-benzyl-D-glucitol (16)**

NaBH$_4$ (0.50 g, 0.01 mol, 2 equiv.) was added in portions to a solution of pyranose **15** (3.55 g, 6.57 mmol, 1 equiv.) in 1:1 EtOH/THF (70 mL) in a round bottom flask at 0 °C. Then, the mixture was kept stirring at room temperature overnight. After this time, the solvent was evaporated under reduced pressure and the resulting residue was suspended in 100 mL of ethyl acetate. 50 mL of 0.5 M aqueous HCl solution was slowly added to the suspension at 0°C. The phases were separated, and the organic phase was washed with saturated $NaHCO_3$ solution (100 mL). The organic phase was dried over $MgSO_4$, filtered and concentrated under reduced pressure. The residue was purified by silica gel flash column chromatography (PE/EtOAc 70:30 → 50:50) to obtain **16** (3.18 g, 89%) as a colorless oil. R_f 0.34 (EP/EtOAc 1:1); $[\alpha]_D^{25} + 13$ (*c* 1.70, CH_2Cl_2); The observed NMR data are in agreement with reported data:[1] ^{1}H-NMR δH ($CDCl_3$; 400.13 MHz) 7.36-7.28 (18H, m, Ar-H), 7.25-7.22 (2H, m, Ar-H), 4.74-4.62 (4H, m, 4xCH-Ph), 4.59-4.50 (4H, m, 4xCH-Ph), 4.07-4.03 (1H, m, 2-H), 3.92-3.90 (1H, m, 4-H), 3.83-3.73 (3H, m, 6a-H, 4-H, 5-H), 3.66-3.65 (2H, m, 1-H), 3.58 (1H, dd, *J* = 11.8 Hz, *J* = 4.6 Hz, 6b-H); ^{13}C-NMR δC ($CDCl_3$, 100.61 MHz) 138.3 (Ar), 138.1 (Ar), 138.0 (Ar), 137.9 (Ar), 128.6-127.9 (Ar), 79.6 (3-C), 79.2 (4-C), 77.4 (5-C), 74.6 (CH_2Ph), 73.6 (CH_2Ph), 73.4 (CH_2Ph), 73.2 (CH_2Ph), 71.2 (2-C), 70.8 (1-C), 62.0 (6-C).

2. **2,3,4,6-Tetra-*O*-benzyl-1-deoxynojirimycin (17)**

The synthesis of **17** was carried out by following a protocol reported by Overkleeft and coworkers:[1]

Step 1: Swern Oxidation

A solution of oxalyl chloride (1.9 mL, 22.3 mmol, 4 equiv.) in 22 mL of anhydrous CH_2Cl_2 was cooled to -78ºC, followed by dropwise addition of a solution of anhydrous DMSO (2.0 mL, 27.9 mmol, 5 quiv.) in 14 mL of anhydrous CH_2Cl_2. The mixture was stirred for 40 min at -78 ºC. Then, a solution of diol **16** (3.03 g, 5.59 mmol, 1 equiv. in 11 mL of dry CH_2Cl_2) was added dropwise to the reaction mixture at -78 ºC. After stirring for 2 h at -78 ºC, Et_3N (9.4 mL, 67.1 mmol, 12 equiv.) was added dropwise. After addition, the mixture was allowed to warm to -5 ºC over 2h.

Step 2: Double reductive amination

To a solution of NH_4HCO_2 (7.05 g, 111.8 mmol, 20 equiv.) in 280 mL of MeOH, $NaBH_3CN$ (1.40 g, 22.3 mmol, 4 equiv.) and Na_2SO_4 (3.17 g, 22.3 mmol, 4 equiv.) were added. The resulting mixture was cooled to 0 ºC, and the crude Swern oxidation reaction mixture was added under vigorous stirring. The combined mixture was kept stirring at 0 ºC for 1 h, and then at room temperature for 20 h. After this time, the pH was adjusted to *ca.* 10 by adding 1 M NaOH at 0 ºC and then the solvent was evaporated under reduced pressure. 100 mL of water was added, and the aqueous mixture was extracted with EtOAc (3x200 mL). The combined organic extracts were dried ($MgSO_4$), filtered and concentrated under reduced pressure. The crude residue was purified by silica gel flash column chromatography (PE/EtOAc 80:20 → 70:30 → 50:50) providing the title compound **17** (277.0 mg, 48%) as a colorless syrup (1.80 g, 61%). R_f 0.26 (PE/EtOAc 1:1); $[\alpha]_D^{25} + 27$ (*c* 3.7, CH_2Cl_2); The observed NMR data are in agreement with reported data:[1] ^{1}H-NMR δH ($CDCl_3$, 400.13 MHz) 7.38-7.27 (18H, m, Ar-H), 7.23-7.21 (2H, m, Ar-H), 5.01-4.99 (2H, m, 2xCHPh), 4.87 (1H, d, *J* = 10.5 Hz, CHPh), 4.70 (2H, dd, *J* = 15.7 Hz, *J* = 11.6 Hz, CH_2Ph), 4.53-4.43 (3H, m, 3xCHPh), 3.69 (1H, dd, *J* = 9.0 Hz, *J* = 2.6 Hz, CH_a-6), 3.58 (1H, t, *J* = 8.8 Hz, 3-H), 3.55-3.48 (2H, m, CH_6-6, 2-H), 3.37 (1H, t, *J* = 9.2 Hz, 4-H), 3.26 (1H, dd, *J* = 12.3 Hz, *J* = 4.9 Hz, CH_a-1), 2.76-2.72 (1H, m, 5-H), 2.52 (1H, dd, *J* = 12.3 Hz, *J* = 10.2 Hz, CH_b-1), 1.92 (1H, brs, NH); ^{13}C-NMR δC ($CDCl_3$, 100.61 MHz) 139.0 (Ar), 138.6 (Ar), 138.5 (Ar), 138.1 (Ar), 128.5-127.6 (Ar), 87.4 (3-C), 80.8 (2-C), 80.2 (4-C), 75.8 (CH_2Ph), 75.3 (CH_2Ph), 73.5 (CH_2Ph), 72.9 (CH_2Ph), 70.4 (6-C), 59.9 (5-C), 48.2 (1-C).

3. Compounds 18a-d

3.1. *N*-(3-bromopropyl)-2,3,4,6-tetra-*O*-benzyl-1-deoxynojirimycin **(18a)**

The crude product of **18a** was purified by silica gel flash column chromatography (PE/EtOAc 90:10 → 80:20) to provide **18a** (93.2 mg, 44%) as a colorless syrup. R_f 0.58 (PE/EtOAc 7:3); $[\alpha]_D^{24} + 7$ (*c* 0.28, CH_2Cl_2); ^{1}H-NMR δH ($CDCl_3$, 400.13 MHz) 7.34-7.26 (18H, m, ArH), 7.15-7.12 (2H, m, ArH), 4.95 (1H, d, *J* = 11.0 Hz, CHPh), 4.87 (1H, d, *J* = 10.8 Hz, CHPh), 4.81 (1H, d, *J* = 11.0 Hz, CHPh), 4.70 (1H, d, *J* = 11.6 Hz, CHPh), 4.64 (1H, d, *J* = 11.6 Hz, CHPh), 4.48-4.42 (3H, m, 3xCHPh), 3.69-3.50 (3H, m, 4-H, CH_2-6), 3.55 (1H, t, $J_{2,3}$ = 9.2 Hz, 2-H), 3.47 (1H, t, $J_{3,2}$ = 9.0 Hz, 3-H), 3.37-3.29 (2H, m, CH_2Br), 3.04 (1H, dd, $J_{1a,1b}$ = 11.2 Hz, $J_{1a,2}$ = 4.7 Hz, CHa-1), 2.88-2.83 (1H, m, CHaN), 2.71-2.67 (1H, m, CHbN), 2.37-2.32 (1H, m, 5-H), 2.21 (1H, t, $J_{1b,1a}$

= 10.8 Hz, CHb-1), 2.00-1.87 (2H, m, CH_2); ^{13}C-NMR δC ($CDCl_3$, 100.61 MHz) 139.1 (Ar), 138.6 (Ar), 137.9 (Ar), 128.6-127.6 (Ar), 83.4 (3-C), 78.7 (2-C), 78.5 (4-C), 75.5 (CH_2Ph), 75.4 (CH_2Ph), 73.6 (CH_2Ph), 73.0 (CH_2Ph), 66.1 (6-C), 64.2 (5-C), 54.9 (1-C), 50.8 (CH_2N), 32.0 (CH_2Br), 27.9 (CH_2). HRMS (ESI); calcd for $C_{37}H_{43}NO_4Br^+$ 644.2370; found 644.2363.

3.2. *N*-(6-bromohexyl)-2,3,4,6-tetra-*O*-benzyl-1-deoxynojirimycin **(18b)**

The crude product of **18b** was purified by silica gel flash column chromatography (PE/EtOAc 90:10 → 80:20) to provide **18b** (151.2 mg, 61%) as a colorless syrup. R_f 0.59 (PE/EtOAc 7:3); $[\alpha]_D^{24}$ + 6 (*c* 0.33, CH_2Cl_2); ^{1}H-NMR δH ($CDCl_3$, 400.13 MHz) 7.35-7.25 (18H, m, ArH), 7.15-7.13 (2H, m, ArH), 4.96 (1H, d, *J* = 11.1 Hz, CHPh), 4.88 (1H, d, *J* = 10.8 Hz, CHPh), 4.81 (1H, d, *J* = 11.1 Hz, CHPh), 4.70 (1H, d, *J* = 11.6 Hz, CHPh), 4.65 (1H, d, *J* = 11.6 Hz, CHPh), 4.51-4.41 (3H, m, 3xCHPh), 3.68-3.62 (2H, m, 4-H, CHa-6), 3.58 (1H, t, $J_{2,3}$ = 9.3 Hz, 2-H), 3.54 (1H, dd, $J_{6b,6a}$ = 10.4 Hz, $J_{6b,5}$ = 2.0 Hz, CHb-6), 3.46 (1H, t, $J_{3,2}$ = 9.0 Hz, 3-H), 3.38 (2H, t, *J* = 6.8 Hz, CH_2Br), 3.07 (1H, dd, $J_{1a,1b}$ = 11.1 Hz, $J_{1a,2}$ = 4.8 Hz, CHa-1), 2.70-2.63 (1H, m, CHaN), 2.58-2.51 (1H, m, CHbN), 2.31-2.28 (1H, m, 5-H), 2.21 (1H, t, $J_{1b,1a}$ = 10.8 Hz, CHb-1), 1.81 (2H, quint, *J* = 7.1 Hz, CH_2), 1.43-1.29 (4H, m, 2xCH_2), 1.23-1.13 (2H, m, CH_2); ^{13}C-NMR δC ($CDCl_3$, 100.61 MHz) 139.2 (Ar), 138.7 (2xAr), 137.9 (Ar), 128.6-127.6 (Ar), 87.5 (3-C), 78.7 (2-C), 78.6 (4-C), 75.4 (CH_2Ph), 75.3 (CH_2Ph), 73.6 (CH_2Ph), 72.9 (CH_2Ph), 65.5 (6-C), 63.9 (5-C), 54.6 (1-C), 52.3 (CH_2N), 33.9 (CH_2Br), 32.8 (CH_2), 28.1 (CH_2), 26.7 (CH_2), 23.7 (CH_2). HRMS (ESI); calcd for $C_{40}H_{49}NO_4Br^+$ 686.2839; found 686.2828.

3.3. *N*-(8-bromooctyl)-2,3,4,6-tetra-*O*-benzyl-1-deoxynojirimycin **(18c)**

The crude product of **18c** was purified by silica gel flash column chromatography (PE/EtOAc 90:10 → 80:20) to provide **18c** (172.2 mg, 77%) as a white solid. R_f 0.65 (PE/EtOAc 7:3); $[\alpha]_D^{24}$ + 6 (*c* 1.0, CH_2Cl_2); ^{1}H-NMR δH ($CDCl_3$, 400.13 MHz) 7.36-7.24 (18H, m, ArH), 7.15-7.13 (2H, m, ArH), 4.96 (1H, d, *J* = 11.1 Hz, CHPh), 4.88 (1H, d, *J* = 10.8 Hz, CHPh), 4.82 (1H, d, *J* = 11.1 Hz, CHPh), 4.70 (1H, d, *J* = 11.6 Hz, CHPh), 4.65 (1H, d, *J* = 11.6 Hz, CHPh), 4.51-4.41 (3H, m, 3xCHPh), 3.70-3.63 (2H, m, 4-H, CHa-6), 3.60 (1H, t, $J_{2,3}$ = 9.0 Hz, 2-H), 3.54 (1H, dd, $J_{6b,6a}$ = 10.4 Hz, $J_{6b,5}$ = 2.2 Hz, CHb-6), 3.46 (1H, t, $J_{3,2}$ = 9.1 Hz, 3-H), 3.41 (2H, t, *J* = 6.8 Hz, CH_2Br),

3.09 (1H, dd, $J_{1a,1b}$ = 11.1 Hz, $J_{1a,2}$ = 4.9 Hz, CHa-1), 2.70-2.63 (1H, m, CHaN), 2.60-2.52 (1H, m, CHbN), 2.30 (1H, dt, $J_{5,4}$ = 10.0 Hz, $J_{5,6}$ = 2.5 Hz, 5-H), 2.23 (1H, t, $J_{1b,1a}$ = 10.8 Hz, CHb-1), 1.85 (2H, quint, J = 7.1 Hz, CH_2), 1.45-1.22 (8H, m, 4xCH_2), 1.21-1.10 (2H, m, CH_2); ^{13}C-NMR δC ($CDCl_3$, 100.61 MHz) 139.2 (Ar), 138.7 (2xAr), 137.9 (Ar), 128.6-127.5 (Ar), 87.5 (3-C), 78.8 (2-C), 78.7 (4-C), 75.4 (CH_2Ph), 75.3 (CH_2Ph), 73.6 (CH_2Ph), 72.9 (CH_2Ph), 65.4 (6-C), 63.8 (5-C), 54.6 (1-C), 52.5 (CH_2N), 34.1 (CH_2Br), 32.9 (CH_2), 29.4 (CH_2), 28.8 (CH_2), 28.2 (CH_2), 27.5 (CH_2), 23.7 (CH_2). HRMS (ESI); calcd for $C_{42}H_{53}NO_4Br^+$ 714.3152; found 714.3151.

3.4.*N*-(10-bromodecyl)-2,3,4,6-tetra-*O*-benzyl-1-deoxynojirimycin **(18d)**

The crude product of **18d** was purified by silica gel flash column chromatography (PE/EtOAc 90:10 → 80:20) to provide **18d** (171.7 mg, 64%) as a colorless syrup. R_f 0.67 (PE/EtOAc 7:3); $[\alpha]_D^{25}$ + 5 (*c* 0.39, CH_2Cl_2); ^{1}H-NMR δH ($CDCl_3$, 400.13 MHz) 7.35-7.25 (18H, m, ArH), 7.14-7.12 (2H, m, ArH), 4.95 (1H, d, J = 11.1 Hz, CHPh), 4.87 (1H, d, J = 10.8 Hz, CHPh), 4.81 (1H, d, J = 11.1 Hz, CHPh), 4.69 (1H, d, J = 11.6 Hz, CHPh), 4.65 (1H, d, J = 11.6 Hz, CHPh), 4.51-4.40 (3H, m, 3xCHPh), 3.69-3.62 (2H, m, 4-H, CHa-6), 3.59 (1H, t, $J_{2,3}$ = 9.3 Hz, 2-H), 3.54 (1H, dd, $J_{6b,6a}$ = 10.4 Hz, $J_{6b,5}$ = 2.2 Hz, CHb-6), 3.46 (1H, t, $J_{3,2}$ = 9.1 Hz, 3-H), 3.41 (2H, t, J = 6.8 Hz, CH_2Br), 3.09 (1H, dd, $J_{1a,1b}$ = 11.1 Hz, $J_{1a,2}$ = 4.9 Hz, CHa-1), 2.70-2.62 (1H, m, CHaN), 2.59-2.52 (1H, m, CHbN), 2.31-2.28 (1H, m, 5-H), 2.22 (1H, t, $J_{1b,1a}$ = 10.8 Hz, CHb-1), 1.86 (2H, t, J = 7.1 Hz, CH_2), 1.44-1.39 (4H, m, 2xCH_2), 1.33-1.25 (10H, m, 5xCH_2); ^{13}C-NMR δC ($CDCl_3$, 100.61 MHz) 139.2 (Ar), 138.7 (2xAr), 138.0 (Ar), 128.6-127.6 (Ar), 87.5 (3-C), 78.8 (2-C), 78.7 (4-C), 75.4 (CH_2Ph), 75.3 (CH_2Ph), 73.6 (CH_2Ph), 72.9 (CH_2Ph), 65.5 (6-C), 63.9 (5-C), 54.6 (1-C), 52.6 (CH_2N), 34.2 (CH_2Br), 29.8 (CH_2), 29.6 (2xCH_2), 29.5 (CH_2), 28.9 (CH_2), 28.3 (CH_2), 27.6 (CH_2), 23.7 (CH_2). HRMS (ESI); calcd for $C_{44}H_{57}NO_4Br^+$ 742.3465; found 742.3462.

4. Compounds 19a-d

4.1.*N*-(3-azidopropyl)-2,3,4,6-tetra-*O*-benzyl-1-deoxynojirimycin **(19a)**

The reaction provided **19a** (127.3 mg, 95%) as a yellow syrup. R_f 0.59 (PE/EtOAc 7:3); $[\alpha]_D^{25}$ + 10 (*c* 0.19, CH_2Cl_2); ^{1}H-NMR δH ($CDCl_3$, 400.13 MHz) 7.34-7.26 (18H, m, ArH), 7.16-

7.13 (2H, m, ArH), 4.96 (1H, d, J = 11.0 Hz, CHPh), 4.88 (1H, d, J = 10.8 Hz, CHPh), 4.82 (1H, d, J = 11.0 Hz, CHPh), 4.70 (1H, d, J = 11.6 Hz, CHPh), 4.64 (1H, d, J = 11.6 Hz, CHPh), 4.50-4.42 (3H, m, 3xCHPh), 3.67-3.53 (4H, m, 4-H, CH_2N_3, CHa-6), 3.47 (1H, t, $J_{2,3}$ = 8.9 Hz, 2-H), 3.25-3.14 (2H, m, 3-H, CHb-6), 3.04 (1H, dd, $J_{1a,1b}$ = 11.2 Hz, $J_{1a,2}$ = 4.9 Hz, CHa-1), 2.83-2.75 (1H, m, CHaN), 2.63-2.56 (1H, m, CHbN), 2.32 (1H, dt, $J_{5,4}$ = 9.3 Hz, $J_{5,6}$ = 2.3 Hz, 5-H), 2.20 (1H, t, $J_{1b,1a}$ = 10.8 Hz, CHb-1), 1.72-1.60 (2H, m, CH_2); ^{13}C-NMR δC ($CDCl_3$, 100.61 MHz) 139.1 (Ar), 138.6 (Ar), 137.9 (Ar), 128.5-127.6 (Ar), 83.4 (3-C), 78.7 (2-C), 78.5 (4-C), 75.5 (CH_2Ph), 75.4 (CH_2Ph), 73.5 (CH_2Ph), 73.0 (CH_2Ph), 66.0 (6-C), 64.2 (5-C), 54.7 (1-C), 49.8 (CH_2N), 49.5 (CH_2N_3), 24.1 (CH_2). HRMS (ESI); calcd for $C_{37}H_{43}N_4O_4^+$ 607.3279; found 607.3279.

4.2.*N*-(6-azidohexyl)-2,3,4,6-tetra-*O*-benzyl-1-deoxynojirimycin **(19b)**

The reaction provided **19b** (104.7 mg, 97%) as a yellow syrup. R_f 0.59 (PE/EtOAc 7:3); $[\alpha]_D^{25}$ + 4 (c 4.8, CH_2Cl_2); ^{1}H-NMR δH ($CDCl_3$, 400.13 MHz) 7.35-7.27 (18H, m, ArH), 7.15-7.13 (2H, m, ArH), 4.95 (1H, d, J = 11.1 Hz, CHPh), 4.88 (1H, d, J = 10.8 Hz, CHPh), 4.81 (1H, d, J = 11.1 Hz, CHPh), 4.69 (1H, d, J = 11.6 Hz, CHPh), 4.65 (1H, d, J = 11.6 Hz, CHPh), 4.51-4.41 (3H, m, 3xCHPh), 3.68-3.52 (4H, m, 3-H, 4-H, CH_2-6), 3.46 (1H, t, $J_{2,3}$ = 9.0 Hz, 2-H), 3.23 (2H, t, J = 6.9 Hz, CH_2N_3), 3.07 (1H, dd, $J_{1a,1b}$ = 11.1 Hz, $J_{1a,2}$ = 4.9 Hz, CHa-1), 2.70-2.63 (1H, m, CHaN), 2.58-2.51 (1H, m, CHbN), 2.30 (1H, dt, $J_{5,4}$ = 9.5 Hz, $J_{5,6}$ = 2.2 Hz, 5-H), 2.21 (1H, t, $J_{1b,1a}$ = 10.8 Hz, CHb-1), 1.44-1.12 (8H, m, 4xCH_2); ^{13}C-NMR δC ($CDCl_3$, 100.61 MHz) 139.2 (Ar), 138.7 (2xAr), 137.9 (Ar), 128.6-127.6 (Ar), 87.5 (3-C), 78.8 (2-C), 78.7 (4-C), 75.4 (CH_2Ph), 75.3 (CH_2Ph), 73.6 (CH_2Ph), 72.9 (CH_2Ph), 65.6 (6-C), 63.9 (5-C), 54.6 (1-C), 52.4 (CH_2N), 51.5 (CH_2N_3), 28.9 (CH_2), 27.2 (CH_2), 26.7 (CH_2), 23.7 (CH_2). HRMS (ESI); calcd for $C_{40}H_{49}N_4O_4^+$ 649.3748; found 649.3736.

4.3.*N*-(8-azidooctyl)-2,3,4,6-tetra-*O*-benzyl-1-deoxynojirimycin **(19c)**

The reaction provided **19c** (134.1 mg, 95%) as a yellow syrup. R_f 0.52 (PE/EtOAc 7:3); $[\alpha]_D^{25}$ + 3 (c 0.65, CH_2Cl_2); ^{1}H-NMR δH ($CDCl_3$, 400.13 MHz) 7.35-7.24 (18H, m, ArH), 7.15-7.13 (2H, m, ArH), 4.96 (1H, d, J = 11.1 Hz, CHPh), 4.88 (1H, d, J = 10.8 Hz, CHPh), 4.82 (1H, d, J = 11.1

Hz, CHPh), 4.69 (1H, d, *J* = 11.6 Hz, CHPh), 4.65 (1H, d, *J* = 11.6 Hz, CHPh), 4.51-4.41 (3H, m, 3xCHPh), 3.69-3.63 (2H, m, 4-H, CHa-6), 3.59 (1H, t, $J_{2,3}$ = 9.0 Hz, 2-H), 3.54 (1H, dd, $J_{6b,6a}$ = 10.4 Hz, $J_{6b,5}$ = 2.2 Hz, CHb-6), 3.46 (1H, t, $J_{3,2}$ = 9.1 Hz, 3-H), 3.26 (2H, t, *J* = 6.9 Hz, CH_2N_3), 3.09 (1H, dd, $J_{1a,1b}$ = 11.1 Hz, $J_{1a,2}$ = 4.9 Hz, CHa-1), 2.70-2.63 (1H, m, CHaN), 2.59-2.52 (1H, m, CHbN), 2.31-2.29 (1H, m, 5-H), 2.22 (1H, t, $J_{1b,1a}$ = 10.8 Hz, CHb-1), 1.63-1.56 (2H, m, CH_2), 1.43-1.22 (8H, m, 4xCH_2), 1.20-1.10 (2H, m, CH_2); ^{13}C-NMR δC ($CDCl_3$, 100.61 MHz) 139.2 (Ar), 138.7 (2xAr), 138.0 (Ar), 128.6-127.6 (Ar), 87.5 (3-C), 78.8 (2-C), 78.7 (4-C), 75.4 (CH_2Ph), 75.3 (CH_2Ph), 73.6 (CH_2Ph), 72.9 (CH_2Ph), 65.5 (6-C), 63.9 (5-C), 54.6 (1-C), 52.5 (CH_2N), 51.6 (CH_2N_3), 29.5 (CH_2), 29.3 (CH_2), 29.0 (CH_2), 27.5 (CH_2), 26.8 (CH_2), 23.7 (CH_2). HRMS (ESI); calcd for $C_{42}H_{53}N_4O_4^+$ 677.4061; found 677.4050.

4.4.*N*-(10-azidodecyl)-2,3,4,6-tetra-*O*-benzyl-1-deoxynojirimycin **(19d)**

The reaction provided **19d** (153.3 mg, 98%) as a yellow syrup. R_f 0.28 (PE/EtOAc 8:2); $[\alpha]_D^{26} + 4$ (*c* 0.49, CH_2Cl_2); ^{1}H-NMR δH ($CDCl_3$, 400.13 MHz) 7.35-7.26 (18H, m, ArH), 7.14-7.12 (2H, m, ArH), 4.95 (1H, d, *J* = 11.1 Hz, CHPh), 4.87 (1H, d, *J* = 10.8 Hz, CHPh), 4.81 (1H, d, *J* = 11.1 Hz, CHPh), 4.69 (1H, d, *J* = 11.6 Hz, CHPh), 4.65 (1H, d, *J* = 11.6 Hz, CHPh), 4.49 (1H, d, *J* = 12.0 Hz, CHPh), 4.46 (1H, d, *J* = 12.3 Hz, CHPh), 4.42 (1H, d, *J* = 10.9 Hz, CHPh), 3.69-3.62 (2H, m, 4-H, CHa-6), 3.59 (1H, t, $J_{2,3}$ = 9.3 Hz, 2-H), 3.54 (1H, dd, $J_{6b,6a}$ = 10.4 Hz, $J_{6b,5}$ = 2.2 Hz, CHb-6), 3.45 (1H, t, $J_{3,2}$ = 9.1 Hz, 3-H), 3.26 (2H, t, *J* = 7.0 Hz, CH_2N_3), 3.09 (1H, dd, $J_{1a,1b}$ = 11.1 Hz, $J_{1a,2}$ = 4.8 Hz, CHa-1), 2.70-2.63 (1H, m, CHaN), 2.60-2.52 (1H, m, CHbN), 2.30 (1H, dt, $J_{5,4}$ = 9.3 Hz, $J_{5,6}$ = 2.3 Hz, 5-H), 2.23 (1H, t, $J_{1b,1a}$ = 10.8 Hz, CHb-1), 1.64-1.58 (2H, m, CH_2), 1.43-1.22 (12H, m, 6xCH_2), 1.18-1.13 (2H, m, CH_2); ^{13}C-NMR δC ($CDCl_3$, 100.61 MHz) 139.2 (Ar), 138.7 (2xAr), 138.0 (Ar), 128.6-127.5 (Ar), 87.5 (3-C), 78.8 (2-C), 78.7 (4-C), 75.4 (CH_2Ph), 75.3 (CH_2Ph), 73.6 (CH_2Ph), 72.9 (CH_2Ph), 65.4 (6-C), 63.8 (5-C), 54.6 (1-C), 52.5 (CH_2N), 51.6 (CH_2N_3), 29.6 (2xCH_2), 29.5 (CH_2), 29.3 (CH_2), 29.0 (CH_2), 27.6 (CH_2), 26.9 (CH_2), 23.7 (CH_2). HRMS (ESI); calcd for $C_{44}H_{57}N_4O_4^+$ 705.4374; found 705.4362.

5. 1-(2-Propyn-1-yl)-1*H*-benzotriazole (20)

1-*H*-benzotriazole (439.6 mg, 3.69 mmol, 1 equiv.) was suspended in dry DMF (10 mL) and potassium carbonate (765.1 mg, 5.53 mmol, 1.5 equiv.) was added. The mixture was allowed to

stir for 10 minutes before propargyl bromide 80% solution in toluene (0.62 mL, 5.53 mmol, 1.5 equiv) was added dropwise. The reaction was let stirring at room temperature for 24 h. After this time, the solvent was removed under reduced pressure and the crude was suspended in ethyl acetate (50 mL) and washed with water (2x20mL). The organic layers were combined, dried ($MgSO_4$), filtered, and concentrated under reduced pressure. Purification of the residue by silica gel flash column chromatography (PE/EtOAc 90:10 → 85:15) provided the title compound **20** (277.0 mg, 48%) as white crystals. R_f 0.39 (PE/EtOAc 7:3); mp: 56-58 °C {Lit.[2] mp:57-58 °C}; The observed NMR data are in agreement with reported data:[3] ^{1}H-NMR δH ($CDCl_3$, 400.13 MHz) 8.05 (1H, dt, *J* = 8.4 Hz, *J* = 0.7 Hz, Ar-H), 7.69 (1H, dt, *J* = 8.3 Hz, *J* = 0.8 Hz, Ar-H), 7.53-7.49 (1H, m, Ar-H), 7.40-7.36 (1H, m, Ar-H), 5.45-5.13 (2H, m, CH_2), 2.49 (1H, t, *J* = 0.8 Hz, C≡C-H); ^{13}C-NMR δC ($CDCl_3$, 100.61 MHz) 146.3 (Ar), 132.5 (Ar), 127.8 (Ar), 124.3 (Ar), 120.2 (Ar), 109.9 (Ar), 75.3 (C≡C), 75.2 (C≡C-H), 38.1 (CH_2).

6. Compounds 21a-d

6.1.1. *N*-(1-(2-((1H-benzo[1,2,3]triazol-1-yl)methyl)-1*H*-1,2,3-triazol-1-yl)propyl)-2,3,4,6-tetra-*O*-benzy-1-deoxynojirimycin **(21a)**

The crude product of **21a** was purified by silica gel flash column chromatography (PE/EtOAc 80:20 → 50:50 → 30:70) to provide **21a** (108.5 mg, 71%) as a colorless syrup. R_f 0.33 (PE/EtOAc 3:7); $[\alpha]_D^{26}$ + 6.6 (*c* 0.61, CH_2Cl_2); ^{1}H-NMR δH ($CDCl_3$, 400.13 MHz) 8.04 (1H, dt, *J* = 8.4 Hz, *J* = 0.9 Hz, Ar-H), 7.70 (1H, dt, *J* = 8.3 Hz, *J* = 0.9 Hz, Ar-H), 7.48-7.44 (1H, m, Ar-H), 7.37-7.35 (1H, m, Ar-H), 7.33 (1H, s, H-triazole), 7.32-7.29 (18H, m, Ar-H), 7.16-7.14 (2H, m, Ar-H), 5.90 (2H, s, CH_2N-benzotriazole), 4.94 (1H, d, *J* = 11.1 Hz, CHPh), 4.87 (1H, d, *J* = 10.9 Hz, CHPh), 4.80 (1H, d, *J* = 11.0 Hz, CHPh), 4.67 (1H, d, *J* = 11.6 Hz, CHPh), 4.43 (1H, d, *J* = 10.9 Hz, CHPh), 4.32 (2H, dd, *J* = 13.8 Hz, *J* = 11.9 Hz, CH_2Ph), 4.22-4.15 (1H, m, CHaN-triazole), 4.15-4.05 (1H, m, CHbN-triazole), 3.59 (1H, dd, $J_{6a,6b}$ = 10.6 Hz, $J_{6a,5}$ = 3.3 Hz, CHa-6), 3.56-3.49 (2H, m, CHb-6, 4-H), 3.47-3.45 (2H, m, 2-H, 3-H), 2.95 (1H, dd, $J_{1a,1b}$ = 11.3 Hz, $J_{1a,2}$ = 4.7 Hz, CHa-1), 2.76-2.70 (1H, m, CHaN), 2.48-2.42 (1H, m, CHbN), 2.31-2.29 (1H, m, 5-H), 2.12-2.05 (1H, m, CHb-1), 1.98-1.86 (2H, m, CH_2); ^{13}C-NMR δC ($CDCl_3$, 100.61 MHz) 146.3 (Ar), 142.0 (Ar), 139.0-137.8 (Ar), 132.8 (Ar), 128.6-127.6 (Ar), 124.3 (Ar), 123.1 (CH-triazole), 120.0 (Ar), 110.2 (Ar), 87.1 (3-C), 78.6 (2-C), 78.4 (4-C), 75.5 (CH_2Ph), 75.3 (2xCH_2Ph), 73.0 (CH_2Ph), 66.6 (6-C), 64.6

(5-C), 54.4 (1-C), 49.1 (CH_2N), 48.7 (CH_2N-triazole), 43.9 (CH_2N-benzotriazole), 26.1 (CH_2). HRMS (ESI); calcd for $C_{46}H_{50}N_7O_4^+$ 764.3919; found 764.3904.

6.1.2. *N*-(1-(2-((1H-benzo[1,2,3]triazol-1-yl)methyl)-1*H*-1,2,3-triazol-1-yl)hexyl)-2,3,4,6-tetra-*O*-benzy-1-deoxynojirimycin **(21b)**

The crude product of **21b** was purified by silica gel flash column chromatography (PE/EtOAc 80:20 → 50:50 → 30:70) to provide **21b** (125.9 mg, 80%) as a colorless syrup. R_f 0.26 (PE/EtOAc 3:7); $[\alpha]_D^{26}$ + 3.1 (*c* 0.65, CH_2Cl_2); ^{1}H-NMR δH ($CDCl_3$, 400.13 MHz) 8.04 (1H, dt, J = 8.4 Hz, J = 0.9 Hz, Ar-H), 7.72 (1H, dt, J = 8.3 Hz, J = 0.9 Hz, Ar-H), 7.50-7.47 (1H, m, Ar-H), 7.45 (1H, s, H-triazole), 7.38-7.34 (1H, m, Ar-H), 7.33-7.22 (18H, m, Ar-H), 7.15-7.13 (2H, m, Ar-H), 5.97 (2H, s, CH_2N-benzotriazole), 4.95 (1H, d, J = 11.1 Hz, CHPh), 4.87 (1H, d, J = 10.9 Hz, CHPh), 4.81 (1H, d, J = 11.1 Hz, CHPh), 4.68 (1H, d, J = 11.6 Hz, CHPh), 4.64 (1H, d, J = 11.6 Hz, CHPh), 4.48-4.40 (3H, m, 3xCHPh), 4.23 (2H, t, J = 7.3 Hz, CH_2N-triazole), 3.66-3.60 (2H, m, CHa-6, 4-H), 3.56 (1H, t, $J_{2,3}$ = 9.2 Hz, 2-H), 3.51 (1H, dd, $J_{6b,6a}$ = 10.4 Hz, $J_{6b,5}$ = 2.0 Hz, CHb-6), 3.45 (1H, t, $J_{3,2}$ = 9.2 Hz, 3-H), 3.04 (1H, dd, $J_{1a,1b}$ = 11.1 Hz, $J_{1a,2}$ = 4.8 Hz, CHa-1), 2.67-2.59 (1H, m, CHaN), 2.53-2.46 (1H, m, CHbN), 2.27 (1H, dt, $J_{5,4}$ = 9.7 Hz, $J_{5,6b}$ = 2.0 Hz, 5-H), 2.17 (1H, t, $J_{1b,1a}$ = 10.8 Hz, CHb-1), 1.79 (2H, quint, J = 7.3 Hz, CH_2), 1.38-1.10 (6H, m, 3xCH_2); ^{13}C-NMR δC ($CDCl_3$, 100.61 MHz) 146.3 (Ar), 142.2 (Ar), 139.1 (Ar), 138.7 (Ar), 137.9 (Ar), 132.8 (Ar), 128.5-127.6 (Ar), 124.3 (Ar), 122.7 (CH-triazole), 120.0 (Ar), 110.2 (Ar), 87.5 (3-C), 78.7 (2-C), 78.6 (4-C), 75.4 (CH_2Ph), 75.3 (CH_2Ph), 73.5 (CH_2Ph), 72.9 (CH_2Ph), 65.7 (6-C), 63.9 (5-C), 54.6 (1-C), 52.2 (CH_2N), 50.5 (CH_2N-triazole), 44.0 (CH_2N-benzotriazole), 30.2 (CH_2), 26.9 (CH_2), 26.4 (CH_2), 23.7 (CH_2). HRMS (ESI); calcd for $C_{49}H_{56}N_7O_4^+$ 806.4388; found 806.4385.

6.1.3. *N*-(1-(2-((1*H*-benzo[1,2,3]triazol-1-yl)methyl)-1*H*-1,2,3-triazol-1-yl)octyl)-2,3,4,6-tetra-*O*-benzy-1-deoxynojirimycin **(21c)**

The crude product of **21c** was purified by silica gel flash column chromatography (PE/EtOAc 80:20 → 50:50 → 30:70) to provide **21c** (125.8 mg, 84%) as a colorless syrup. R_f 0.29 (PE/EtOAc 1:1); $[\alpha]_D^{26}$ + 6.4 (*c* 0.31, CH_2Cl_2); ^{1}H-NMR δH ($CDCl_3$, 400.13 MHz) 8.04 (1H, dt, J = 8.4 Hz, J = 0.9 Hz, Ar-H), 7.72 (1H, dt, J = 8.4 Hz, J = 0.9 Hz, Ar-H), 7.50-7.45 (2H, m, Ar-H, H-triazole),

7.38-7.34 (1H, m, Ar-H), 7.33-7.22 (18H, m, Ar-H), 7.14-7.12 (2H, m, Ar-H), 5.97 (2H, s, CH_2N-benzotriazole), 4.95 (1H, d, J = 11.1 Hz, CHPh), 4.87 (1H, d, J = 10.1 Hz, CHPh), 4.81 (1H, d, J = 11.1 Hz, CHPh), 4.69 (1H, d, J = 11.6 Hz, CHPh), 4.64 (1H, d, J = 11.6 Hz, CHPh), 4.50-4.46 (2H, m, 2xCHPh), 4.41 (1H, d, J = 10.9 Hz, CHPh), 4.27 (2H, t, J = 7.3 Hz, CH_2N-triazole), 3.68-3.62 (2H, m, CHa-6, 4-H), 3.58 (1H, t, $J_{2,3}$ = 9.3 Hz, 2-H), 3.53 (1H, dd, $J_{6b,6a}$ = 10.4 Hz, $J_{6b,5}$ = 2.1 Hz, CHb-6), 3.45 (1H, t, $J_{3,2}$ = 9.0 Hz, 3-H), 3.07 (1H, dd, $J_{1a,1b}$ = 11.1 Hz, $J_{1a,2}$ = 4.8 Hz, CHa-1), 2.68-2.61 (1H, m, CHaN), 2.57-2.50 (1H, m, CHbN), 2.29 (1H, dt, $J_{5,4}$ = 9.5 Hz, $J_{5,6b}$ = 2.3 Hz, 5-H), 2.21 (1H, t, $J_{1b,1a}$ = 10.8 Hz, CHb-1), 1.83 (2H, quint, J = 7.1 Hz, CH_2), 1.37-1.05 (10H, m, 5xCH_2); ^{13}C-NMR δC ($CDCl_3$, 100.61 MHz) 146.3 (Ar), 142.2 (Ar), 139.1 (Ar), 138.7 (Ar), 138.0 (Ar), 132.8 (Ar), 128.5-127.5 (Ar), 124.3 (Ar), 122.7 (CH-triazole), 120.0 (Ar), 110.2 (Ar), 87.5 (3-C), 78.7 (2-C), 78.6 (4-C), 75.4 (CH_2Ph), 75.3 (CH_2Ph), 73.5 (CH_2Ph), 72.9 (CH_2Ph), 65.5 (6-C), 63.8 (5-C), 54.6 (1-C), 52.4 (CH_2N), 50.7 (CH_2N-triazole), 44.0 (CH_2N-benzotriazole), 30.2 (CH_2), 29.4 (CH_2), 29.0 (CH_2), 27.5 (CH_2), 26.5 (CH_2), 23.7 (CH_2). HRMS (ESI); calcd for $C_{51}H_{60}N_7O_4^+$ 834.4701; found 834.4692.

6.1.4. *N*-(1-(2-((1H-benzo[1,2,3]triazol-1-yl)methyl)-1*H*-1,2,3-triazol-1-yl)decyl)-2,3,4,6-tetra-*O*-benzy-1-deoxynojirimycin **(21d)**

The crude product of **21d** was purified by silica gel flash column chromatography (PE/EtOAc 80:20 → 50:50 → 30:70) to provide **21d** (134.7 mg, 80%) as a colorless syrup. R_f 0.33 (PE/EtOAc 1:1); $[\alpha]_D^{26}$ + 5.0 (*c* 1.2, CH_2Cl_2); ^{1}H-NMR δH ($CDCl_3$, 400.13 MHz) 8.04 (1H, dt, J = 8.4 Hz, J = 0.9 Hz, Ar-H), 7.72 (1H, dt, J = 8.3 Hz, J = 0.9 Hz, Ar-H), 7.49-7.45 (2H, m, Ar-H, H-triazole), 7.38-7.36 (1H, m, Ar-H), 7.34-7.24 (18H, m, Ar-H), 7.14-7.12 (2H, m, Ar-H), 5.97 (2H, s, CH_2N-benzotriazole), 4.95 (1H, d, J = 11.1 Hz, CHPh), 4.87 (1H, d, J = 10.8 Hz, CHPh), 4.81 (1H, d, J = 11.1 Hz, CHPh), 4.69 (1H, d, J = 11.6 Hz, CHPh), 4.65 (1H, d, J = 11.6 Hz, CHPh), 4.50-4.44 (2H, m, 2xCHPh), 4.41 (1H, d, J = 10.9 Hz, CHPh), 4.28 (2H, t, J = 7.3 Hz, CH_2N-triazole), 3.68-3.62 (2H, m, CHa-6, 4-H), 3.59 (1H, t, $J_{2,3}$ = 9.3 Hz, 2-H), 3.534 (1H, dd, $J_{6b,6a}$ = 10.4 Hz, $J_{6b,5}$ = 2.1 Hz, CHb-6), 3.45 (1H, t, $J_{3,2}$ = 8.9 Hz, 3-H), 3.09 (1H, dd, $J_{1a,1b}$ = 11.1 Hz, $J_{1a,2}$ = 4.8 Hz, CHa-1), 2.67-2.62 (1H, m, CHaN), 2.59-2.53 (1H, m, CHbN), 2.31-2.28 (1H, m, 5-H), 2.22 (1H, t, $J_{1b,1a}$ = 10.8 Hz, CHb-1), 1.88-1.82 (2H, m, CH_2), 1.39-1.11 (14H, m, 7xCH_2); ^{13}C-NMR δC ($CDCl_3$, 100.61 MHz) 146.3 (Ar), 142.2 (Ar), 139.2 (Ar), 138.7 (Ar), 138.0 (Ar), 132.8 (Ar), 128.5-127.5

(Ar), 124.3 (Ar), 122.7 (CH-triazole), 120.0 (Ar), 110.3 (Ar), 87.5 (3-C), 78.8 (2-C), 78.7 (4-C), 75.4 (CH_2Ph), 75.3 (CH_2Ph), 73.6 (CH_2Ph), 72.9 (CH_2Ph), 65.5 (6-C), 63.8 (5-C), 54.6 (1-C), 52.5 (CH_2N), 50.7 (CH_2N-triazole), 44.0 (CH_2N-benzotriazole), 30.3 (CH_2), 29.6 (2xCH_2), 29.4 (CH_2), 29.0 (CH_2), 27.6 (CH_2), 26.5 (CH_2), 23.7 (CH_2). HRMS (ESI); calcd for $C_{53}H_{64}N_7O_4^+$ 862.5014; found 862.4998.

7. Compounds 12a-d

7.1. *N*-(1-(2-((1*H*-benzo[1,2,3]triazol-1-yl)methyl)-1*H*-1,2,3-triazol-1-yl)propyl)-1-deoxynojirimycin hydrochloride **(12a)**

The crude product of **12a** was purified by silica gel flash column chromatography (CH_3CN/H_2O 95:5 → 90:10) to provide **12a** (33.7 mg, 64%) as white crystals. R_f 0.16 (CH_3CN/H_2O 9:1); $[\alpha]_D^{26} - 28$ (*c* 0.07, MeOH); mp: 97-99 °C; ^{1}H-NMR δH (MeOD, 400.13 MHz) 8.12 (1H, s, H-triazole), 7.99 (1H, dt, *J* = 8.4 Hz, *J* = 0.9 Hz, Ar-H), 7.83 (1H, dt, *J* = 8.4 Hz, *J* = 0.9 Hz, Ar-H), 7.59-7.55 (1H, m, Ar-H), 7.46-7.42 (1H, m, Ar-H), 6.05 (2H, s, CH_2N-benzotriazole), 4.42 (2H, t, *J* = 6.9 Hz, CH_2N-triazole), 3.79 (1H, dd, $J_{6a,6b}$ = 12.1 Hz, $J_{6a,5}$ = 2.5 Hz, CHa-6), 3.75 (1H, dd, $J_{6b,6a}$ = 12.0 Hz, $J_{6b,5}$ = 3.1 Hz, CHb-6), 3.46-3.40 (1H, m, 2-H), 3.34-3.29 (1H, m, 4-H, overlaps with solvente signal), 3.12 (1H, t, $J_{3,2}$ = 9.0 Hz, 3-H), 2.95 (1H, dd, $J_{1a,1b}$ = 11.1 Hz, $J_{1a,2}$ = 4.8 Hz, CHa-1), 2.88-2.81 (1H, m, CHaN), 2.51-2.44 (1H, m, CHbN), 2.11-2.03 (4H, m, 5-H, CHb-1, CH_2); ^{13}C-NMR δC (MeOD, 100.61 MHz) 146.9 (Ar), 143.1 (Ar), 134.1 (Ar), 129.0 (Ar), 125.8 (Ar), 125.4 (CH-triazole), 120.0 (Ar), 111.6 (Ar), 80.5 (3-C), 71.9 (4-C), 70.7 (2-C), 67.9 (5-C), 59.5 (6-C), 57.6 (1-C), 50.1 (CH_2N), 49.6 (CH_2N-triazole), 44.3 (CH_2N-benzotriazole), 26.9 (CH_2). HRMS (ESI); calcd for $C_{18}H_{26}N_7O_4^+$ 404.2041; found 404.2035.

7.2. *N*-(1-(2-((1*H*-benzo[1,2,3]triazol-1-yl)methyl)-1*H*-1,2,3-triazol-1-yl)hexyl)-1-deoxynojirimycin hydrochloride **(12b)**

The crude product of **12b** was purified by silica gel flash column chromatography (CH_3CN/H_2O 95:5 → 90:10) to provide **12b** (26.1 mg, 46%) as white crystals. R_f 0.13 (CH_3CN/H_2O 9:1); $[\alpha]_D^{26} - 27$ (*c* 0.07, MeOH); mp: 106-108 °C; ^{1}H-NMR δH (MeOD, 400.13 MHz) 8.09 (1H, s, H-triazole), 7.99 (1H, dt, *J* = 8.4 Hz, *J* = 0.9 Hz, Ar-H), 7.82 (1H, dt, *J* = 8.4 Hz, *J* = 0.9 Hz, Ar-H), 7.58-7.54 (1H, m, Ar-H), 7.46-7.42 (1H, m, Ar-H), 6.05 (2H, s, CH_2N-

benzotriazole), 4.37 (2H, t, $J = 7.0$ Hz, CH_2N-triazole), 3.87-3.79 (2H, m, CH_2-6), 3.50-3.44 (1H, m, 2-H), 3.35 (1H, t, $J_{4,3} = 8.4$ Hz, 4-H), 3.14 (1H, t, $J_{3,4} = 9.1$ Hz, 3-H), 2.96 (1H, dd, $J_{1a,1b} = 11.0$ Hz, $J_{1a,2} = 4.9$ Hz, CHa-1), 2.79-2.72 (1H, m, CHaN), 2.55-2.48 (1H, m, CHbN), 2.14 (1H, t, $J_{1b,1a} = 11.0$ Hz, CHb-1), 2.09 (1H, dt, $J_{5,4} = 9.8$ Hz, $J_{5,6} = 2.8$ Hz, 5-H), 1.91-1.85 (2H, m, CH_2), 1.45-1.42 (2H, m, CH_2), 1.31-1.28 (4H, m, 2xCH_2); ^{13}C-NMR δC (MeOD, 100.61 MHz) 146.9 (Ar), 143.1 (Ar), 134.1 (Ar), 128.9 (Ar), 125.8 (Ar), 125.2 (CH-triazole), 120.0 (Ar), 111.6 (Ar), 80.6 (3-C), 72.0 (4-C), 70.7 (2-C), 67.4 (5-C), 59.5 (6-C), 57.7 (1-C), 53.5 (CH_2N), 51.4 (CH_2N-triazole), 44.3 (CH_2N-benzotriazole), 31.1(CH_2), 27.8 (CH_2), 27.2 (CH_2), 25.1 (CH_2). HRMS (ESI); calcd for $C_{21}H_{31}N_7O_4Na^+$ 468.2330; found 468.2325.

7.3. *N*-(1-(2-((1*H*-benzo[1,2,3]triazol-1-yl)methyl)-1*H*-1,2,3-triazol-1-yl)octyl)-1-deoxynojirimycin hydrochloride **(12c)**

The crude product of **12c** was purified by silica gel flash column chromatography (CH_3CN/H_2O 95:5 → 90:10) to provide **12c** (27.5 mg, 45%) as white crystals. R_f 0.10 (CH_3CN/H_2O 9:1); $[\alpha]_D^{27} - 22$ (*c* 0.09, MeOH); mp: 75-77 °C; ^{1}H-NMR δH (MeOD, 400.13 MHz) 8.07 (1H, s, H-triazole), 7.98 (1H, dt, $J = 8.4$ Hz, $J = 0.9$ Hz, Ar-H), 7.81 (1H, dt, $J = 8.4$ Hz, $J = 0.9$ Hz, Ar-H), 7.56-7.52 (1H, m, Ar-H), 7.45-7.41 (1H, m, Ar-H), 6.04 (2H, s, CH_2N-benzotriazole), 4.36 (2H, t, $J = 7.0$ Hz, CH_2N-triazole), 3.88-3.81 (2H, m, CH_2-6), 3.51-3.45 (1H, m, 2-H), 3.36 (1H, t, $J_{4,3} = 9.3$ Hz, 4-H), 3.14 (1H, t, $J_{3,4} = 9.1$ Hz, 3-H), 2.98 (1H, dd, $J_{1a,1b} = 11.2$ Hz, $J_{1a,2} = 4.9$ Hz, CHa-1), 2.81-2.73 (1H, m, CHaN), 2.58-2.51 (1H, m, CHbN), 2.18 (1H, t, $J_{1b,1a} = 10.9$ Hz, CHb-1), 2.13 (1H, dt, $J_{5,4} = 9.6$ Hz, $J_{5,6} = 2.6$ Hz, 5-H), 1.85 (2H, quint, $J = 7.1$ Hz, CH_2), 1.45 (2H, quint, $J = 7.5$ Hz, CH_2), 1.30-1.21 (8H, m, 4xCH_2); ^{13}C-NMR δC (MeOD, 100.61 MHz) 146.9 (Ar), 143.1 (Ar), 134.1 (Ar), 128.9 (Ar), 125.8 (Ar), 125.2 (CH-triazole), 120.0 (Ar), 111.6 (Ar), 80.5 (3-C), 71.9 (4-C), 70.6 (2-C), 67.4 (5-C), 59.3 (6-C), 57.6 (1-C), 53.7 (CH_2N), 51.4 (CH_2N-triazole), 44.3 (CH_2N-benzotriazole), 31.1(CH_2), 30.2 (CH_2), 29.8 (CH_2), 28.3 (CH_2), 27.2 (CH_2), 25.1 (CH_2). HRMS (ESI); calcd for $C_{23}H_{35}N_7O_4Na^+$ 496.2643; found 496.2635.

7.4. *N*-(1-(2-((1*H*-benzo[1,2,3]triazol-1-yl)methyl)-1*H*-1,2,3-triazol-1-yl)decyl)-1-deoxynojirimycin hydrochloride **(12d)**

The crude product of **12d** was purified by silica gel flash column chromatography (CH_3CN/H_2O 95:5 → 90:10) to provide **12d** (31.4 mg, 46%) as white crystals. R_f 0.17 (CH_3CN/H_2O 9:1); $[\alpha]_D^{25} - 12$ (*c* 0.16, MeOH); mp: 108-110 °C; ^{1}H-NMR δH (MeOD, 400.13 MHz) 8.08 (1H, s, H-triazole), 7.98 (1H, dt, J = 8.4 Hz, J = 0.9 Hz, Ar-H), 7.81 (1H, dt, J = 8.4 Hz, J = 0.9 Hz, Ar-H), 7.57-7.53 (1H, m, Ar-H), 7.45-7.41 (1H, m, Ar-H), 6.05 (2H, s, CH_2N-benzotriazole), 4.36 (2H, t, J = 7.0 Hz, CH_2N-triazole), 3.92 (1H, dd, $J_{6a,6b}$ = 12.1 Hz, $J_{6a,5}$ = 2.5 Hz, CHa-6), 3.87 (1H, dd, $J_{6a,6b}$ = 12.1 Hz, $J_{6a,5}$ = 2.7 Hz, CHa-6), 3.57-3.51 (1H, m, 2-H), 3.42 (1H, t, $J_{4,3}$ = 9.4 Hz, 4-H), 3.20 (1H, t, $J_{3,4}$ = 9.1 Hz, 3-H), 3.12 (1H, dd, $J_{1a,1b}$ = 11.4 Hz, $J_{1a,2}$ = 4.8 Hz, CHa-1), 2.97-2.90 (1H, m, CHaN), 2.77-2.70 (1H, m, CHbN), 2.43-2.37 (2H, m, CHb-1, 5-H), 1.85 (2H, quint, J = 7.1 Hz, CH_2), 1.55-1.54 (2H, m, CH_2), 1.30-1.24 (12H, m, 6xCH_2); ^{13}C-NMR δC (MeOD, 100.61 MHz) 146.9 (Ar), 143.0 (Ar), 134.1 (Ar), 128.9 (Ar), 125.8 (Ar), 125.2 (CH-triazole), 120.0 (Ar), 111.6 (Ar), 79.8 (3-C), 71.1 (4-C), 69.9 (2-C), 67.4 (5-C), 58.2 (6-C), 56.9 (1-C), 53.9 (CH_2N), 51.4 (CH_2N-triazole), 44.3 (CH_2N-benzotriazole), 31.1(CH_2), 30.3 (3xCH_2), 29.8 (CH_2), 28.2 (CH_2), 27.3 (CH_2), 24.9 (CH_2). HRMS (ESI); calcd for $C_{25}H_{39}N_7O_4Na^+$ 524.2956; found 524.2946.

8. References

1. Wennekes, T.; van den Berg, R.J.B.H.N.; Donker W.; van der Marel, G.A.; Strijland, A.; Aerts, J.M.F:G.; Overkleeft, H.S. *J. Org. Chem.* **2007**, *72*, 1088–1097.
2. Khalafi-Nezhad A.; Zare A.; Parhami A.; Rad M.N.S.; Nejabat GR. *J. Iran. Chem. Soc.* **2007**, *4*, 271-278.
3. Perego LA, Blieck R, Michel J, Ciofini I, Taillefer M, Monnier F. *Adv. Synth. Catal.* **2017**, *359*, 4388-4392.

9. NMR Spectra

^{1}H-NMR spectra of compound **16** ($CDCl_3$, 400.13 MHz)

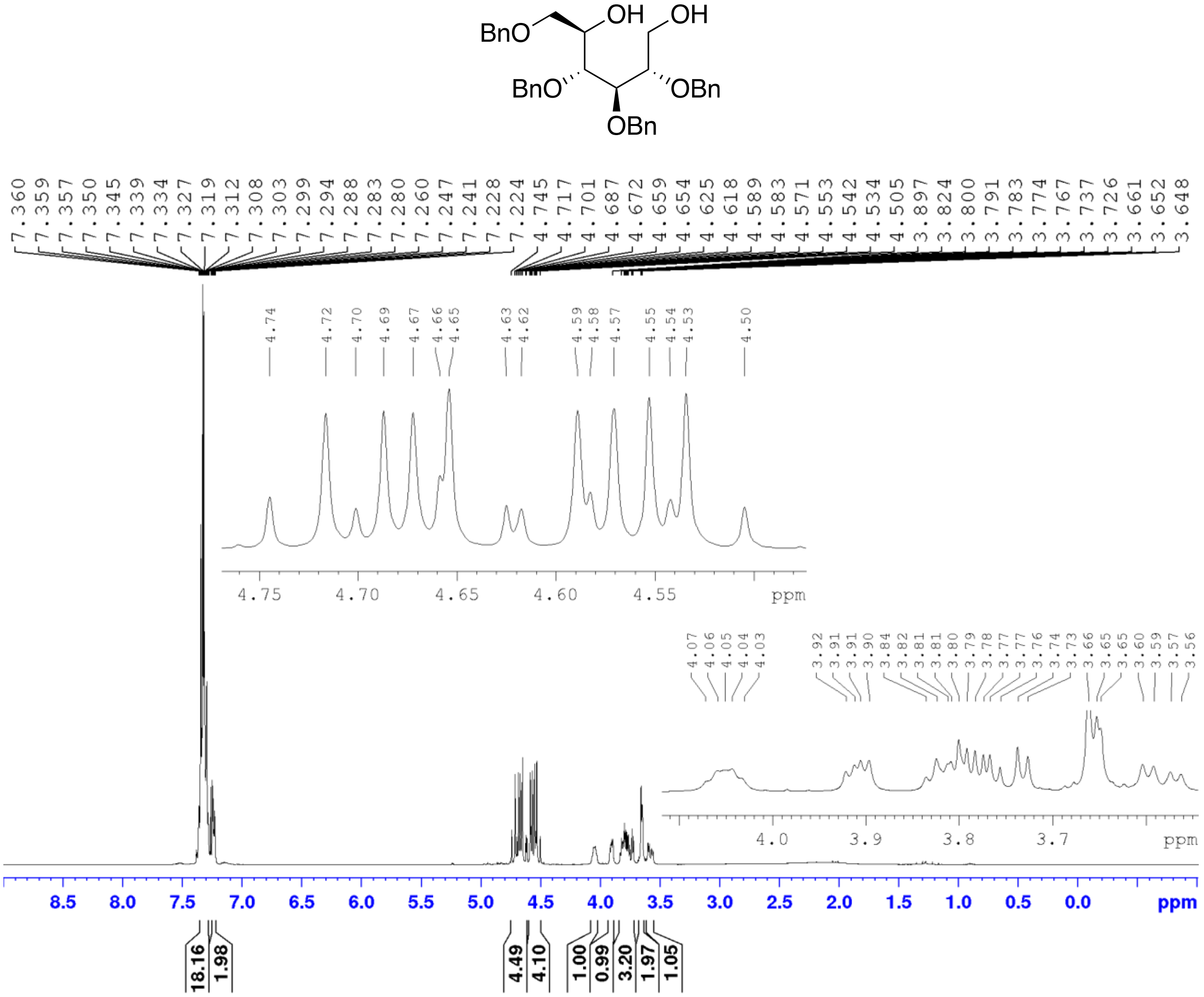

^{13}C-NMR spectra of compound **16** ($CDCl_3$, 100.61 MHz)

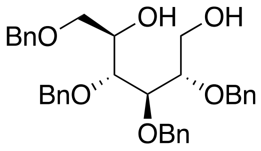

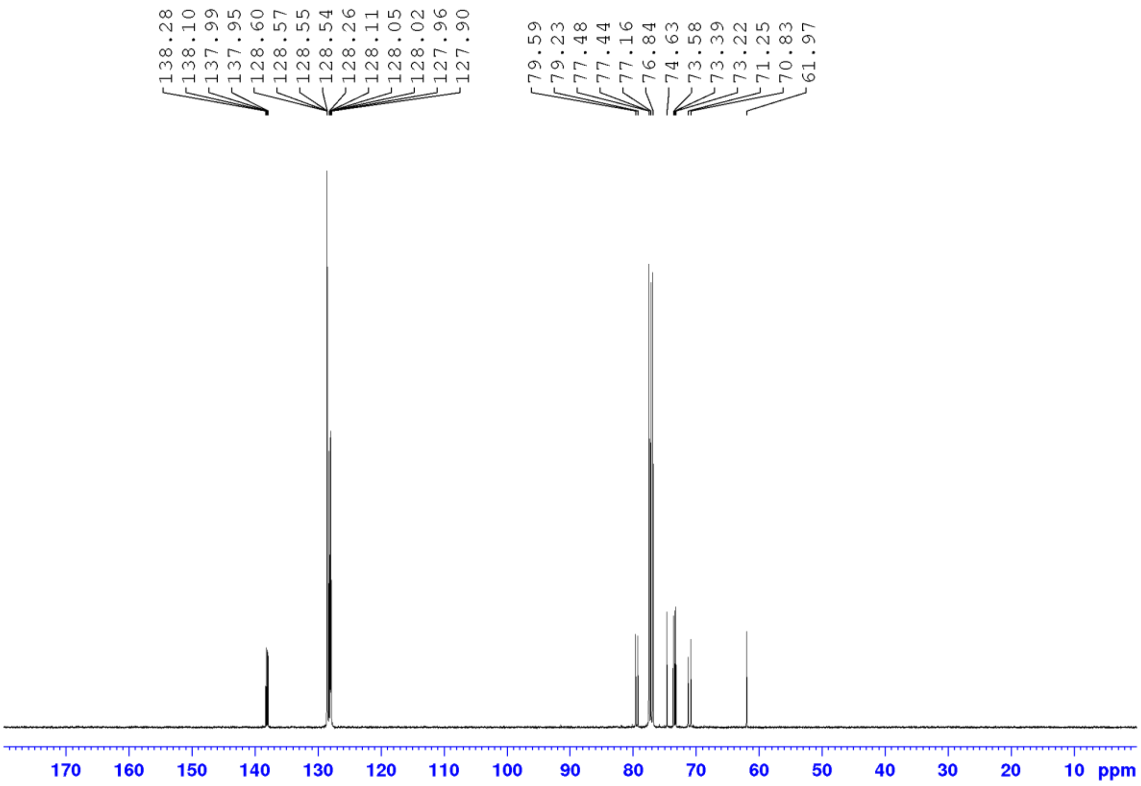

^{1}H-NMR spectra of compound **17** ($CDCl_3$, 400.13 MHz)

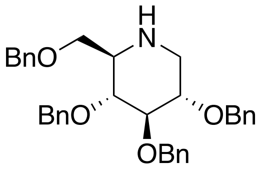

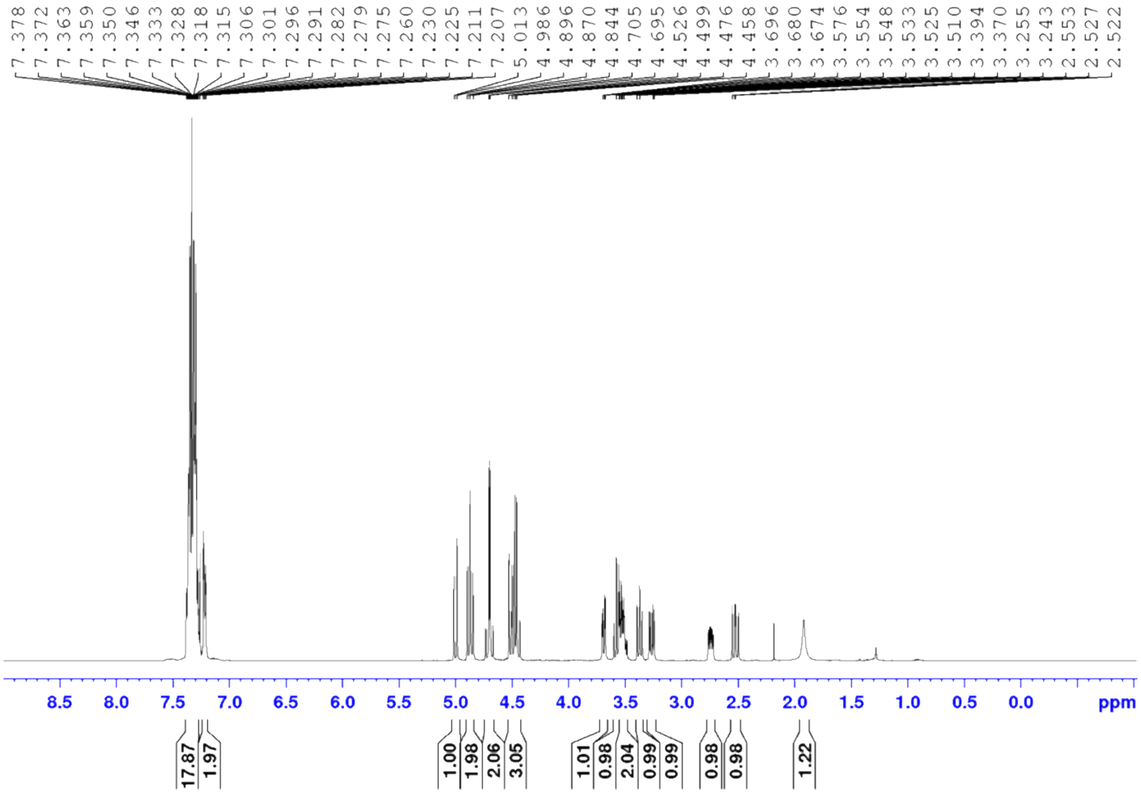

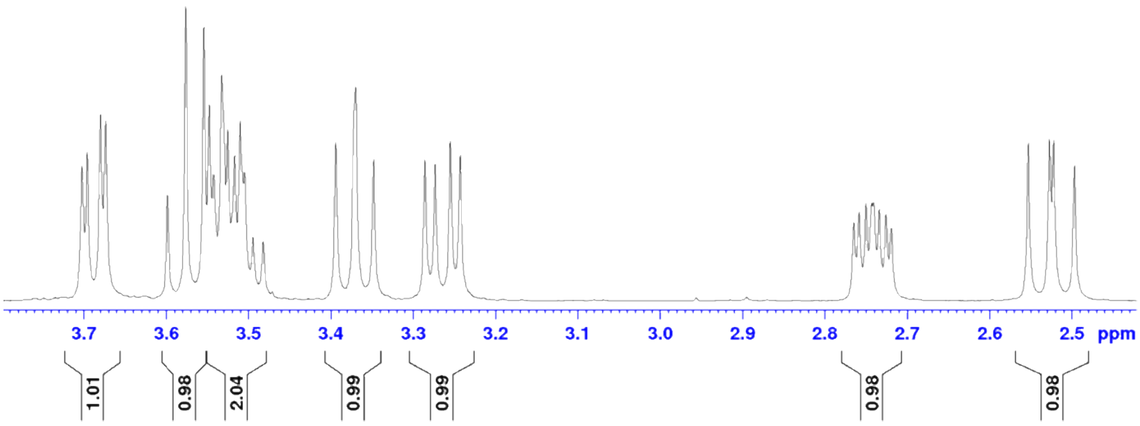

^{13}C-NMR spectra of compound **17** ($CDCl_3$, 100.61 MHz)

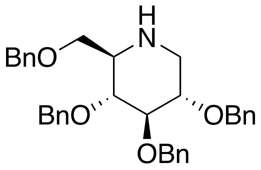

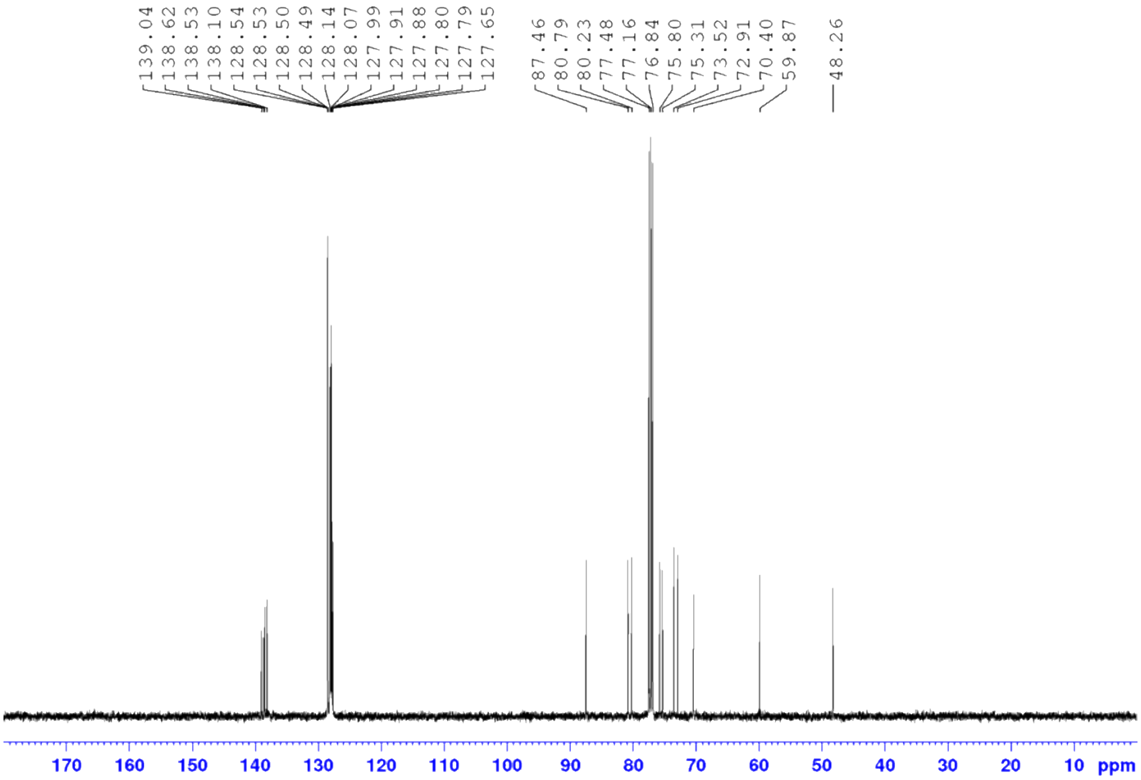

^{1}H-NMR spectra of compound **18a** ($CDCl_3$, 400.13 MHz)

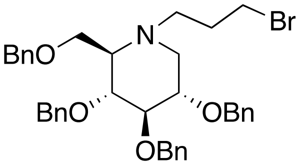

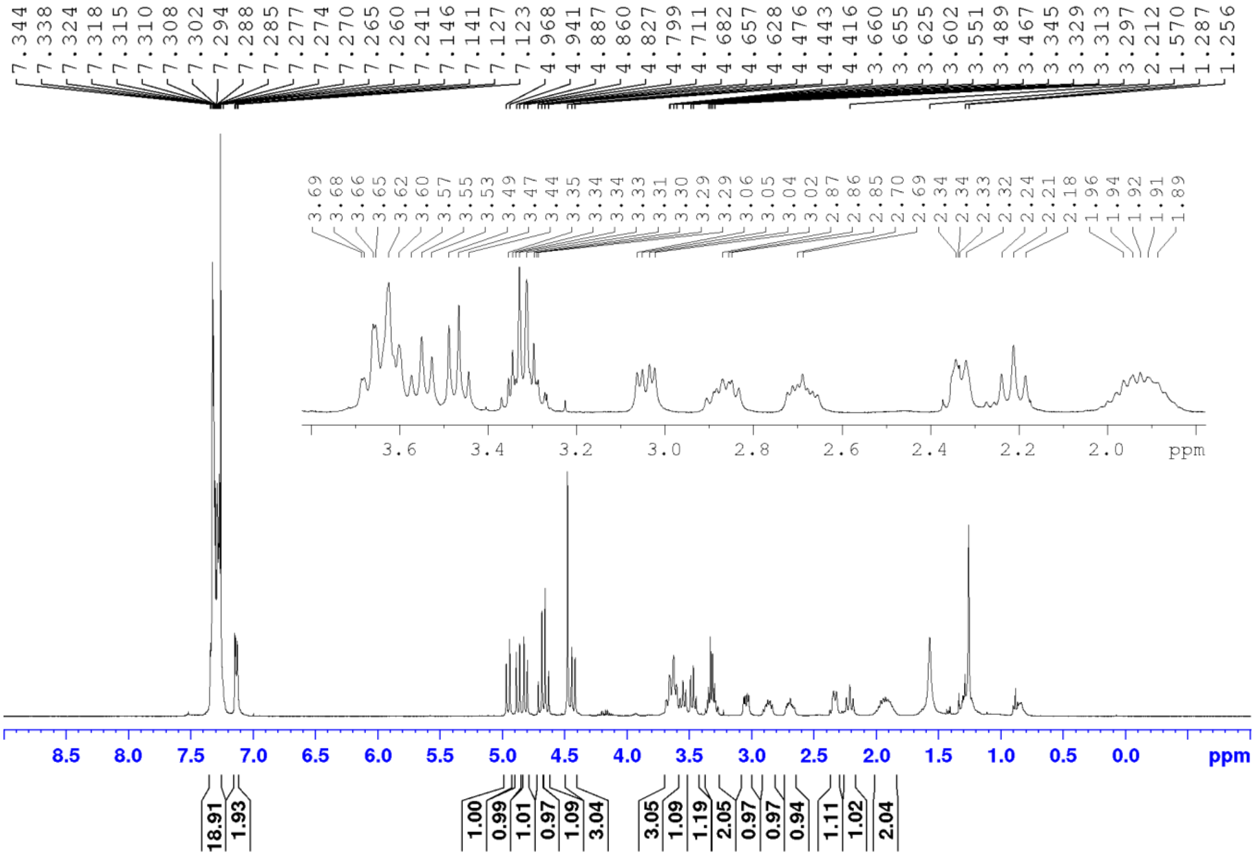

^{13}C-NMR spectra of compound **18a** ($CDCl_3$, 100.61 MHz)

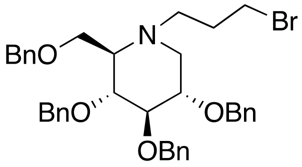

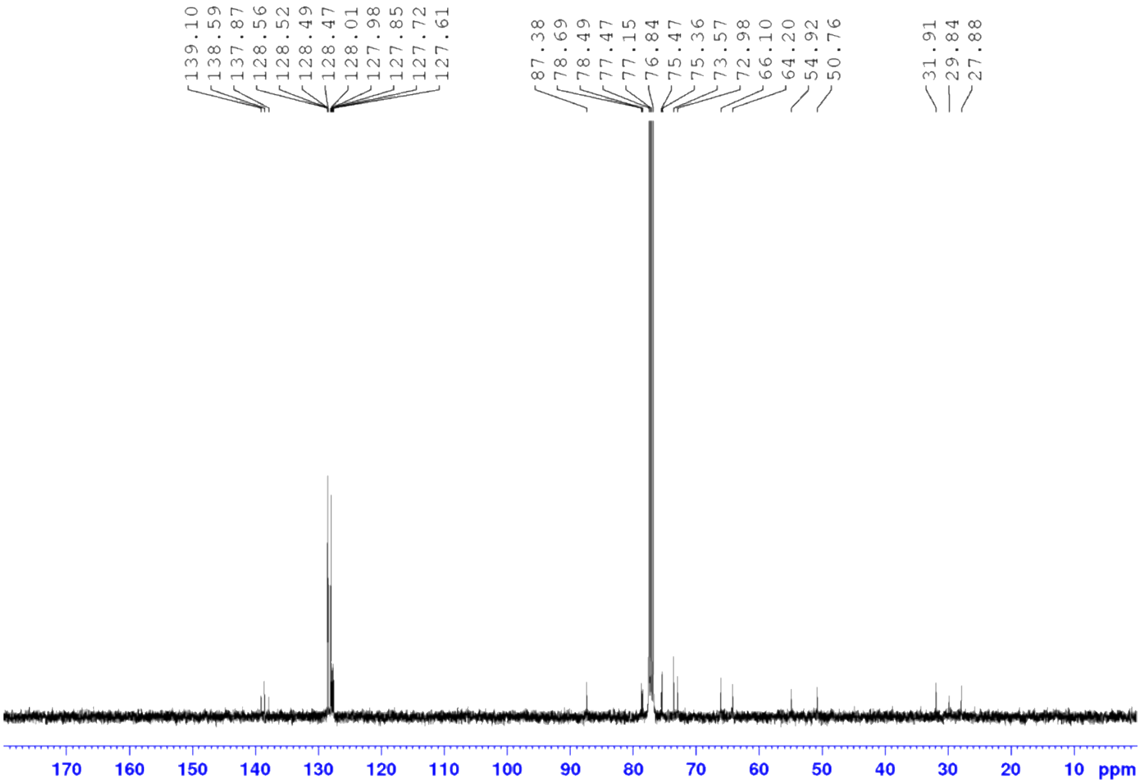

^{1}H-NMR spectra of compound **18b** ($CDCl_3$, 400.13 MHz)

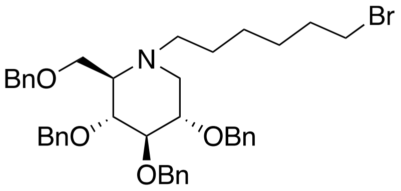

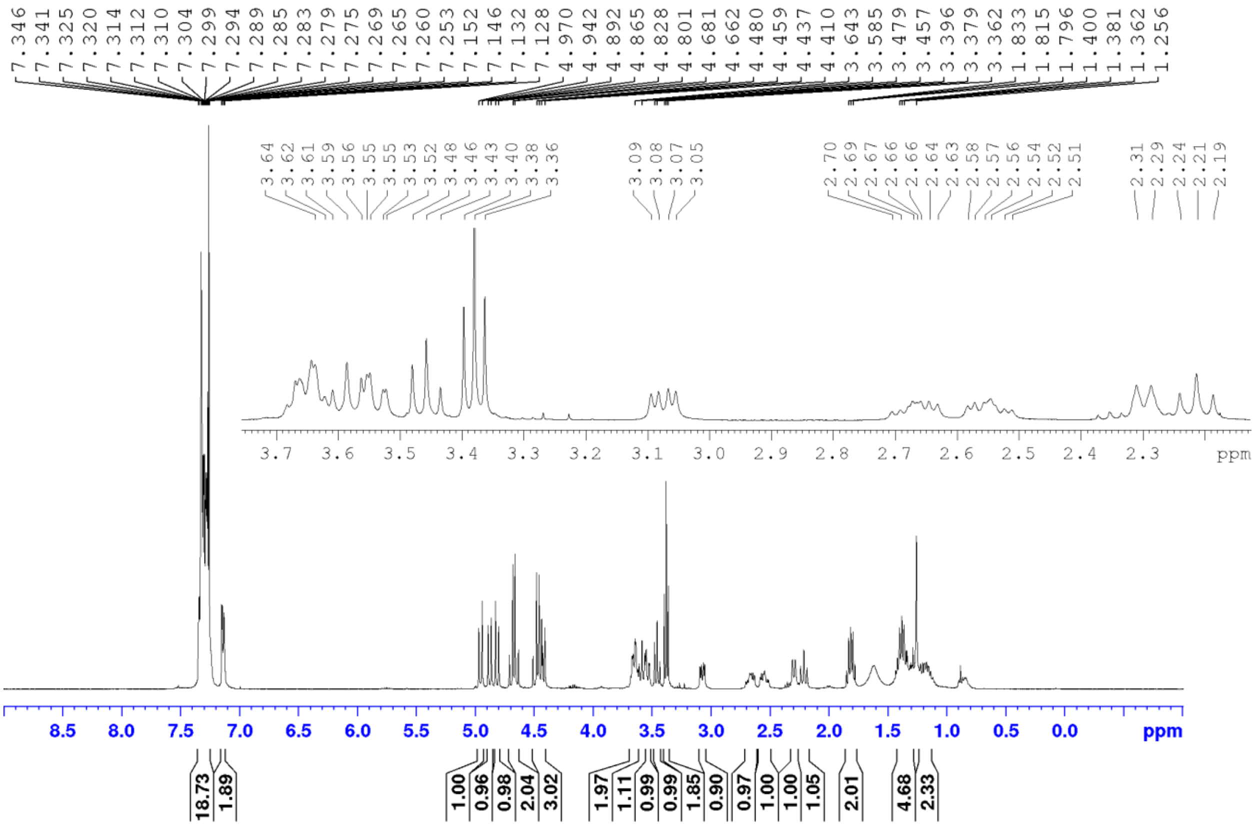

^{13}C-NMR spectra of compound **18b** ($CDCl_3$, 100.61 MHz)

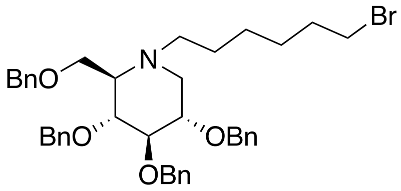

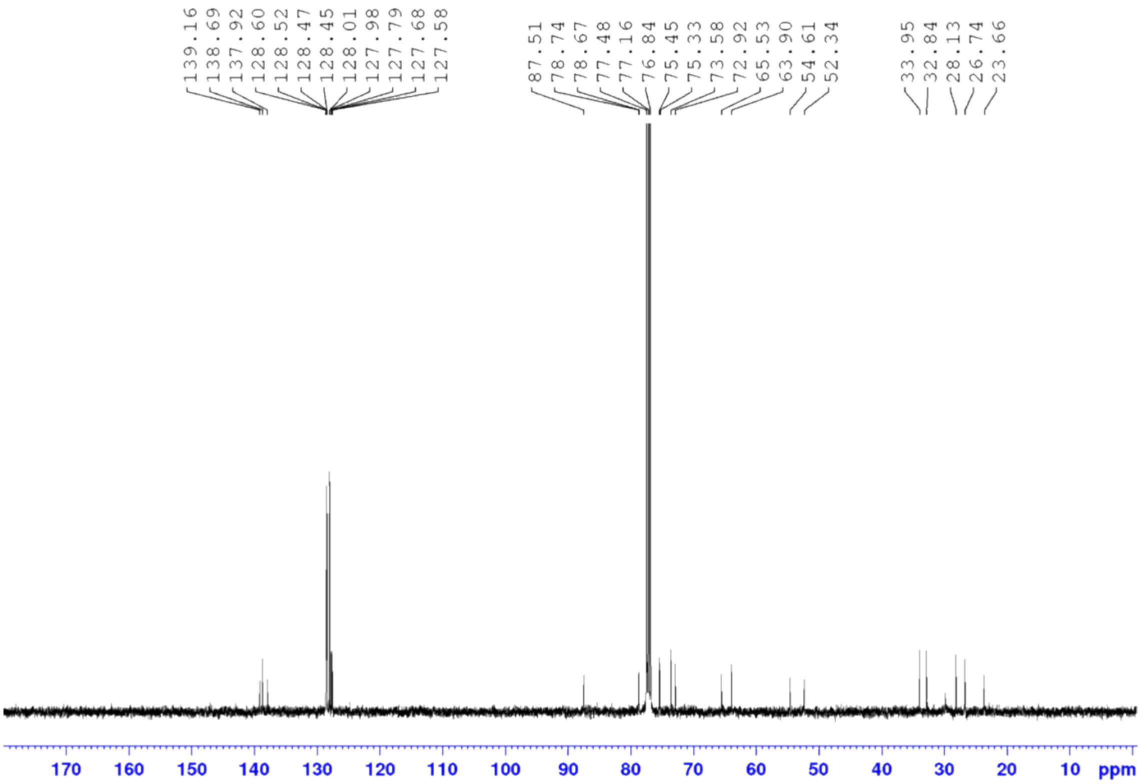

^{1}H-NMR spectra of compound **18c** ($CDCl_3$, 400.13 MHz)

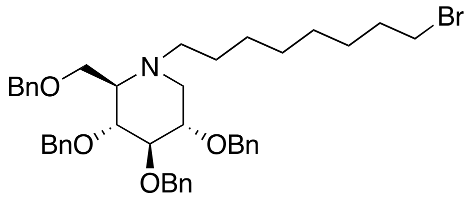

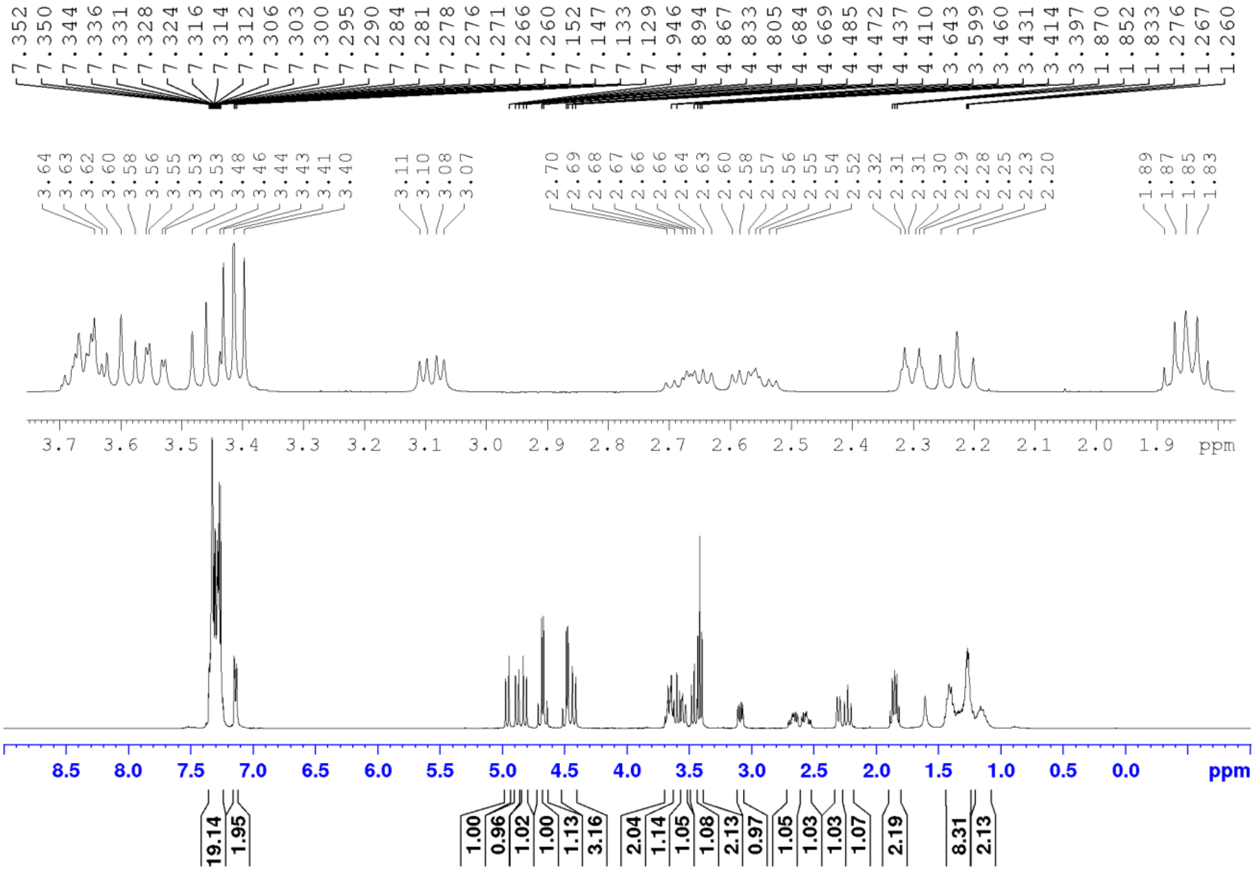

^{13}C-NMR spectra of compound **18c** ($CDCl_3$, 100.61 MHz)

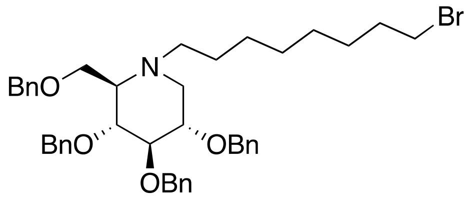

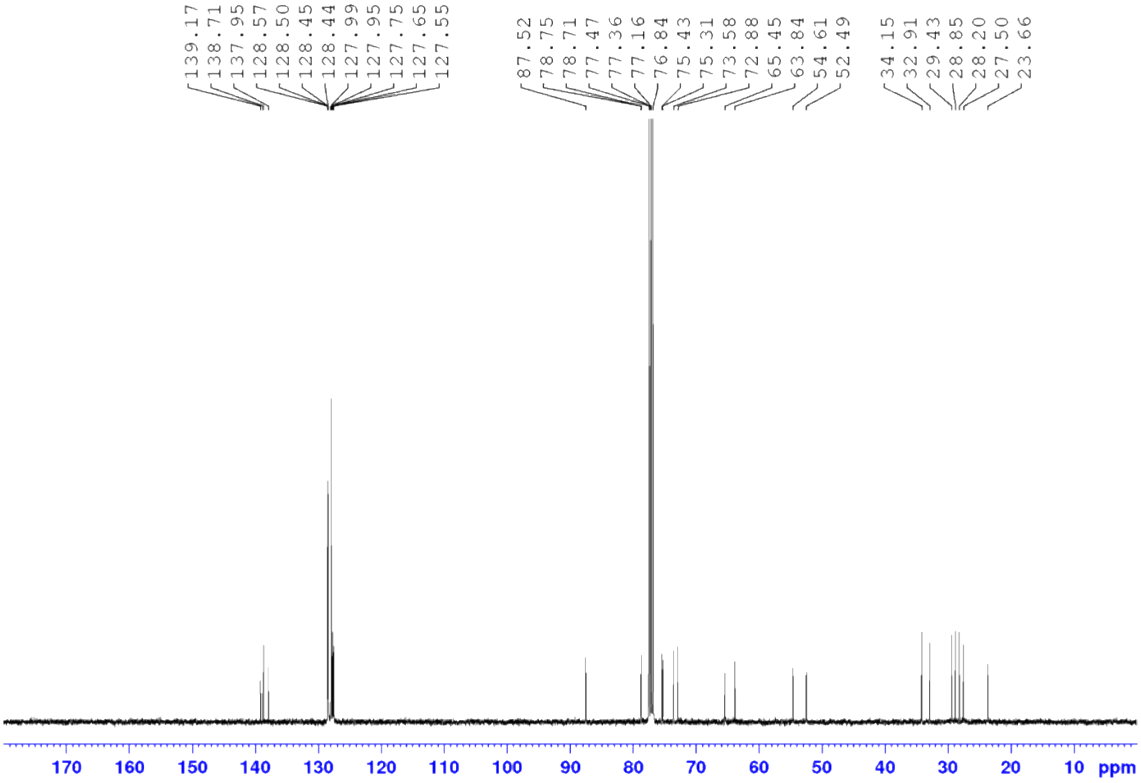

^{1}H-NMR spectra of compound **18d** ($CDCl_3$, 400.13 MHz)

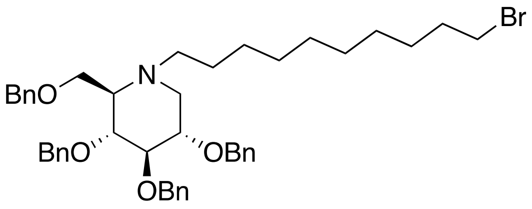

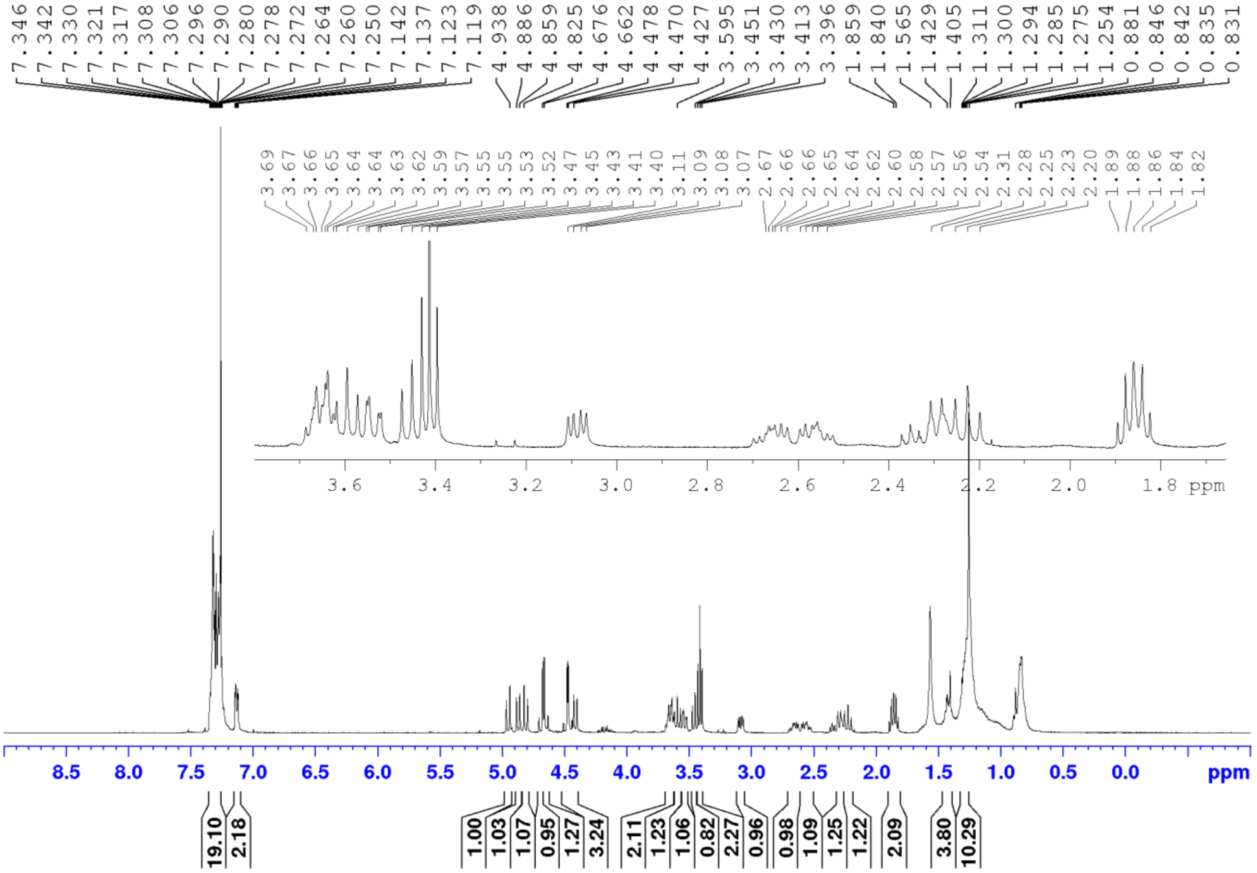

^{13}C-NMR spectra of compound **18d** ($CDCl_3$, 100.61 MHz)

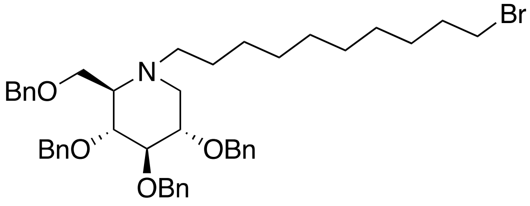

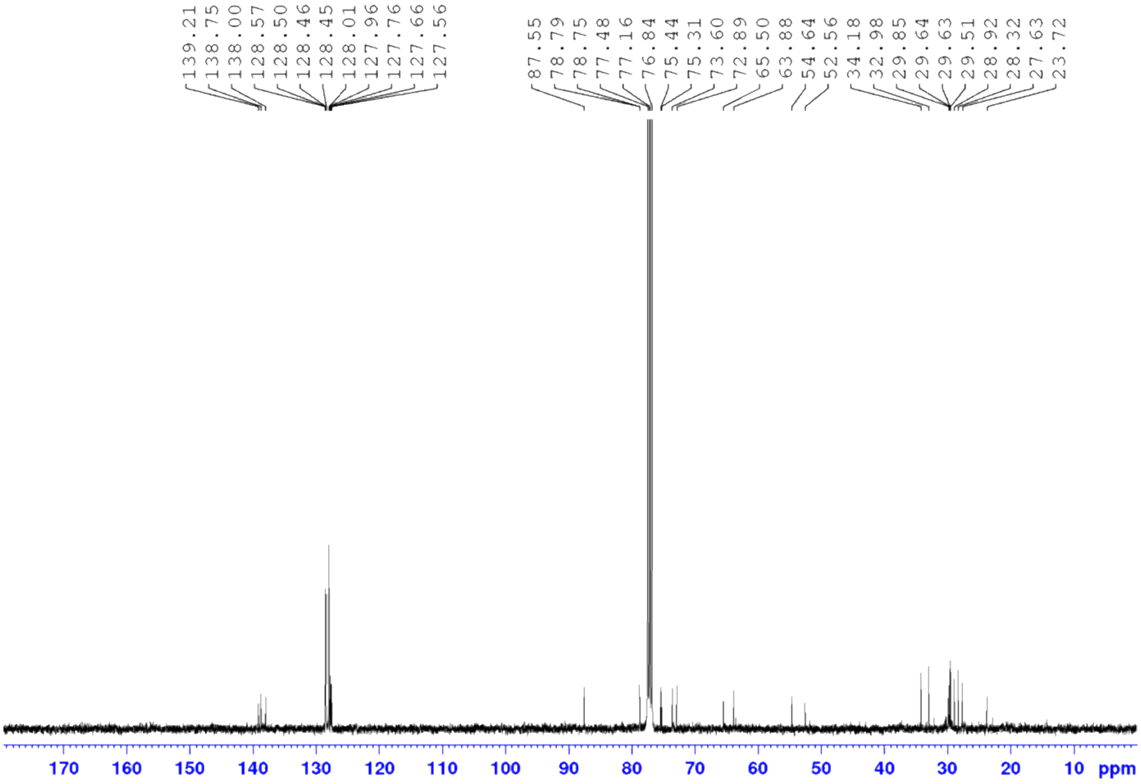

^{1}H-NMR spectra of compound **19a** ($CDCl_3$, 400.13 MHz)

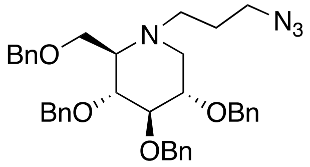

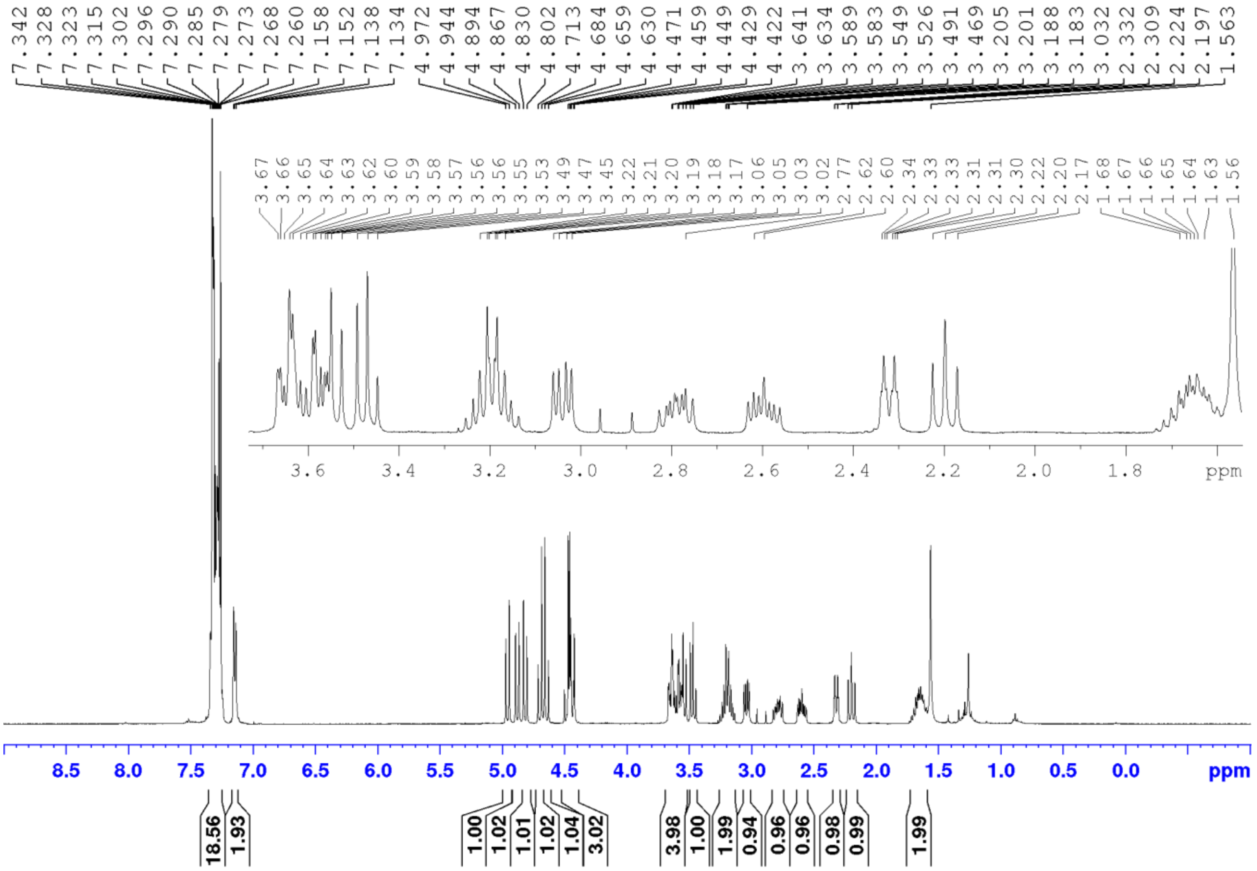

^{13}C-NMR spectra of compound **19a** ($CDCl_3$, 100.61 MHz)

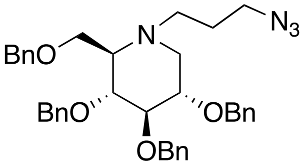

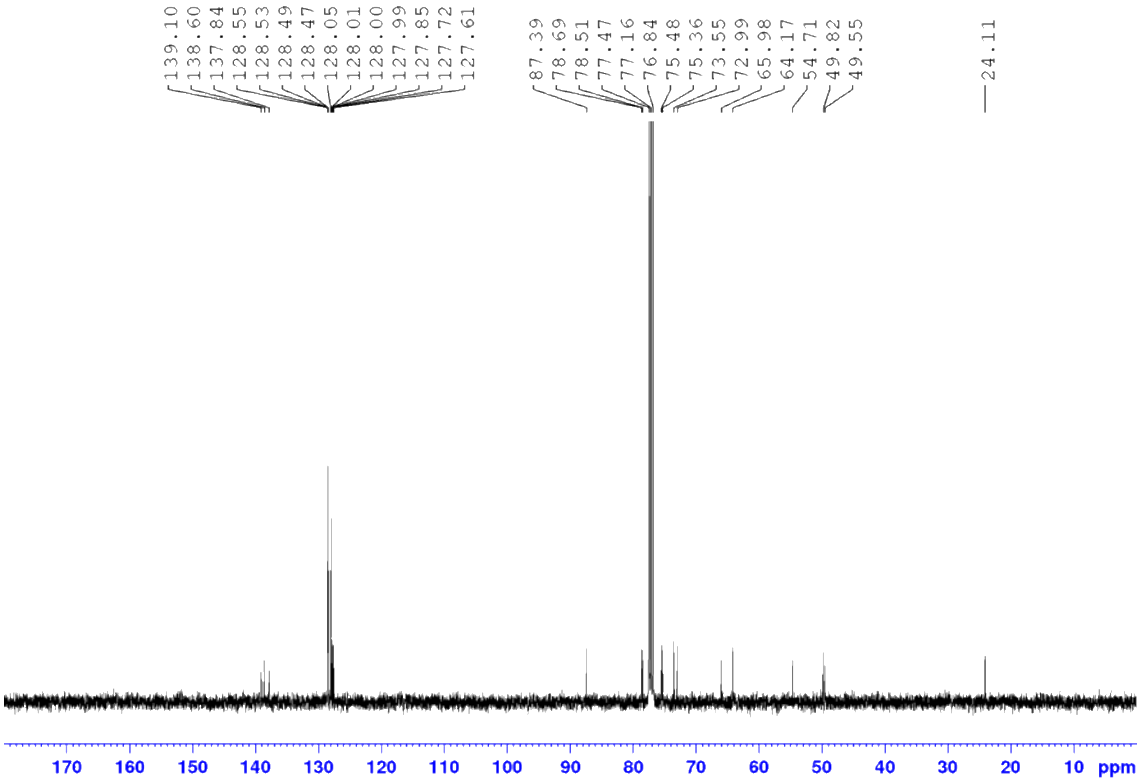

^{1}H-NMR spectra of compound **19b** ($CDCl_3$, 400.13 MHz)

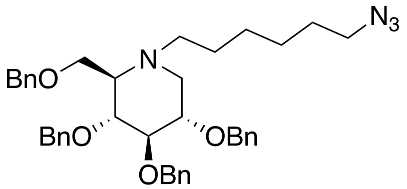

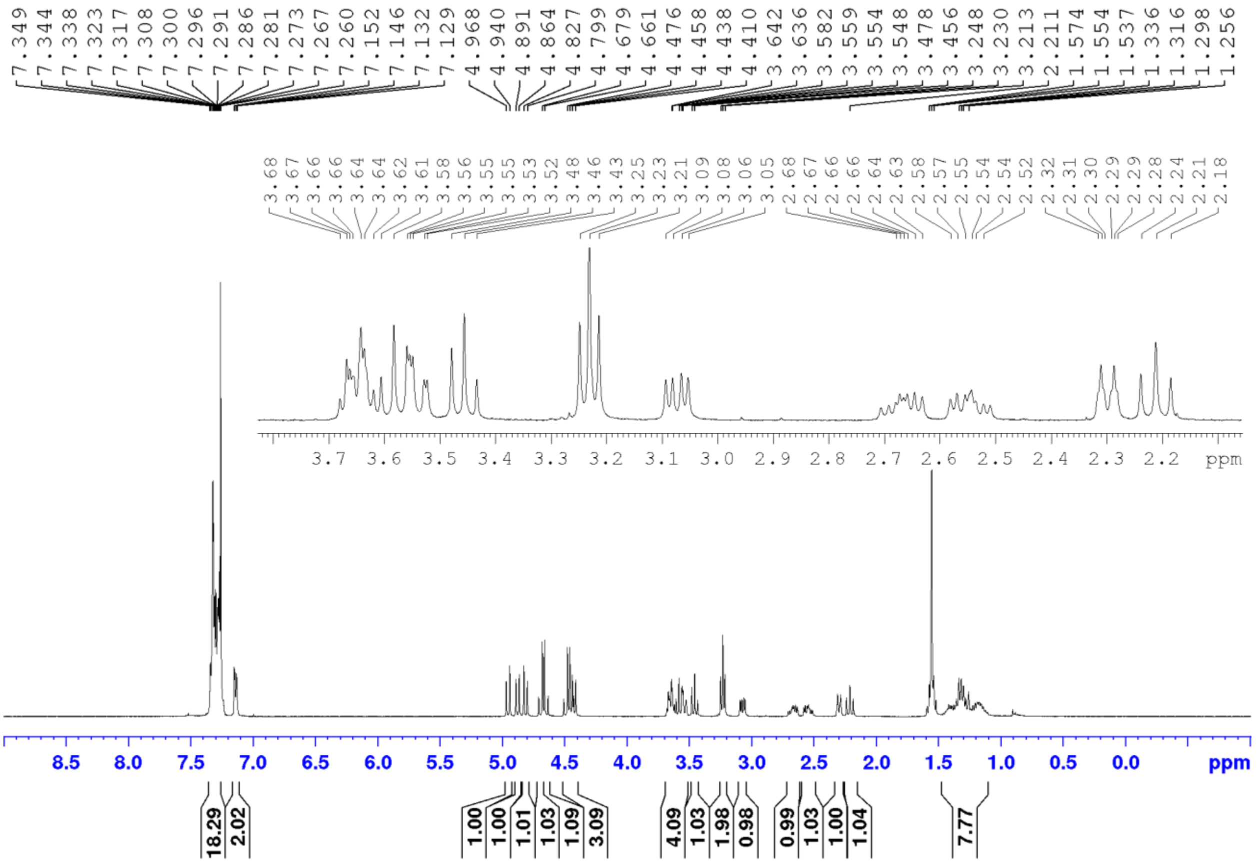

^{13}C-NMR spectra of compound **19b** ($CDCl_3$, 100.61 MHz)

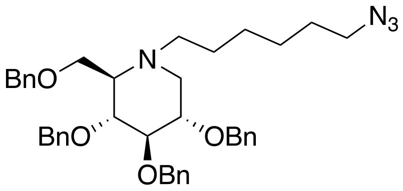

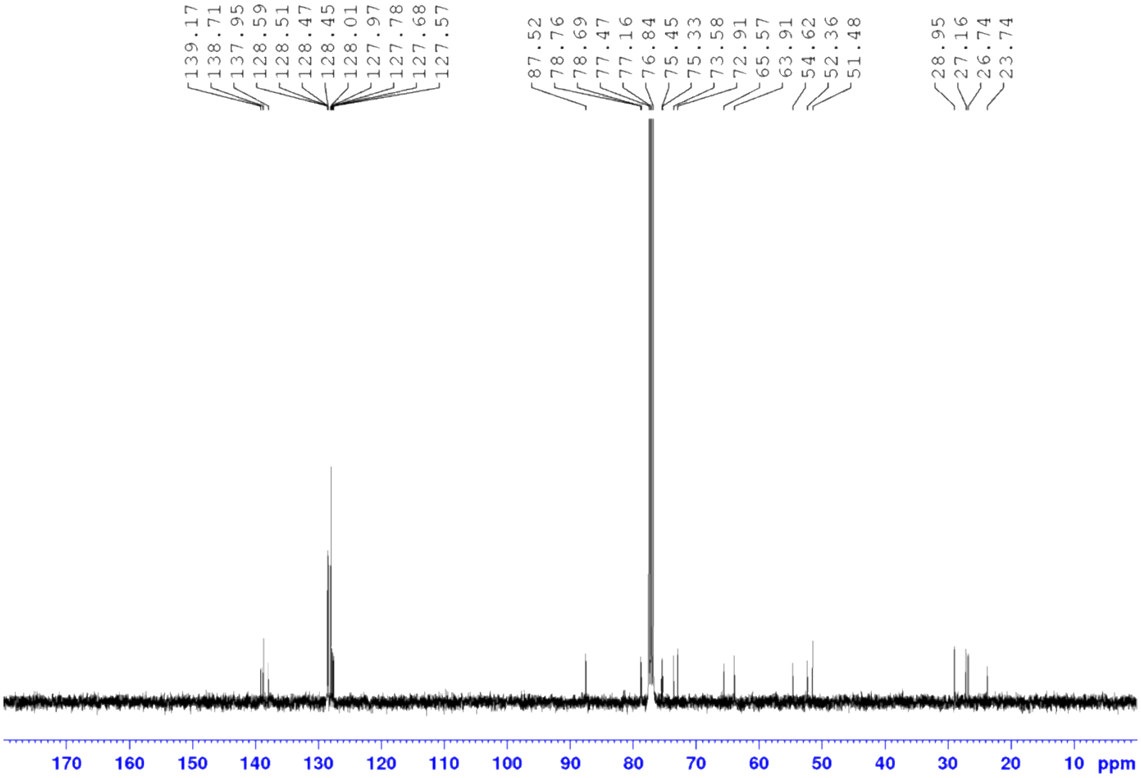

^{1}H-NMR spectra of compound **19c** ($CDCl_3$, 400.13 MHz)

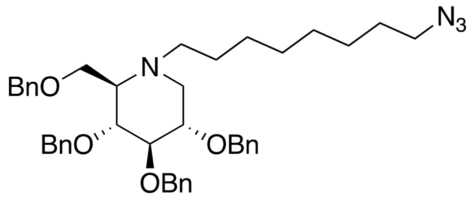

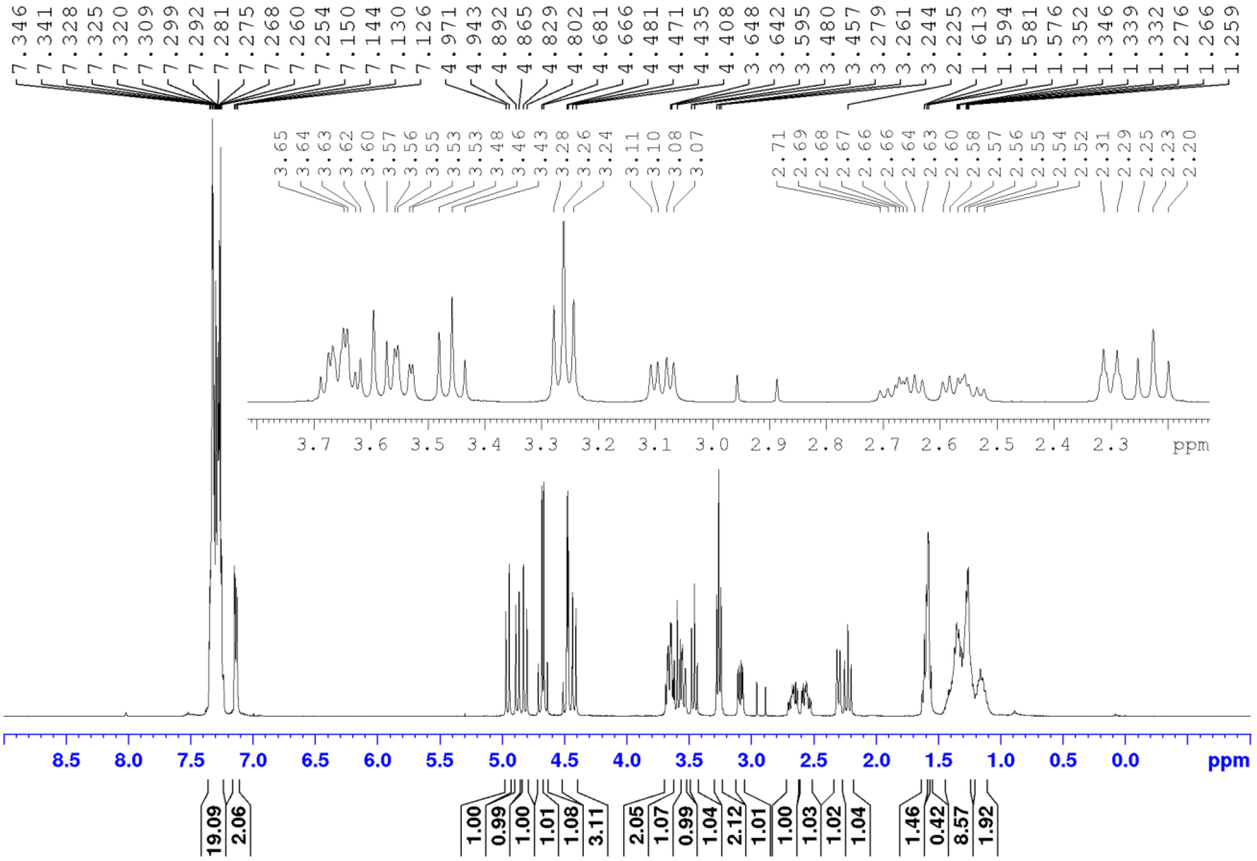

^{13}C-NMR spectra of compound **19c** ($CDCl_3$, 100.61 MHz)

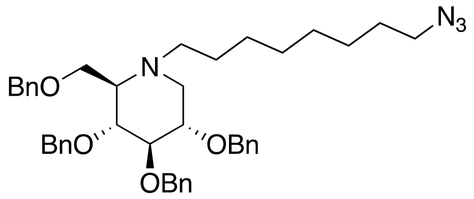

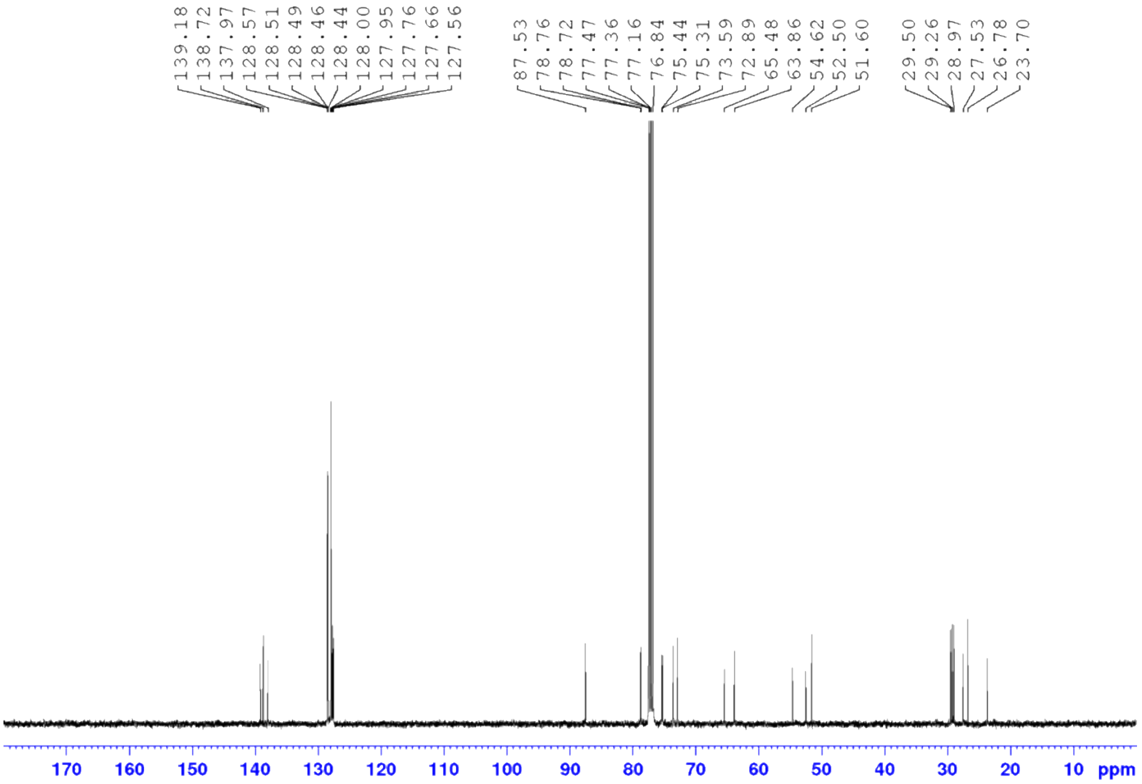

^{1}H-NMR spectra of compound **19d** ($CDCl_3$, 400.13 MHz)

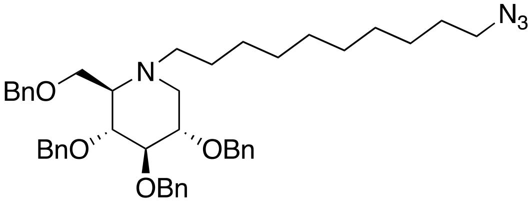

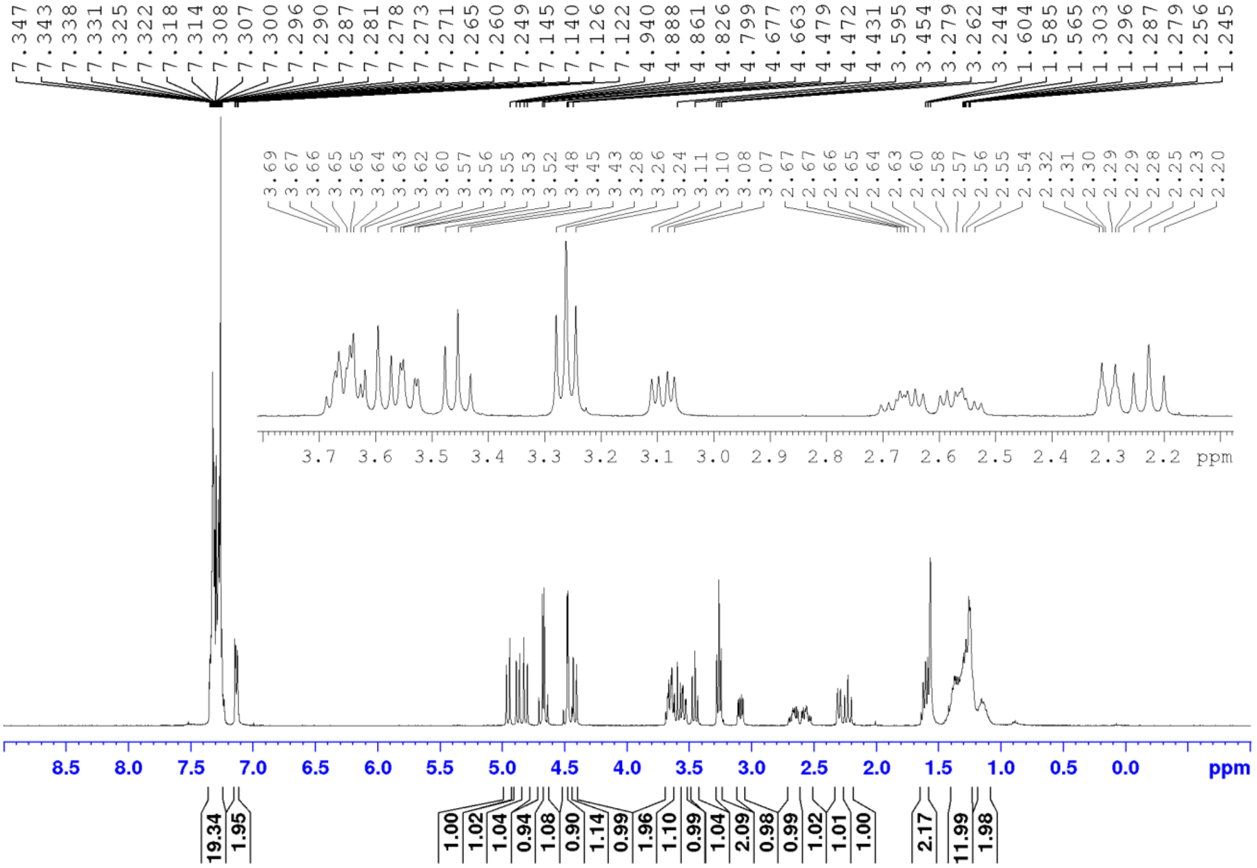

^{13}C-NMR spectra of compound **19d** ($CDCl_3$, 100.61 MHz)

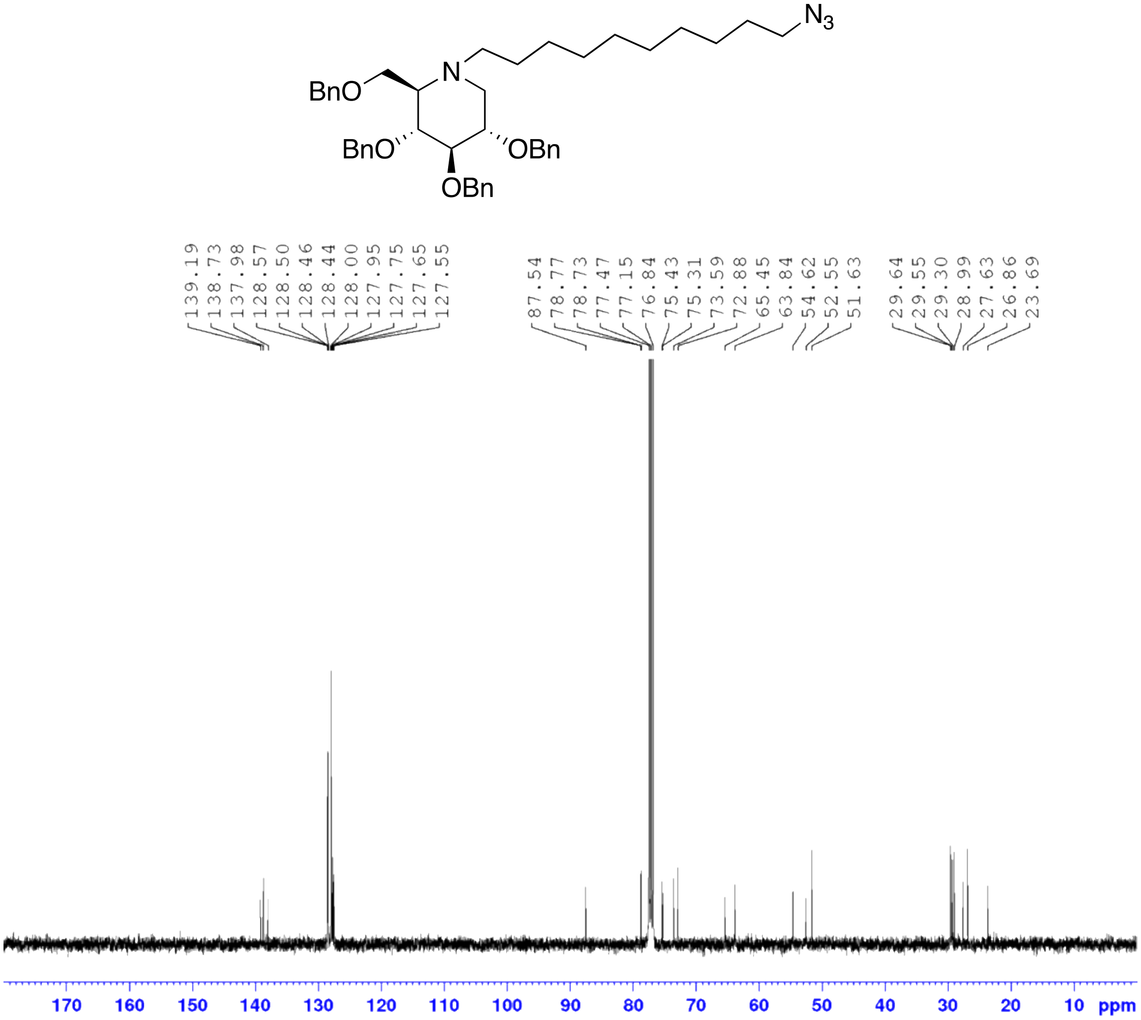

^{1}H-NMR spectra of compound **20** ($CDCl_3$, 400.13 MHz)

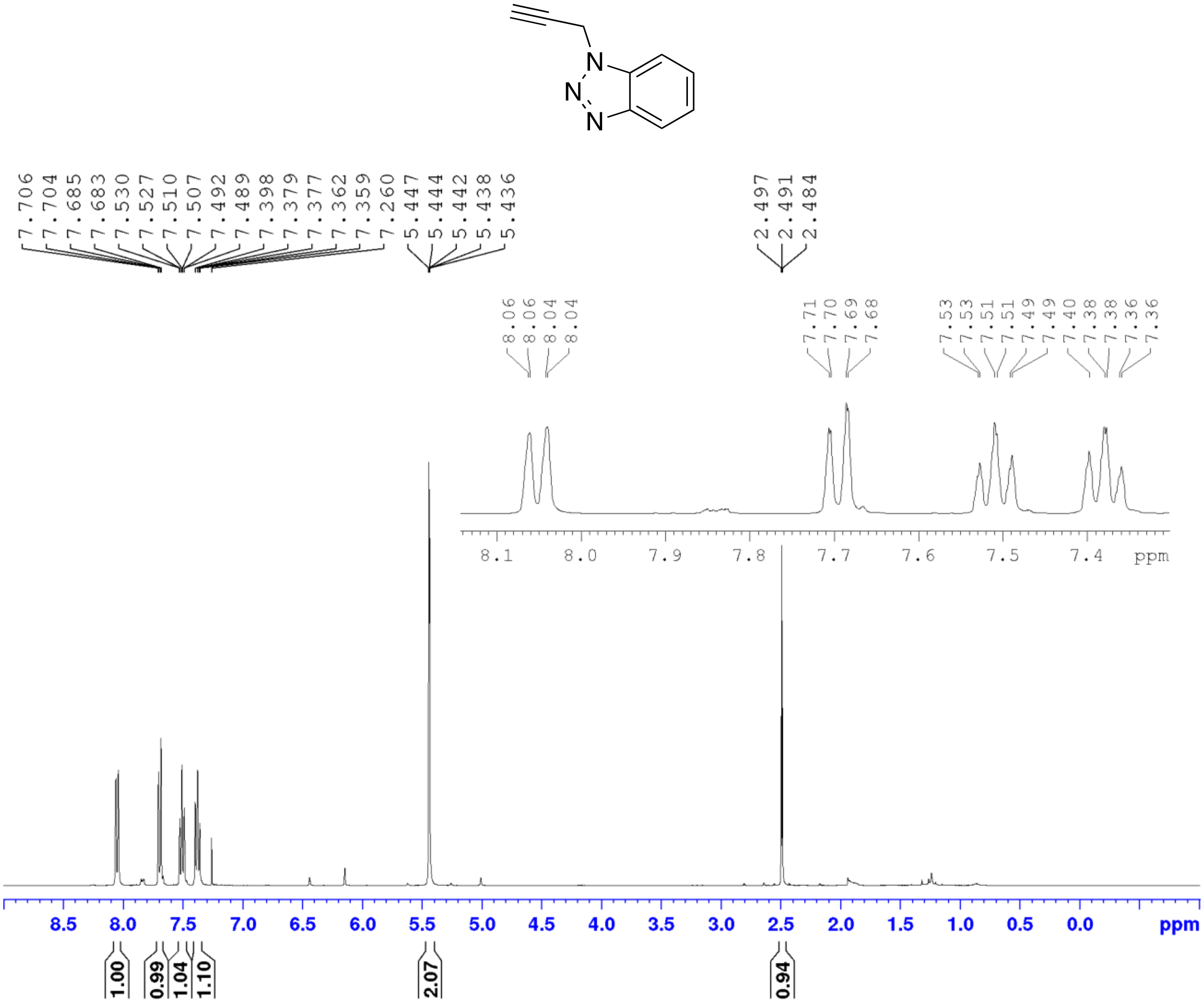

^{13}C-NMR spectra of compound **20** ($CDCl_3$, 100.61 MHz)

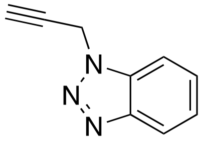

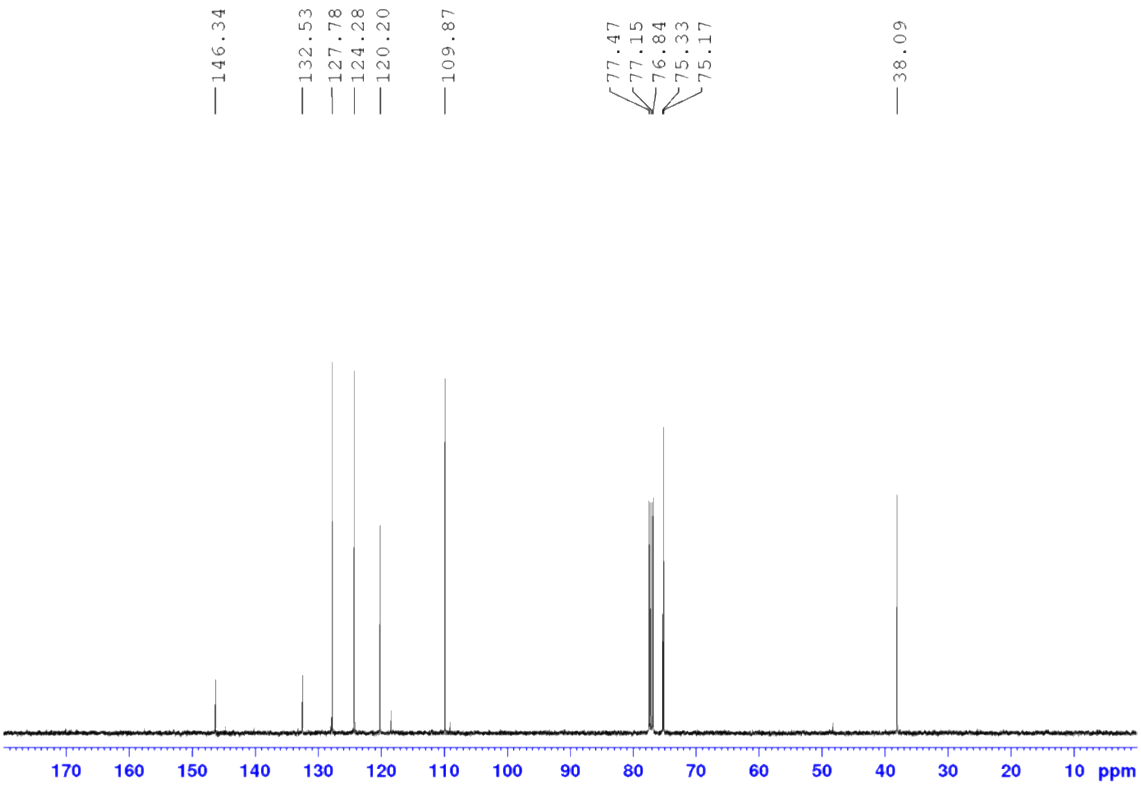

^{1}H-NMR spectra of compound **21a** ($CDCl_3$, 400.13 MHz)

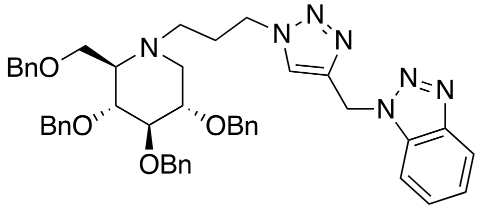

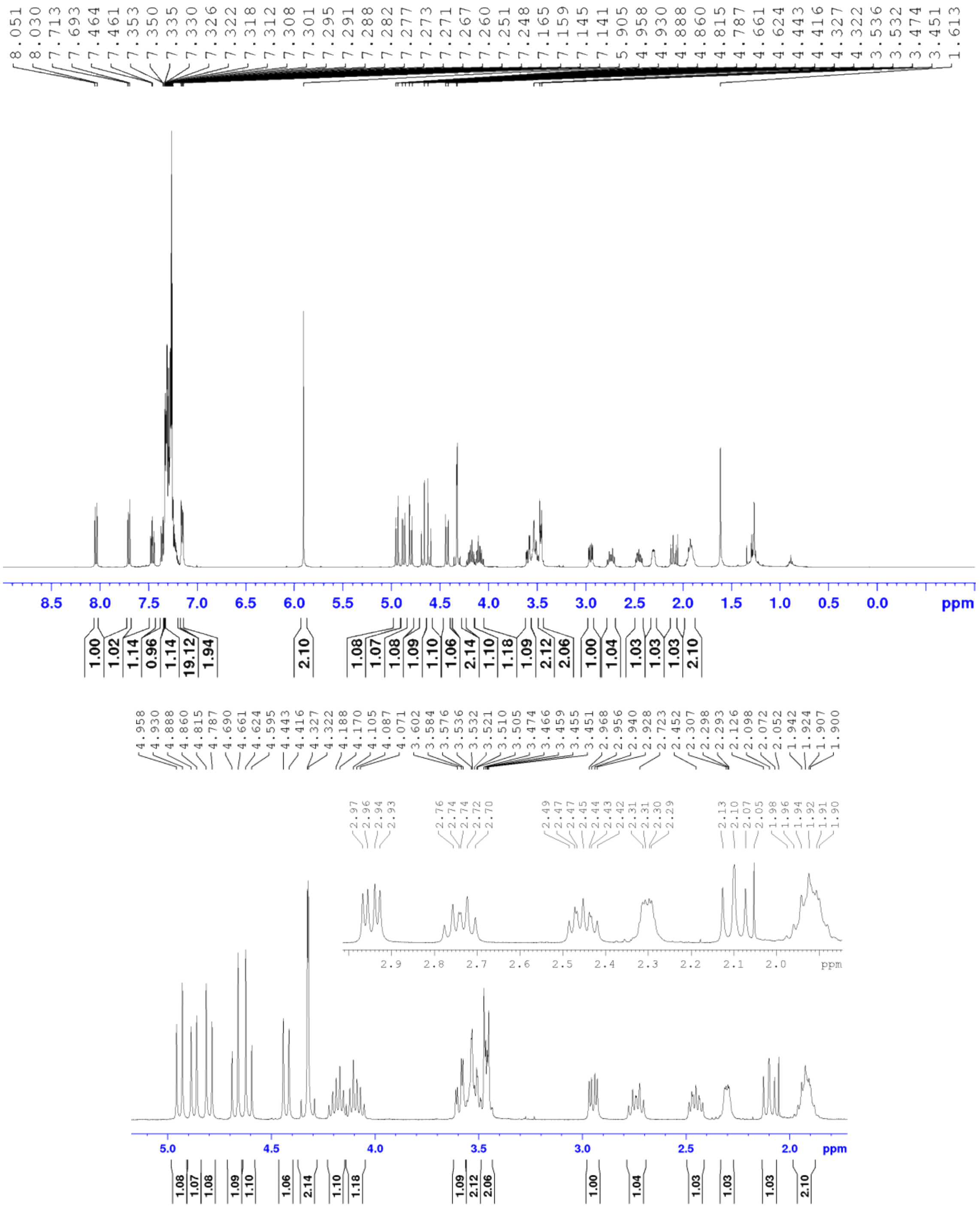

^{13}C-NMR spectra of compound **21a** ($CDCl_3$, 100.61 MHz)

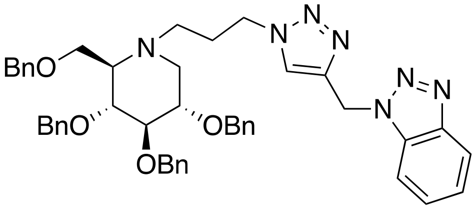

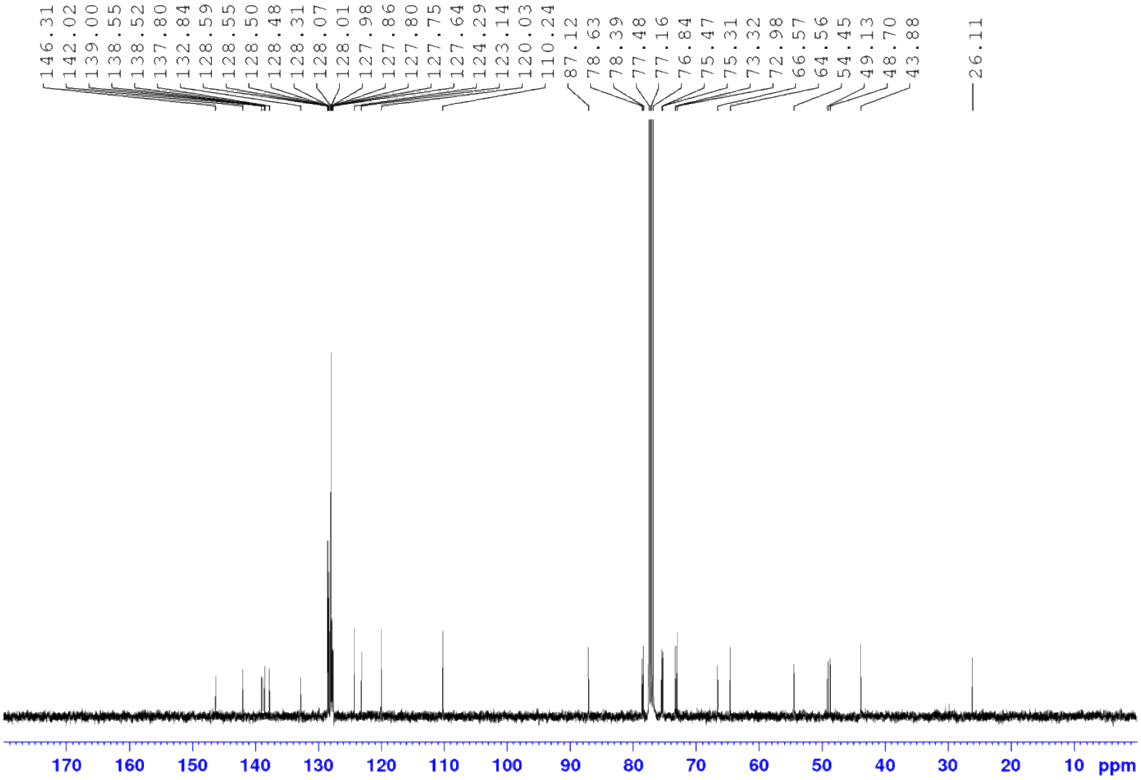

^{1}H-NMR spectra of compound **21b** ($CDCl_3$, 400.13 MHz)

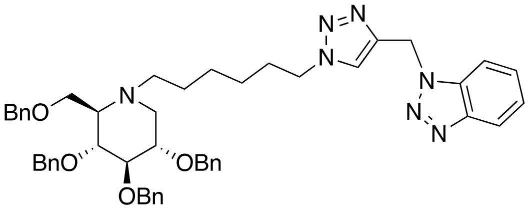

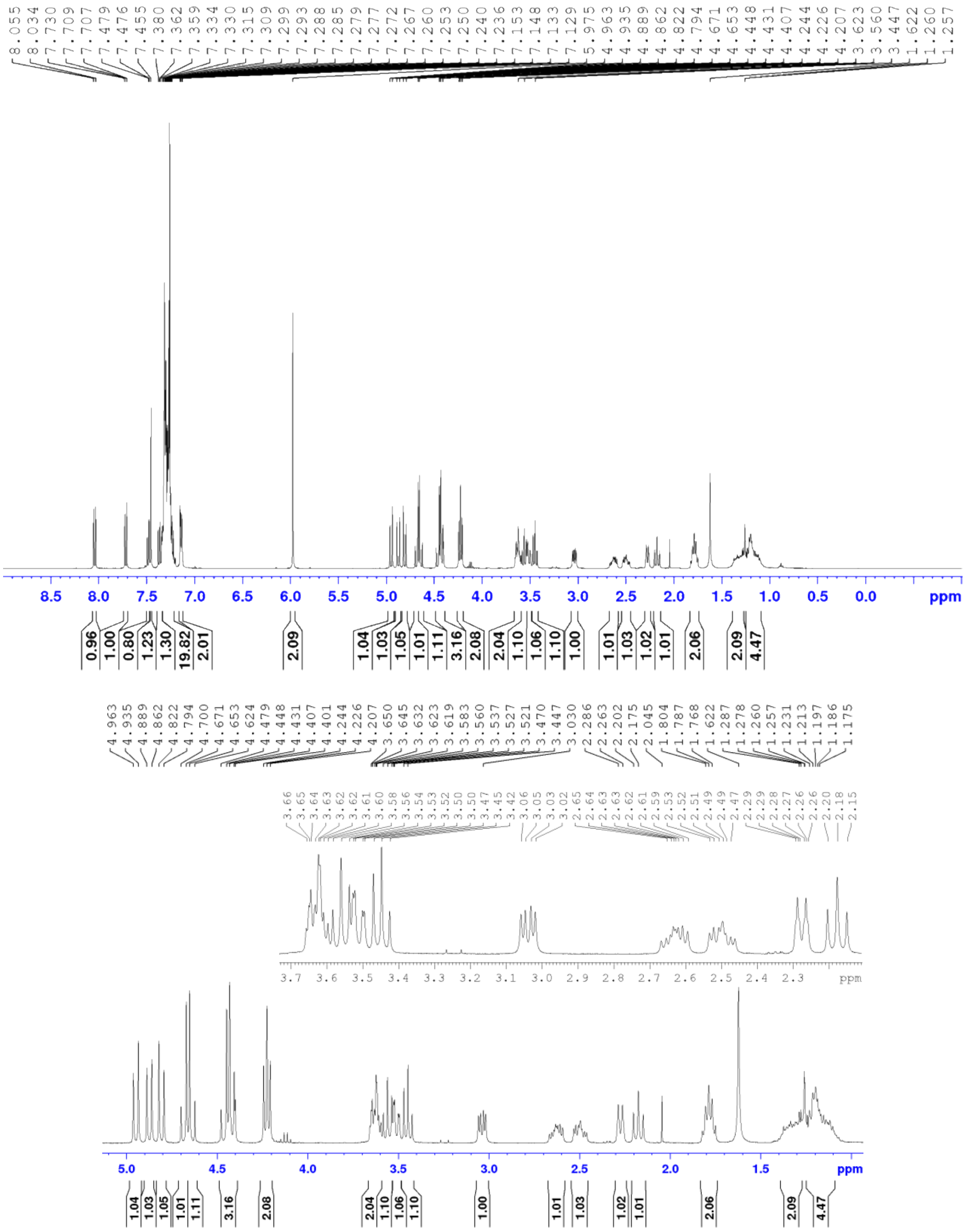

^{13}C-NMR spectra of compound **21b** ($CDCl_3$, 100.61 MHz)

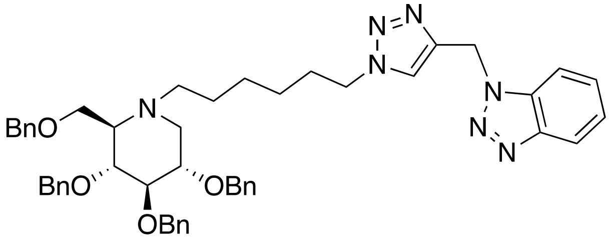

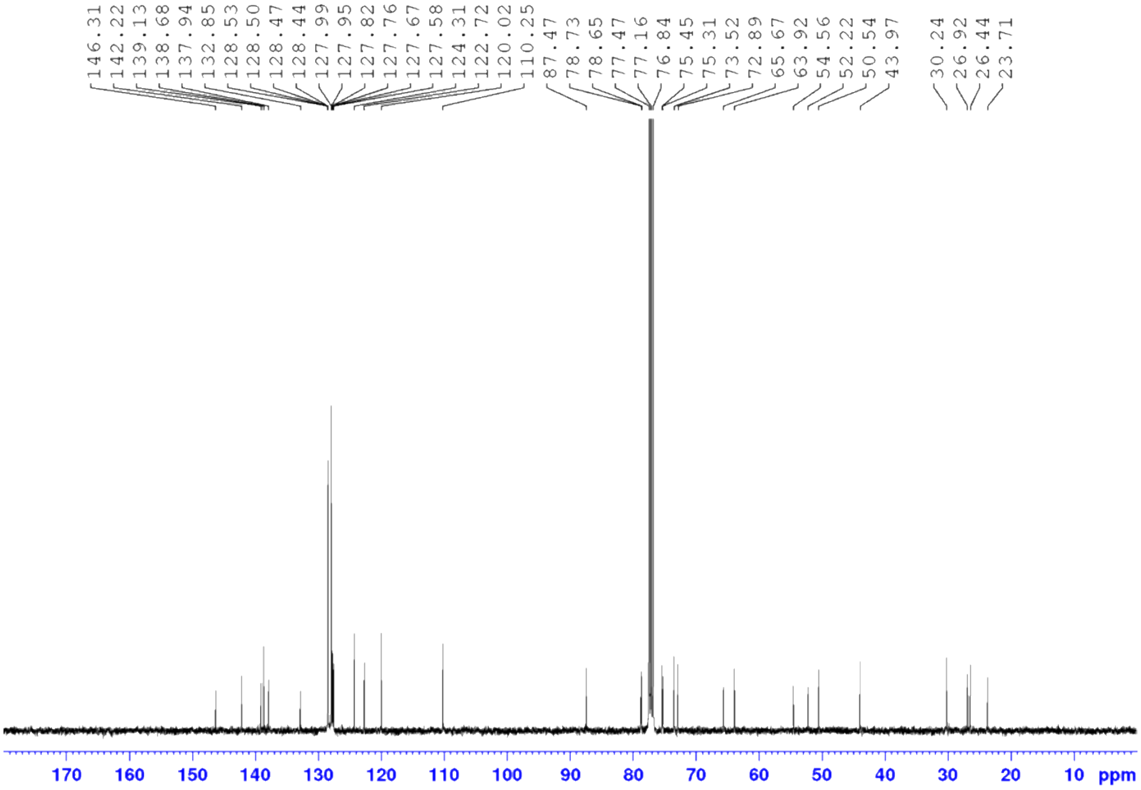

^{1}H-NMR spectra of compound **21c** ($CDCl_3$, 400.13 MHz)

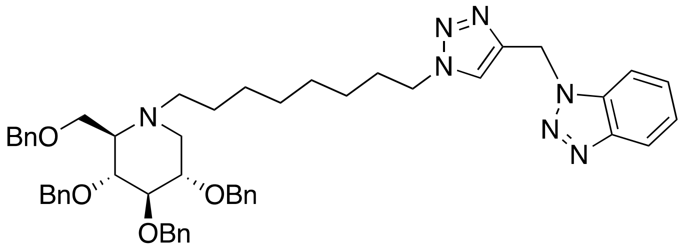

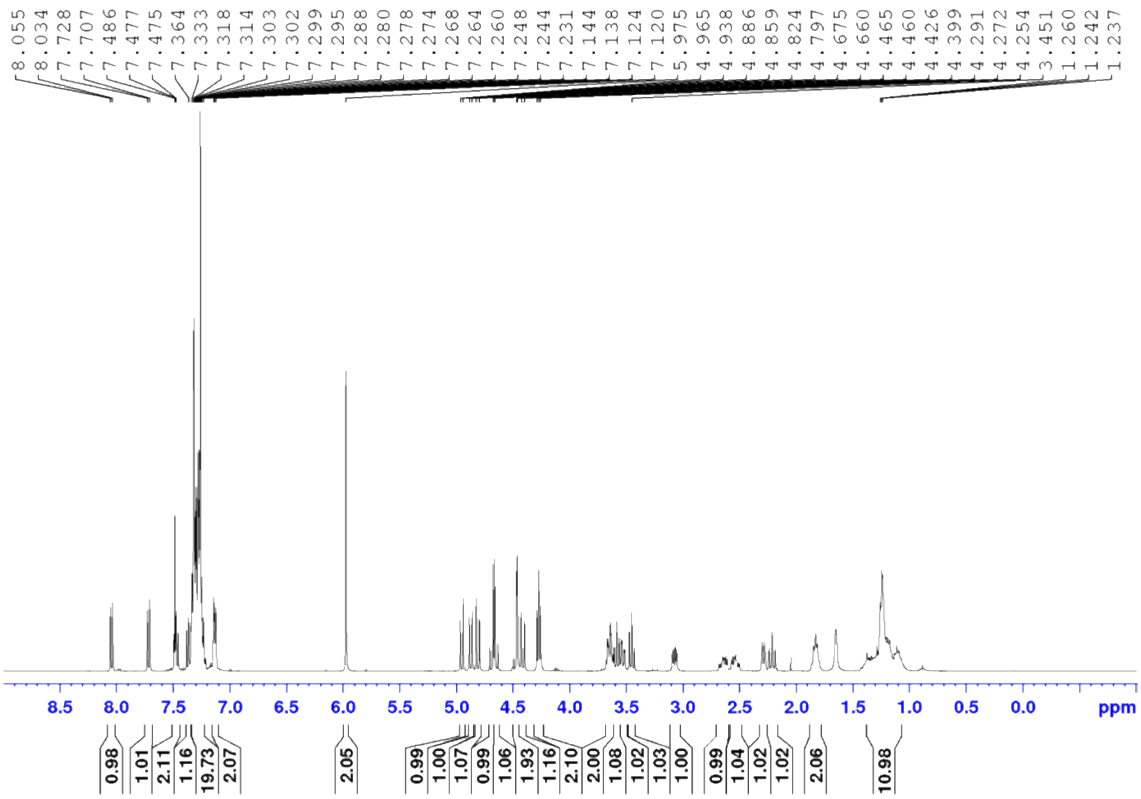

^{13}C-NMR spectra of compound **21c** ($CDCl_3$, 100.61 MHz)

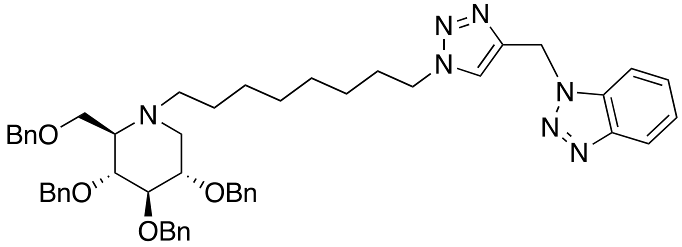

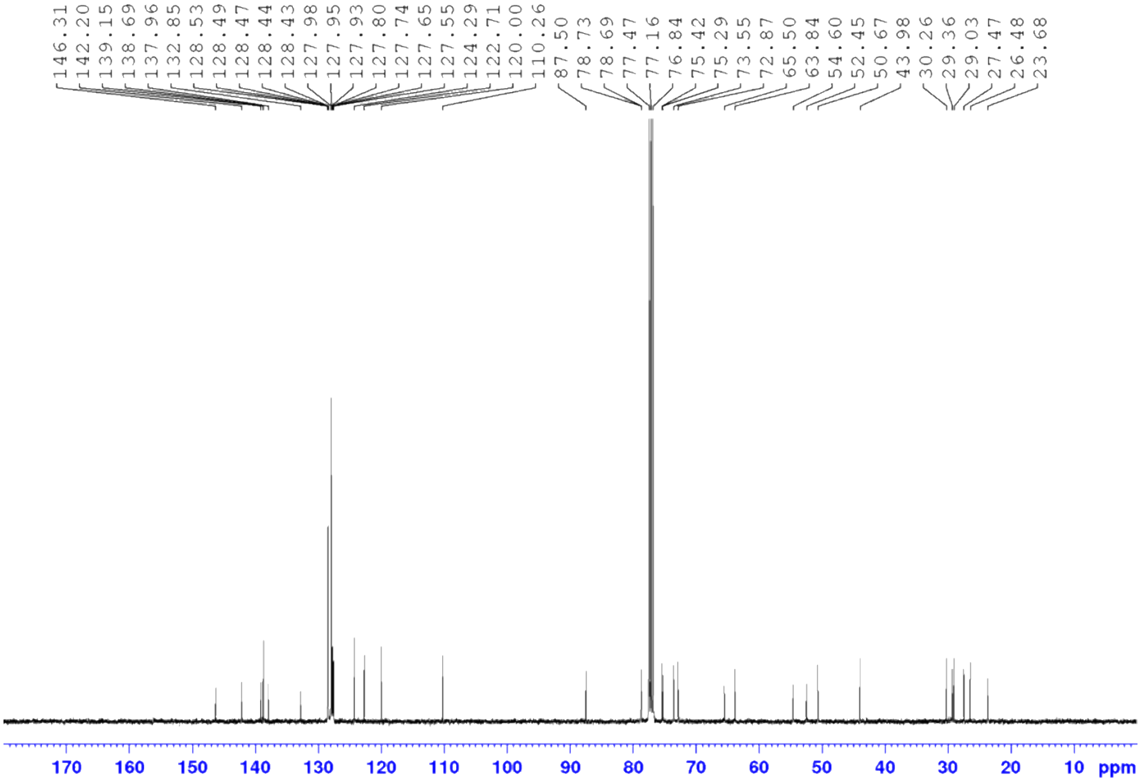

^{1}H-NMR spectra of compound **21d** ($CDCl_3$, 400.13 MHz)

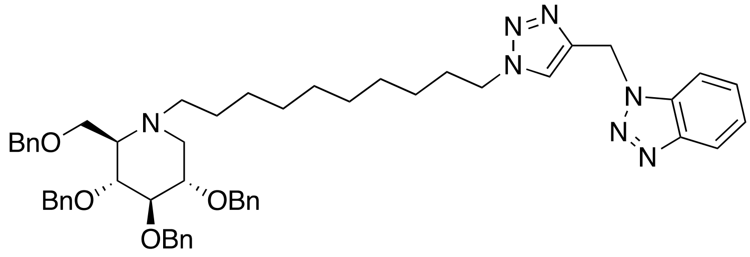

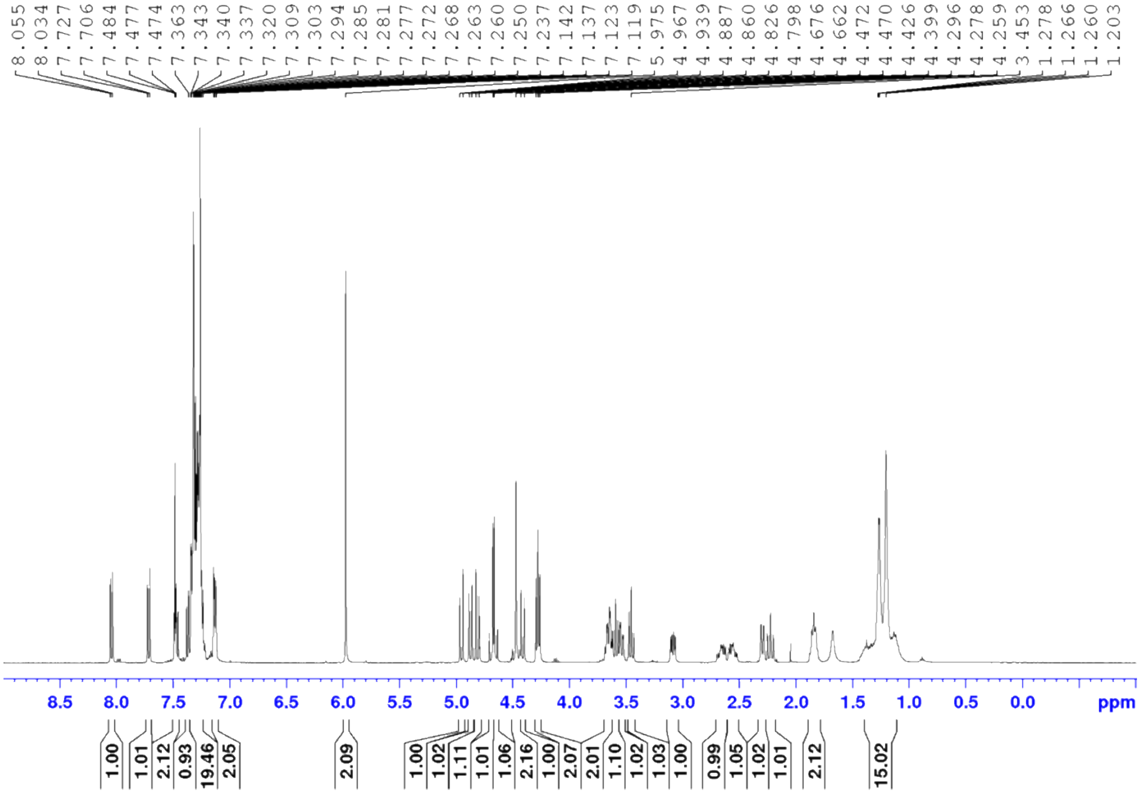

^{13}C-NMR spectra of compound **21d** ($CDCl_3$, 100.61 MHz)

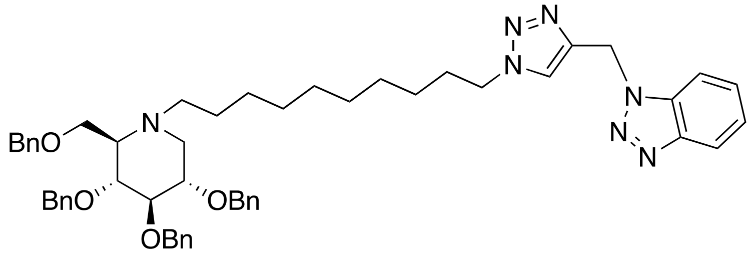

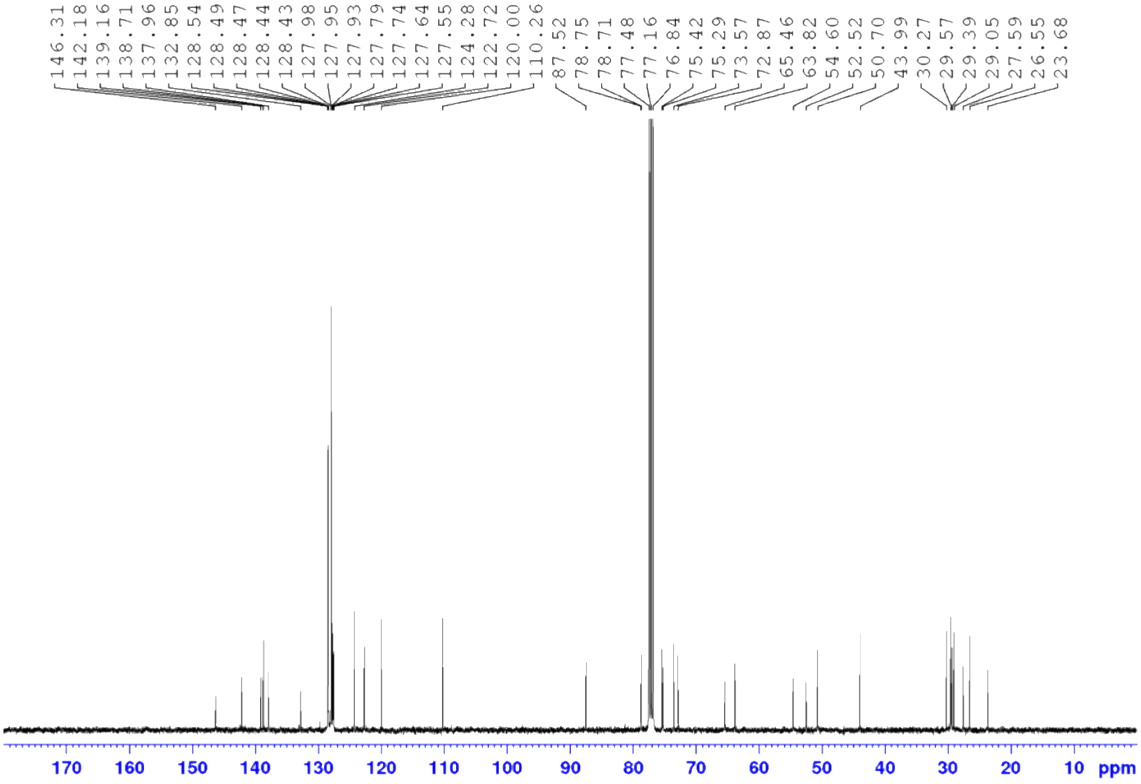

^{1}H-NMR spectra of compound **12a** (MeOD, 400.13 MHz)

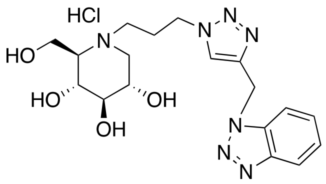

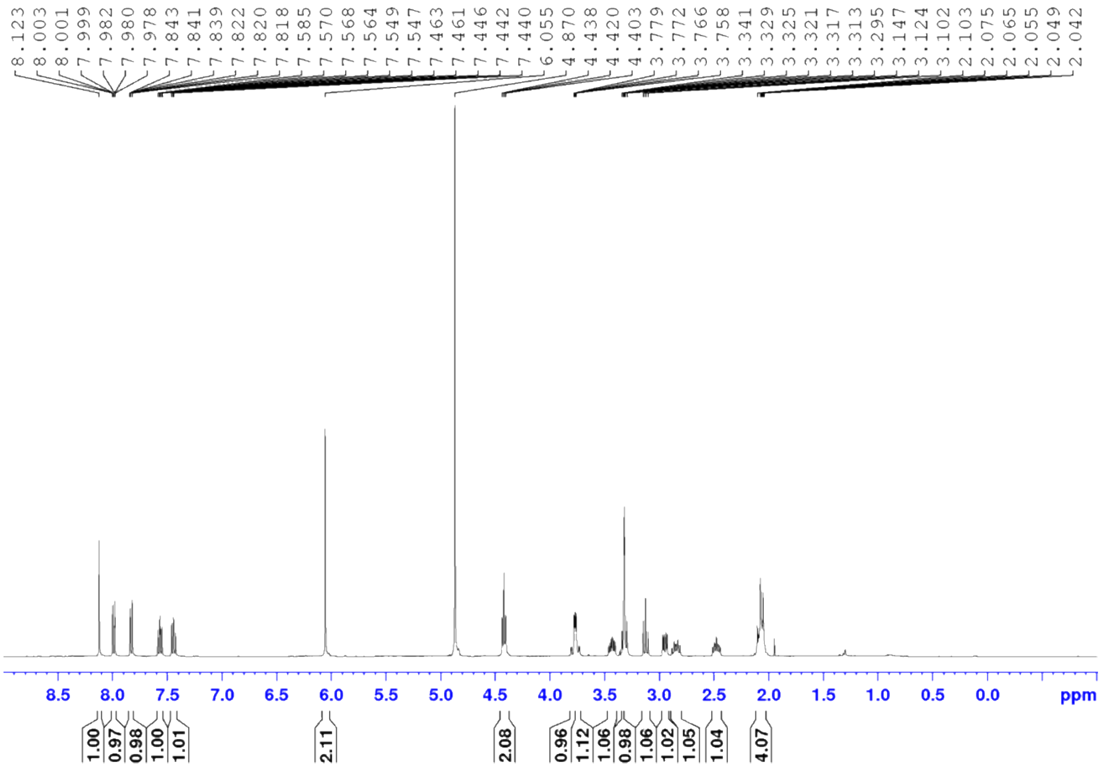

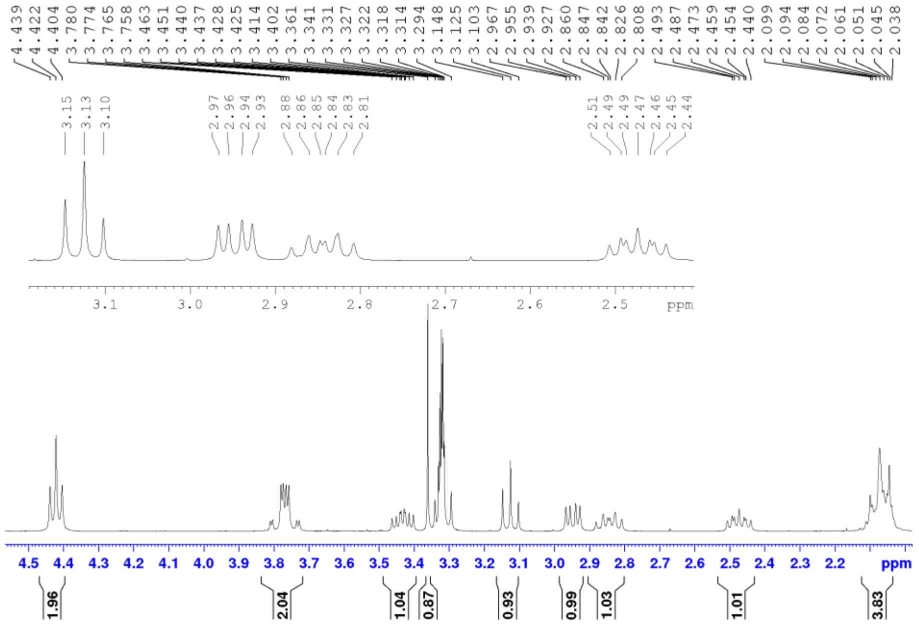

^{13}C-NMR spectra of compound **12a** (MeOD, 100.61 MHz)

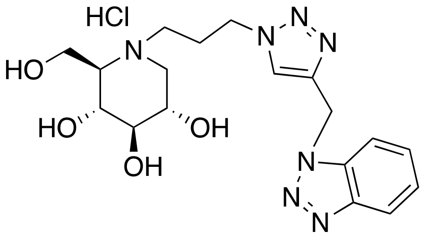

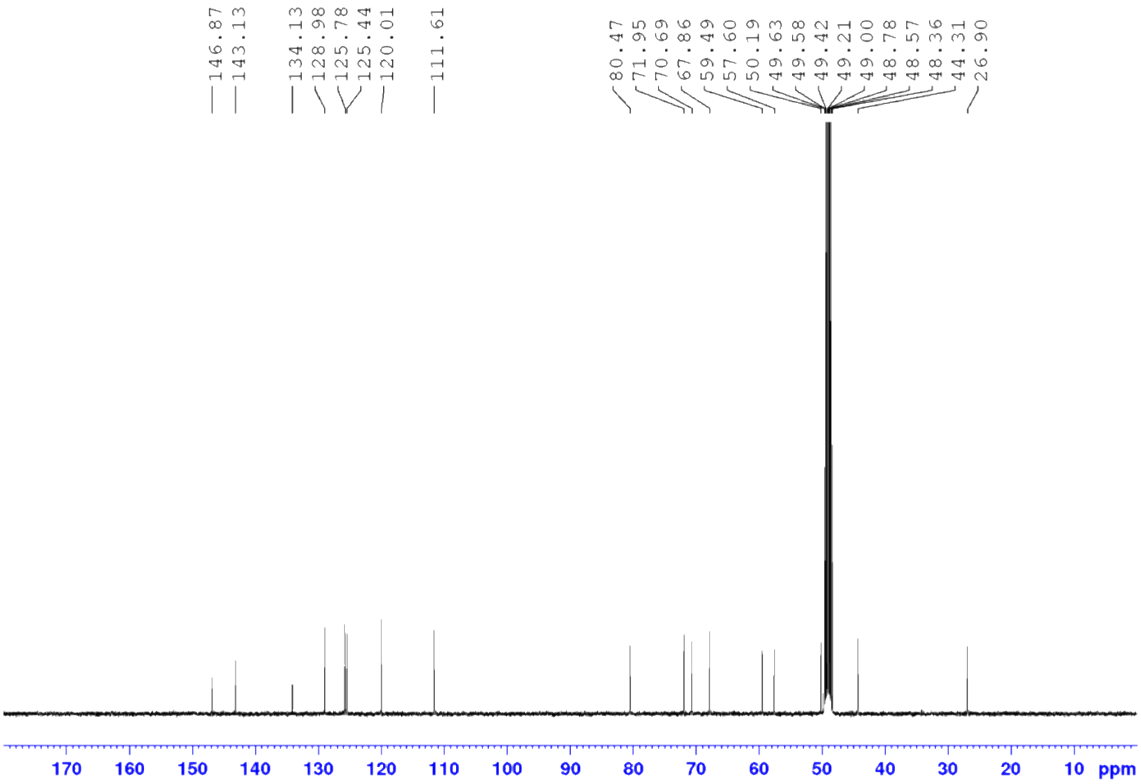

^{1}H-NMR spectra of compound **12b** (MeOD, 400.13 MHz)

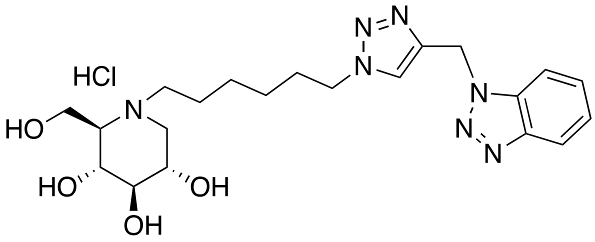

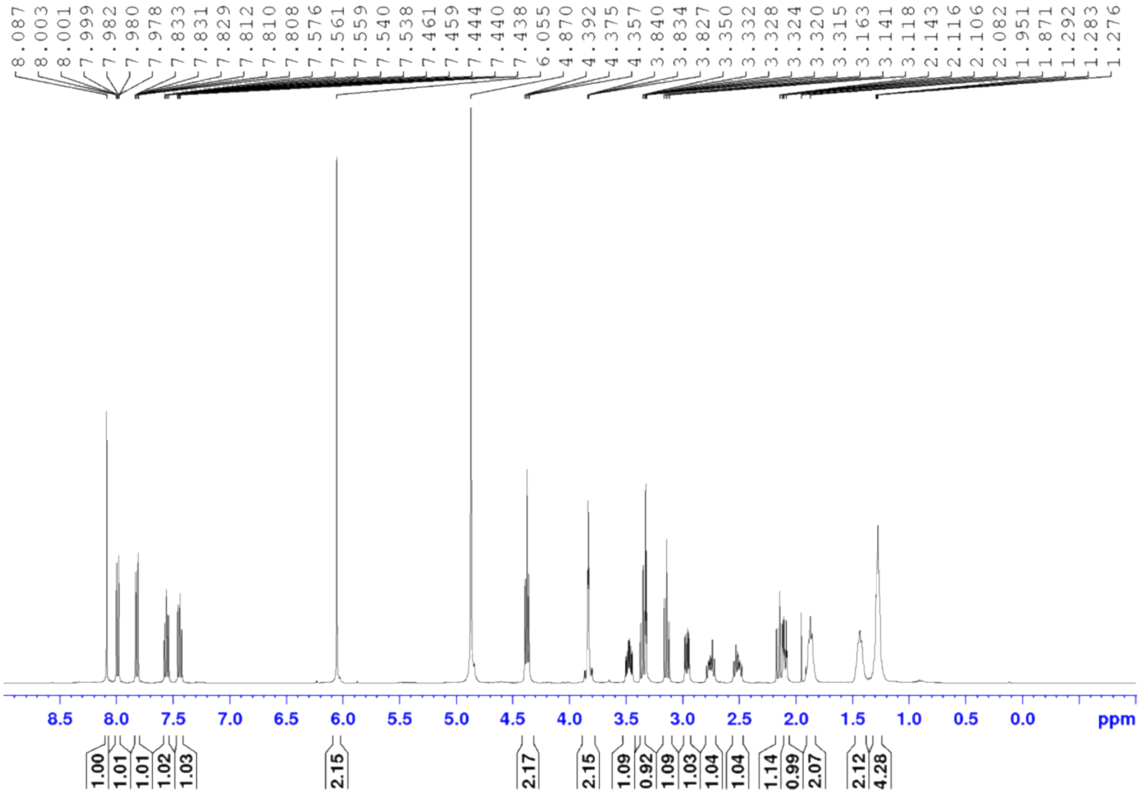

^{13}C-NMR spectra of compound **12b** (MeOD, 100.61 MHz)

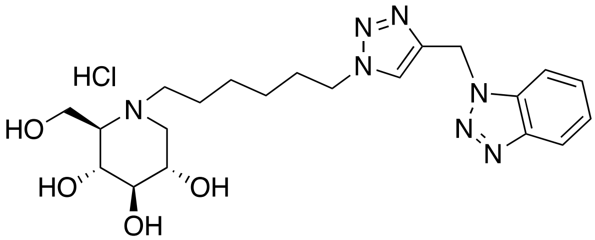

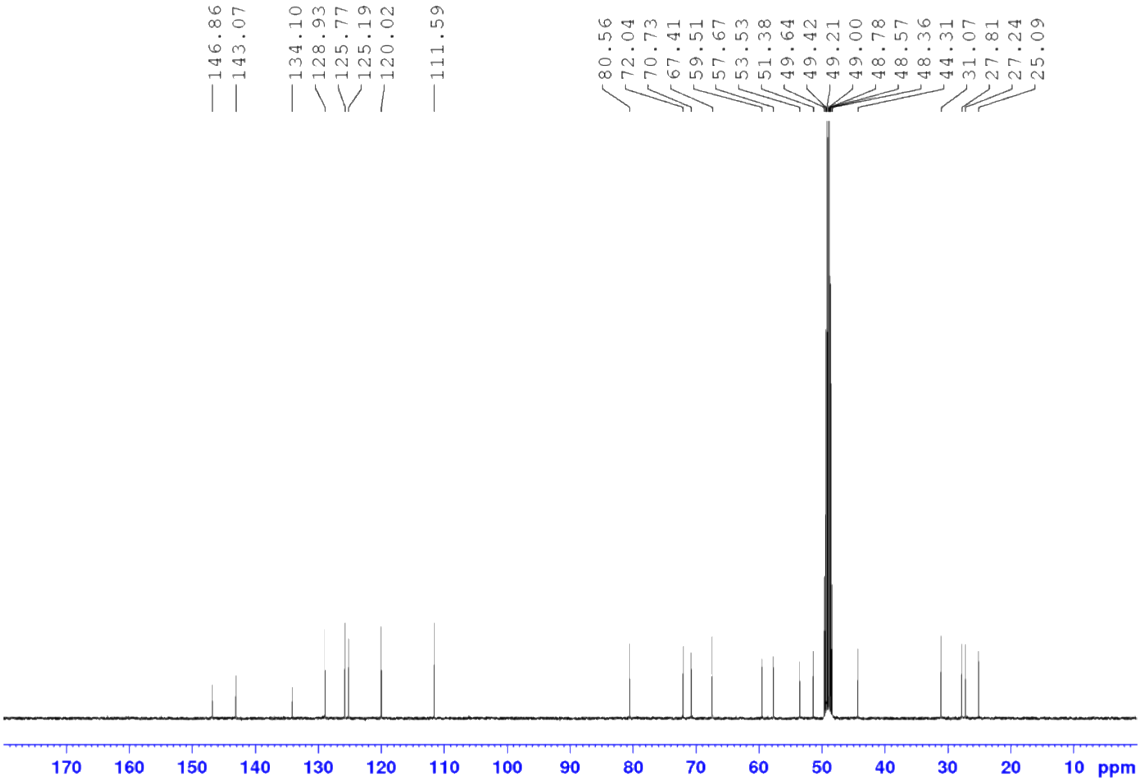

^{1}H-NMR spectra of compound **12c** (MeOD, 400.13 MHz)

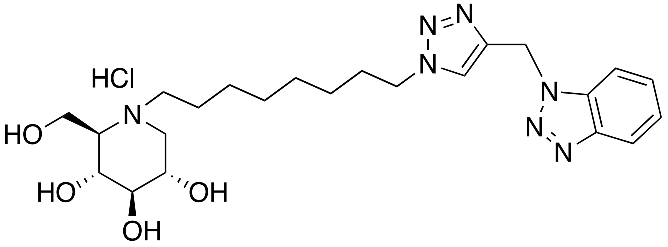

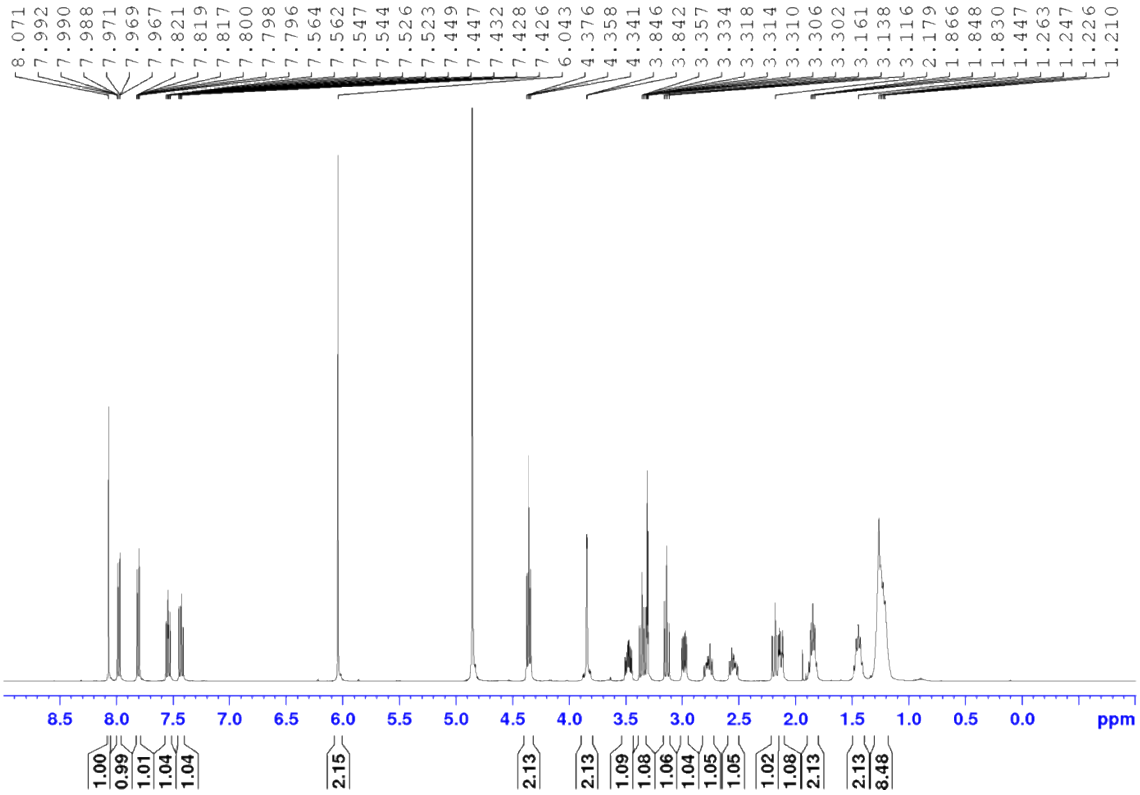

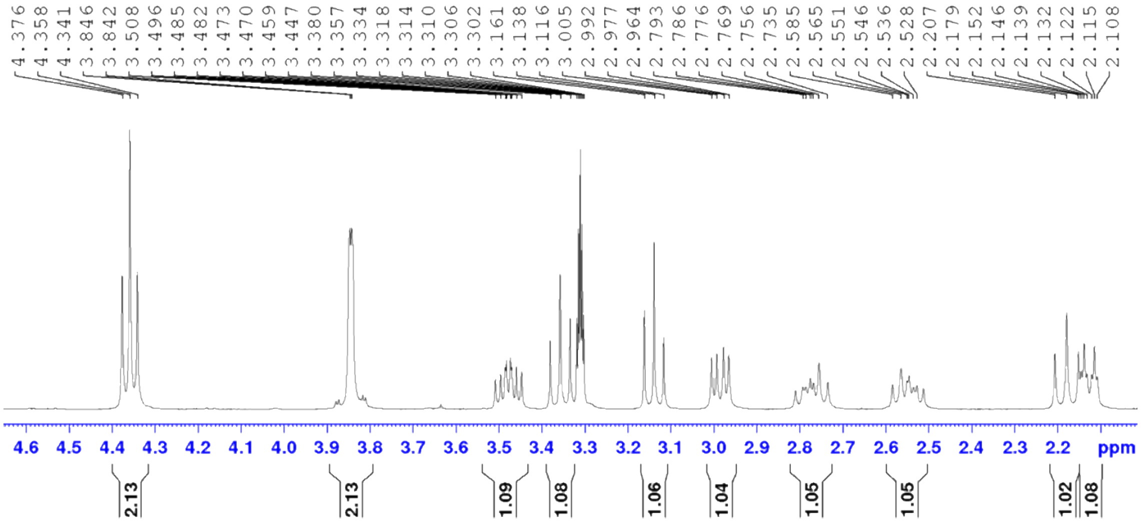

^{13}C-NMR spectra of compound **12c** (MeOD, 100.61 MHz)

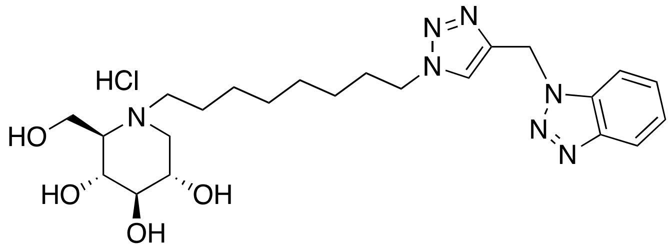

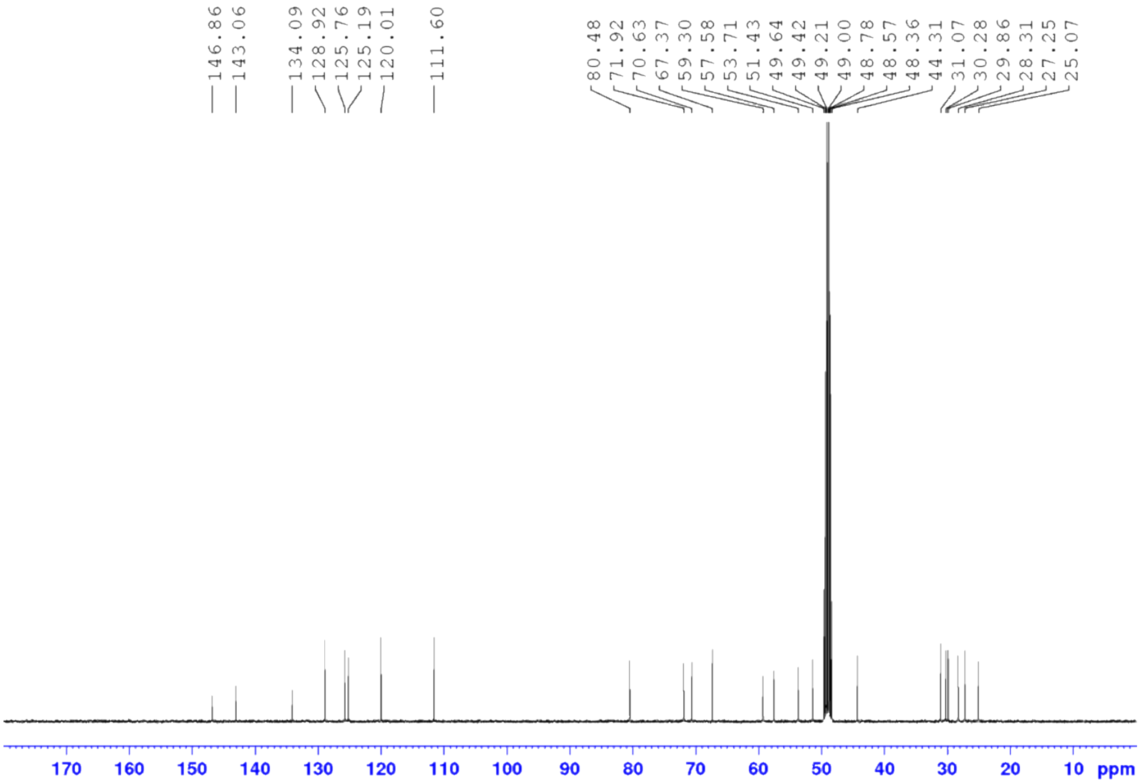

^{1}H-NMR spectra of compound **12d** (MeOD, 400.13 MHz)

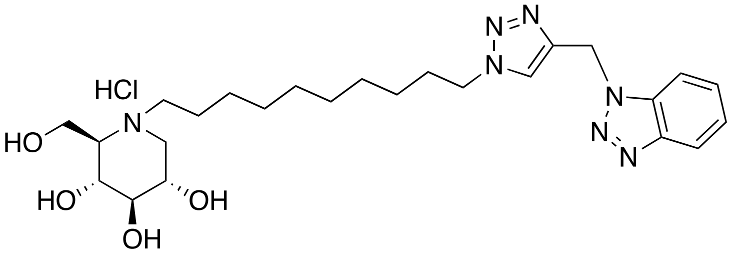

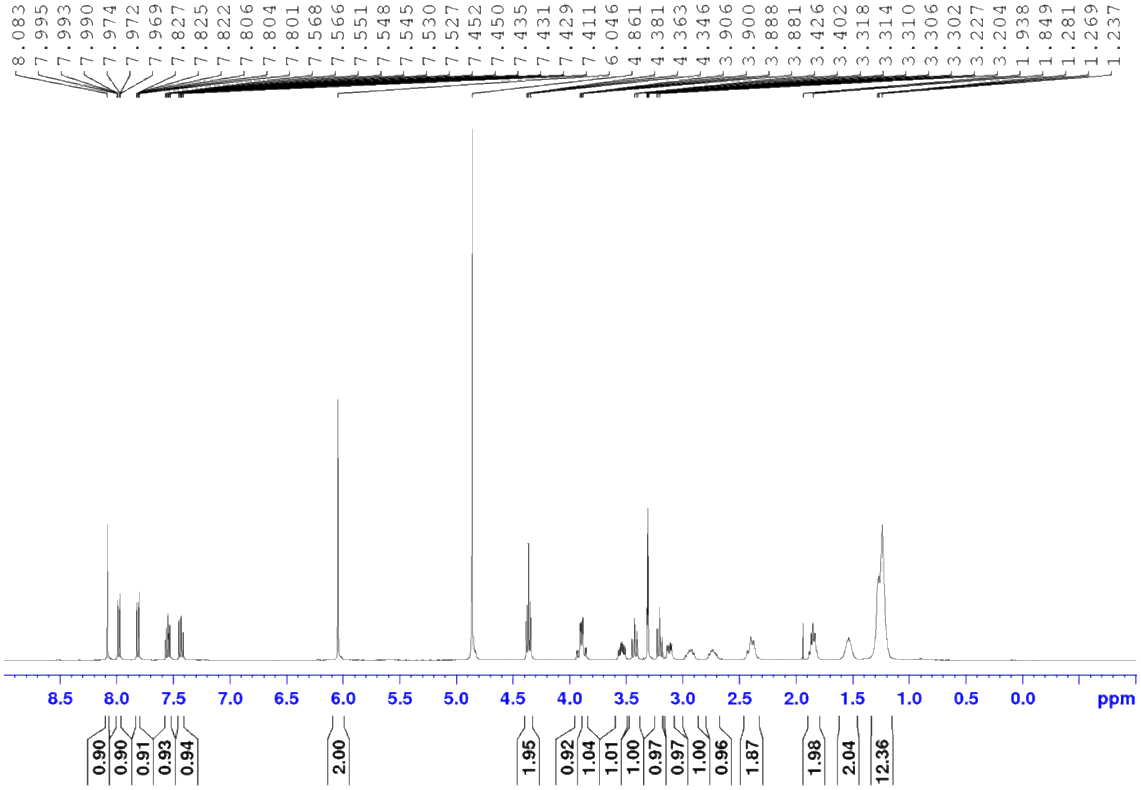

^{13}C-NMR spectra of compound **12d** (MeOD, 100.61 MHz)

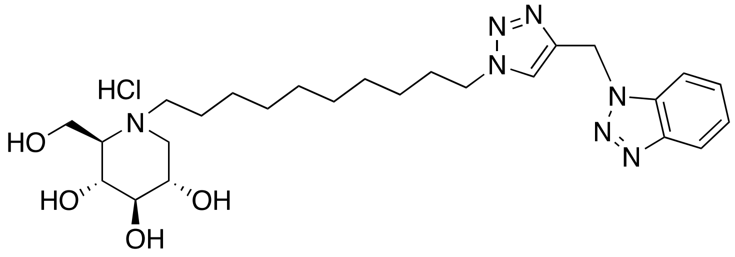

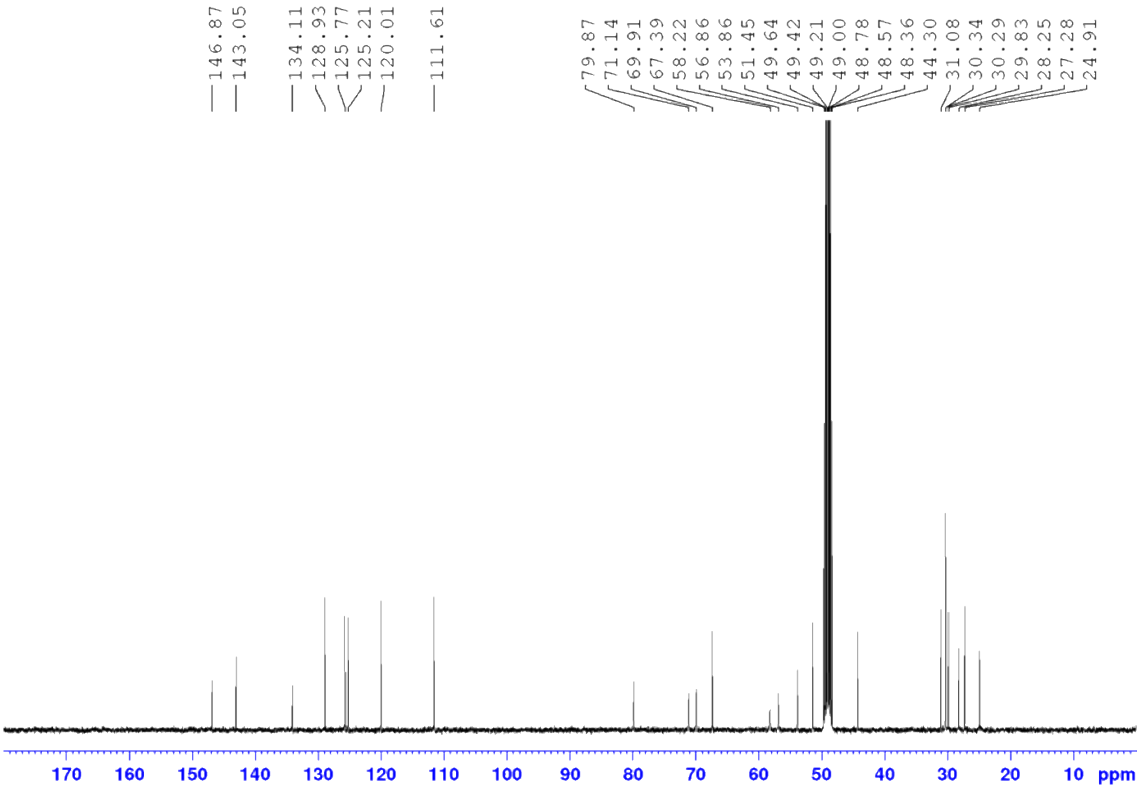

Supplement: Supplemental Material [file IENZ_A_2117912_SM9124.pdf]
